# Distinctive microbial community and genome structure in coastal seawater from a human-made port and nearby offshore island in northern Taiwan facing the Northwestern Pacific Ocean

**DOI:** 10.1371/journal.pone.0284022

**Published:** 2023-06-09

**Authors:** Chi-Yu Shih, Shiow-Yi Chen, Chun-Ru Hsu, Ching-Hsiang Chin, Wei-Chih Chiu, Mei-Hung Chang, Lee-Kuo Kang, Cing-Han Yang, Tun-Wen Pai, Chin-Hwa Hu, Pang-Hung Hsu, Wen-Shyong Tzou

**Affiliations:** 1 Bachelor Degree Program in Marine Biotechnology, National Taiwan Ocean University, Keelung, Taiwan; 2 Taiwan Ocean Genome Center, National Taiwan Ocean University, Keelung, Taiwan; 3 Departent of Bioscience and Biotechnology, National Taiwan Ocean University, Keelung, Taiwan; 4 Center of Excellence for the Oceans, National Taiwan Ocean University, Keelung, Taiwan; 5 Biotools Co., Ltd., New Taipei City, Taiwan; 6 Department of Computer Science and Information Engineering, National Taipei University of Technology, Taipei, Taiwan; 7 Department of Computer Science and Engineering, National Taiwan Ocean University, Keelung, Taiwan; University of the Philippines Diliman, PHILIPPINES

## Abstract

Pollution in human-made fishing ports caused by petroleum from boats, dead fish, toxic chemicals, and effluent poses a challenge to the organisms in seawater. To decipher the impact of pollution on the microbiome, we collected surface water from a fishing port and a nearby offshore island in northern Taiwan facing the Northwestern Pacific Ocean. By employing 16S rRNA gene amplicon sequencing and whole-genome shotgun sequencing, we discovered that Rhodobacteraceae, Vibrionaceae, and Oceanospirillaceae emerged as the dominant species in the fishing port, where we found many genes harboring the functions of antibiotic resistance (ansamycin, nitroimidazole, and aminocoumarin), metal tolerance (copper, chromium, iron and multimetal), virulence factors (chemotaxis, flagella, T3SS1), carbohydrate metabolism (biofilm formation and remodeling of bacterial cell walls), nitrogen metabolism (denitrification, N2 fixation, and ammonium assimilation), and ABC transporters (phosphate, lipopolysaccharide, and branched-chain amino acids). The dominant bacteria at the nearby offshore island (Alteromonadaceae, Cryomorphaceae, Flavobacteriaceae, Litoricolaceae, and Rhodobacteraceae) were partly similar to those in the South China Sea and the East China Sea. Furthermore, we inferred that the microbial community network of the cooccurrence of dominant bacteria on the offshore island was connected to dominant bacteria in the fishing port by mutual exclusion. By examining the assembled microbial genomes collected from the coastal seawater of the fishing port, we revealed four genomic islands containing large gene-containing sequences, including phage integrase, DNA invertase, restriction enzyme, DNA gyrase inhibitor, and antitoxin HigA-1. In this study, we provided clues for the possibility of genomic islands as the units of horizontal transfer and as the tools of microbes for facilitating adaptation in a human-made port environment.

## Introduction

As interference and pollution by humans have continued to deteriorate our environment, many studies have focused on the pollution of rivers and seawater by investigating the microbiome to explore the composition of microorganisms in the water as a means for inferring their effects on public health and the environment. For example, researchers discovered the presence of multidrug resistance genes in bacteria in the Ganges River in India [1]. Metagenomics analysis demonstrated the disease-causing bacterial pathogens *Burkholderia*, *Shigella*, and *Salmonella* in the downstream metropolitan sewage discharge area of São Pedro, Brazil [2]. Researchers in China also found metal tolerance genes and drug resistance genes in bacteria collected from mangrove sediments on Hainan Island [3] and the Pearl River Delta Dawan District [4], China. Some research has also focused on changes in microbiota and potential bioremediation in coastal areas and commercial harbors [5–9].

Fishing ports are unique in that the sources of pollution are numerous. The pollution could be derived from domestic sewage, waste oil and sewage discharged from ships, and wastewater discharged from the surrounding fish market and restaurants. Some fishing ports in Taiwan have both economic and recreational functions, and the environment of the fishing ports recently raised concern due to their pollution [10]. The Badouzi Fishing Port in Keelung, Taiwan, is located at the northern tip of Taiwan, facing the East China Sea and the Northwestern Pacific Ocean. In contrast to the Badouzi Fishing Port, the offshore Heping Island is a recreational park without any fishing or economic activity. This research aims to investigate the microbiomes in the seawater of these two adjacent locations. By employing both 16S rRNA gene amplicon and whole-genome shotgun sequencing, we discovered the differences in the microbiomes in these two locations and identified the potential disease-causing bacteria with antibiotic resistance genes and metal tolerance genes. Network analysis also uncovered the coexistence and mutual-exclusion relationship in the microbial community as the response of the microbial consortia to pollution. Microbial genomes from the fishing port also revealed genomic islands potentially capable of horizontal transfer. This study is the first one for revealing microbial community and its associated genome structure in coastal seawater in northern Taiwan facing the Northwestern Pacific Ocean.

## Materials and methods

### Sample collection and DNA extraction

**Fig 1:**
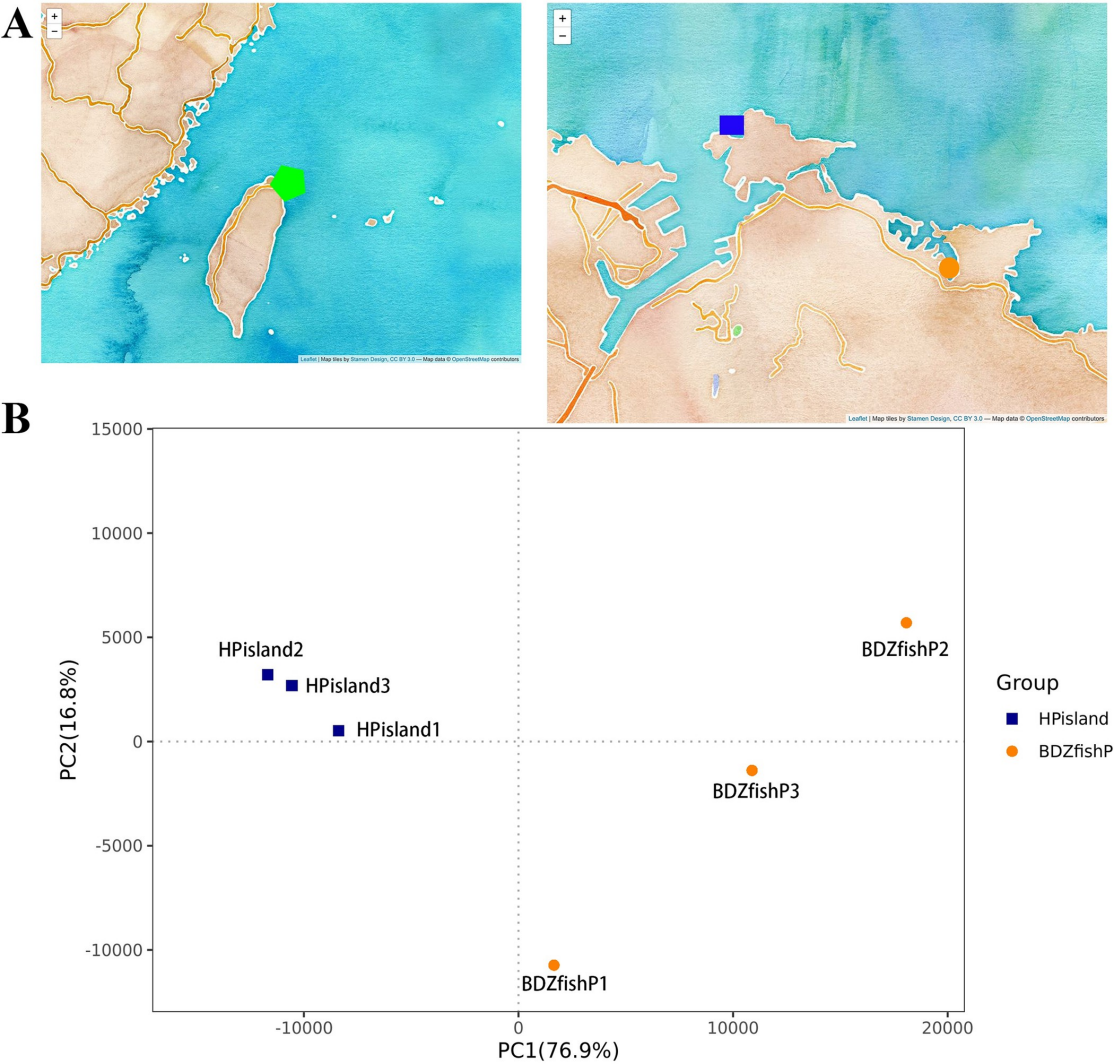
Map of the seawater sampling sites. (A) The sampling sites (in green pentagon) are in northern Taiwan facing the Northwestern Pacific Ocean. Sampling points in a fishing port (Badouzi Fishing Port in orange circles) and nearby offshore island (Heping Island in blue boxes) are shown (map was made from open source https://github.com/Wesely/Taiwan-Python-Map). (B) Principal component analysis (PCA) based on OTU level for the microbiota composition of all samples (PC1 = 76.9%, PC2 = 16.8%). Heping Island (HPisland) and Badouzi Fishing Port (BDZfishP) are indicated in blue boxes and orange circles, respectively.

Heping Island and Badouzi Fishing Port are located at the northeastern point of Taiwan, facing the East China Sea in the Northwestern Pacific Ocean (Fig 1A, S1 and S2 Figs in S1 File). The distance between Heping Island and Badouzi Fishing Port is approximately 4 kilometers. Coastal surface seawater was collected on Heping Island (25.16194 N, 121.76296 E) and Badouzi Fishing Port (25.14120 N, 121.79287 E) in triplicate at different locations on the mornings of July 8 and July 9, 2021, respectively (N = 3 for sampling in Heping Island and Badouzi Fishing Port). Seawater was passed through a 1.2 μm filter first to remove debris and grainy particles and then further passed through a 0.47 μm filter to collect the prokaryotic cells. The filters were stored at -20°C until further processing. Total genomic DNA from samples was extracted using the column-based method (QIAamp PowerFecal DNA Kit, Qiagen). All the samples were processed separately.

### 16S rRNA gene amplicon sequencing and analysis

For 16S rRNA gene sequencing, the V3-V4 region was amplified by a specific primer set (341F: 5’-CCTACGGGNGGCWGCAG-3’, 806R: 5’-GACTACHVGGGTAT CTAATCC-3’) [11], and the sequencing library was prepared according to the 16S Metagenomic Sequencing Library Preparation procedure (Illumina). The library was sequenced on an Illumina MiSeq platform, and paired 300‐bp reads were generated. Demultiplexing was carried out based on barcode identification. Raw amplicon paired-end reads were assembled using FLASH (v1.2.11) [12]. Low-quality reads (Q <20) were discarded in the QIIME (v1.9.1) pipeline [13]. A read was truncated if the quality score of three consecutive bases was < Q20; the resulting read was retained in the dataset only if it was at least 75% of the original length (using split_libraries_fastq.py script in QIIME [14]). UCHIMES was employed to filter sequence chimeras [15, 16]. The effective tags were clustered at 97% sequence identity to generate operational taxonomic units (OTUs) by using the UPARSE [17] function in the USEARCH (v7.0.1090) pipeline [18]. For each representative sequence, the RDP classifier (v2.11) algorithm [19] was employed to annotate taxonomy classification based on the information retrieved from SILVA (an on-line resource for quality checked and aligned ribosomal RNA sequence data, https://www.arb-silva.de/) (138.1) [20, 21] with an 80% minimum confidence threshold. To analyze the sequence similarities among different OTUs, multiple sequence alignment was conducted by using PyNAST software (v1.2) [22] against the core-set dataset in the Silva database.

To normalize the variations in sequence depth across samples, OTU abundance information was rarefied to the minimum sequence depth using the QIIME script (single_rarefaction.py). Subsequent analysis of alpha and beta diversities was performed using the normalized data. The relative abundance and evenness accounting for diversity were evaluated by the Shannon and Simpson indices using the QIIME pipeline. A rarefaction curve was constructed by a random selection of a certain amount of sequencing data of each sample to represent the number of observed species [23]. For beta diversity, T-SNE was performed using the R package Rtsne [24].

### Whole-genome shotgun sequencing and analysis

Total genomic DNA from samples was extracted using the column-based method (QIAamp PowerFecal DNA Kit, Qiagen). DNA degradation and potential contamination were monitored on 1% agarose gels. DNA quantification was checked using a Qubit® dsDNA Assay Kit in a Qubit® 4.0 Fluorometer (Life Technologies, CA, USA). A total amount of 1 μg DNA per sample was used as input material for the library preparations. Sequencing libraries were generated using Illumina Nextera DNA Flex Library Prep (Illumina, USA) following the manufacturer’s recommendations, and index codes were added to attribute sequences to each sample. The library quality was assessed on the Qubit 4.0 Fluorometer and a Qsep100TM system. Subsequently, the library was sequenced on an Illumina NovaSeq platform, and paired 150 bp reads were generated.

The original data obtained by high-throughput sequencing (Illumina NovaSeq 6000 platform) were transformed into raw sequenced reads by CASAVA base calling and stored in FASTQ format. The obtained raw paired-end reads were filtered by Trimmomatic (v0.38) [25] to discard low-quality reads and trim adaptor sequences and to eliminate poor-quality bases. The obtained high-quality data (clean reads) were used for subsequent analyses. Bowtie2 (v2.3.4.1) [26] was used to filter out the contaminating sequences. The filtered reads were then assembled using MEGAHIT (v1.1.3) [27]. Prodigal (v2.6.3) [28] was used to predict the open reading frames (ORFs) from the assembled contigs with lengths > 500 bps. ORFs were filtered to remove those shorter than 100 bps. A nonredundant gene catalog was constructed using CD-HIT (v4.7) with 95% identity [29, 30]. Filtered reads from each sample were mapped to the initial gene catalog using BWA (v0.7.17-r1188). SAMtools (v1.8) was used to create BAM files [31], and the jgi_summarize_bam_contig_depths script from the MetaBAT2 pipeline [32] was run on all BAM files to calculate the coverage for each sample. MetaBAT2 (v2.12.1) was used to carry out metagenomic binning with a minimum contig length threshold of 2500 bps for generating metagenome-assembled genomes (MAG). The genome percentage completeness and contamination of all bins were assessed using CheckM (v1.1.3) [33]. The obtained unigenes were blasted against the NCBI Refseq [34] database using DIAMOND (v0.9.22.123) [35]. The taxonomy assignment was determined by using the lowest common ancestor (LCA) algorithm. The abundances of each taxonomic group were calculated by summing the abundance of genes annotated to a feature. The constructed nonredundant gene catalog was annotated by several functional databases to assign their function. Gene annotation was conducted by aligning sequences against the Refseq (a database that provides comprehensive, integrated, non-redundant, well-annotated set of sequences, including genomic DNA, transcripts, and proteins, https://www.ncbi.nlm.nih.gov/refseq/) [34], eggNOG (a database of hierarchical, functionally and phylogenetically annotated orthology resource, http://eggnog5.embl.de/) [36], KOfam (a database and web server to assign KEGG Orthologs to protein sequences by homology search against a database of profile hidden Markov models, https://www.genome.jp/tools/kofamkoala/) [37], VFDB (a database of virulence factors of bacterial pathogens, http://www.mgc.ac.cn/VFs/) [38], CARD (a database of resistance genes, their products and associated phenotypes, https://card.mcmaster.ca/) [39], NCyc (a database for metagenomic profiling of nitrogen cycling genes, https://github.com/qichao1984/NCyc) [40], dbCAN2 (a database for carbohydrate-active enzymes, https://bcb.unl.edu/dbCAN2/) [41], and BacMet (a database of bacterial genes that are experimentally confirmed to confer resistance to metals and/or antibacterial biocides, http://bacmet.biomedicine.gu.se/) [42] databases using DIAMOND (v0.9.22.123), HMMER (v3.2.1) and other database-specific annotators. Several methods were developed to discover genomic islands in microbial genomes [43], and we employed IslandViewer 4 [44] to search for genomic islands in three assembled genomes. The phylogenomics of MAGs were based on anvi’o workflow [45] and the tree-drawing tool iTOL [46].

Coding sequences from genomic sequences of 8 strains of *Phaeobacter italicus* (RefSeq assembly accession numbers: GCF_019801785.1, GCF_001258055.1, GCF_001404195.1, GCF_900113345.1, GCF_020171225.1, GCF_017743945.1, GCF_019801155.1, and GCF_001251095.1) were downloaded from NCBI. The DNA sequences of genomic islands from the assembled genome (bin) were searched by BLASTN against coding sequences of the 8 strains stated above with the e-value set to 1e-5.

We analyzed the community network structure by analyzing OTU results (abundance value) using CoNet [47]. CoNet deduced the Spearman correlation among all pairs and ran bootstrapping 1000 times. Correlation coefficients larger than 0.8 and p values smaller than 0.05 were retained and shown by Cytoscape [48].

## Results

### Taxonomic analysis using 16S rRNA gene amplicon sequencing reads

**Fig 2:**
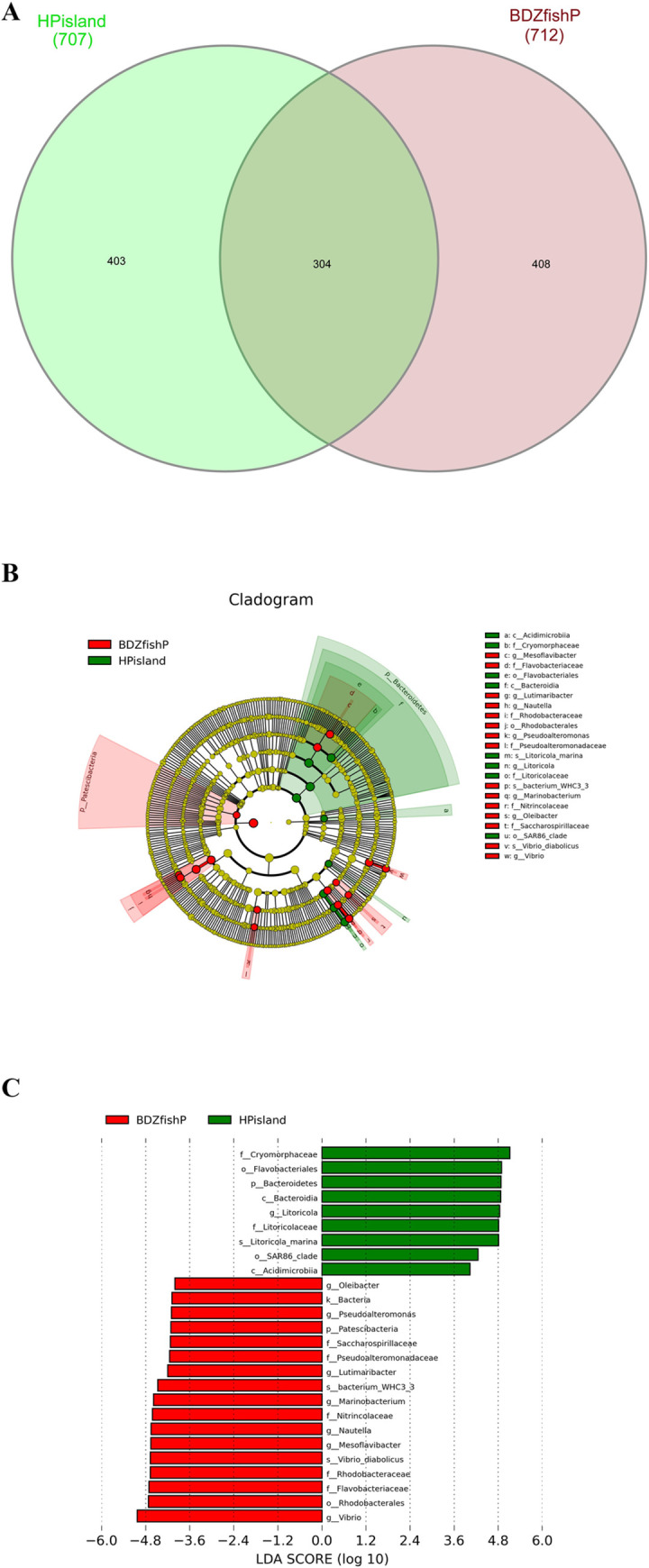
Composition of OTU and LDA analyses. (A) Venn diagrams showing the shared and unique OTUs of the microbiota in the costal seawater of Heping Island and Badouzi Fishing Port. (B) Linear discriminant analysis effect size (LEfSe) cladogram of the microbial composition in seawater of Heping Island and Badouzi Fishing Port. Green and red nodes/shades specify taxa significantly higher in relative abundance in Heping Island and Badouzi Fishing Port, respectively. The taxa abundance is proportional to the circle size. The threshold of the linear discriminant analysis (LDA) score was 4.0. (C) Histogram of the LDA scores showing differential abundances of microbial taxa between Heping Island and Badouzi Fishing Port.

We investigated the microbiome composition by sequencing the 16S rRNA gene V3-V4 regions of bacteria. There were 707 and 712 OTUs collected from 224,703 and 202,954 tags for seawater from Heping Island and Badouzi Fishing Port, respectively (S1 Table in S2 File). The alpha diversity and evenness index indicated that the microbial community in coastal seawater in the fishing port (5.11 of Shannon index and 0.58 of Pielou’s evenness) had higher phylogenetic diversity and evenness than that in the offshore island (4.16 of Shannon index and 0.47 of Pielou’s evenness) (S3-S11 Figs in S1 File, S2 Table in S2 File). PCA showed that the microbial communities in fishing ports and offshore islands were well clustered separately; however, OTUs in fishing ports were more diverse in the second PCA dimension (Fig 1B). A Venn diagram showed that 43% of OTUs were shared between the microbiota in fishing ports and offshore islands (Fig 2A).

We employed SILVA to conduct the taxonomic analysis of OTUs. We found significant differences in the abundance of characteristic microbiota in both sampling sites as determined via LEfSe (Linear discriminant analysis Effect Size, an analysis that can identify genomic biomarkers characterizing statistical differences among biological groups analysis) (Fig 2B and 2C). At the phylum level, Proteobacteria and Bacteroidetes comprised the highest percentages of OTUs in the seawater from Heping Island (50.5% and 43.5%, respectively) and Badouzi Fishing Port (65.3% and 28.7%, respectively) at the phylum level. The sums of the OTU percentages of Proteobacteria and Bacteroidetes from the two sampling locations were the same (94.0%), indicating that the increase or decrease in these two phyla could account for the major difference in the bacterial community in seawater from Heping Island and Badouzi Fishing Port. Compared with the community structure of Heping Island, the decrease in Bacteroidetes was on par with the increase in Proteobacteria at Badouzi Fishing Port (for Proteobacteria, 65.3%-50.5% = 14.8%; for Bacteroidetes, 28.7–43.5% = -14.8%).

**Fig 3:**
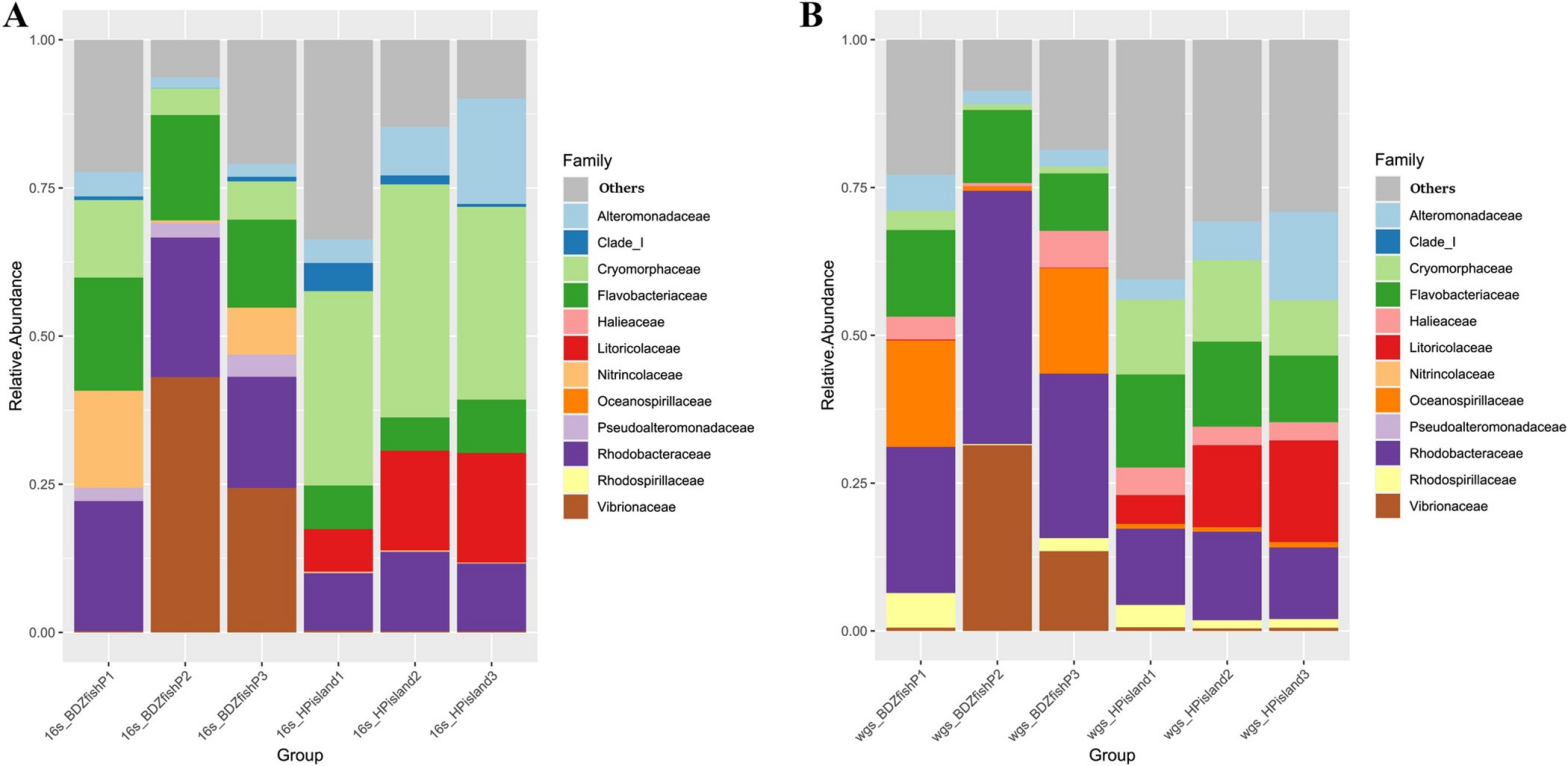
Microbial composition (in relative abundance) in Badouzi Fishing Port and Heping Island. The distribution of major contributing families (top ten) based on the analysis of (A) 16S rRNA gene amplicon sequencing and (B) whole-genome shotgun sequencing.

At the family level (S3 Table in S2 File), at Heping Island, Cryomorphaceae (34.9%), Litoricolaceae (14.2%), Rhodobacteraceae (11.5%), Alteromonadaceae (10.0%), Flavobacteriaceae (7.3%), Clade_I (2.2%), and Actinomarinaceae (2.1%) were the top families of the bacterial community; at Badouzi Fishing Port, Vibrionaceae (22.6%), Rhodobacteraceae (21.4%), Flavobacteriaceae (17.2%), Nitrincolaceae (8.2%), Cryomorphaceae (8.0%), and Pseudoalteromonadaceae (2.8%) were the top families of the bacterial community (Fig 3A).

These data indicated that in comparison with Heping Island in terms of the percentage of community structure, the changes in the populations of Cryomorphaceae (Bacteroidetes, 26.9% down), Litoricolaceae (Proteobacteria, 14.1% down), and Alteromonadaceae (Proteobacteria, 7.3% down) were commensurate with the changes in the populations of Vibrionaceae (Proteobacteria, 22.3% up), Rhodobacteraceae (Proteobacteria, 9.9% up), Flavobacteriaceae (Bacteroidetes, 9.9% up) and Nitrincolaceae (Proteobacteria, 8.1% up) at Badouzi Fishing Port.

### Taxonomic analysis using whole-genome shotgun sequencing

The numbers of paired-end raw reads sequenced were 173 and 198 million for seawater from Heping Island and Badouzi Fishing Port, respectively (S4 Table in S2 File). A total of 2,352,957 contigs (with 947,562 contigs longer than 500 nt), 1,359,362 ORFs, and 409,712 unique genes were generated. Finally, the numbers of unique genes annotated by Refseq and eggNOG was 225,025 and 301,223, respectively, for seawater from Heping Island. For seawater from Badouzi Fishing Port, the numbers of unique genes annotated by Refseq and eggNOG was 230,953 and 305,820, respectively.

At the phylum level, Proteobacteria and Bacteroidetes were still the two most abundant phyla. For Heping Island, 55.0% and 38.3% of microbes detected belong to Proteobacteria and Bacteroidetes, respectively. For Badouzi Fishing Port, 80.3% and 18.0% of microbes detected belonged to Proteobacteria and Bacteroidetes, respectively.

At the family level, at Heping Island, the unclassified family was the most abundant (16.1%), followed by Flavobacteriaceae (13.8%), Rhodobacteraceae (13.3%), Litoricolaceae (12.0%), Cryomorphaceae (11.9%), Alteromonadaceae (8.3%), Halieaceae (3.6%), and Rhodospirillaceae (2.2%) (Fig 3B, S5 Table in S2 File).

At Badouzi Fishing Port, Rhodobacteraceae was the most abundant (31.8%), followed by Vibrionaceae (15.2%), Flavobacteriaceae (12.3%), Oceanospirillaceae (12.2%), unclassified (7.6%), Alteromonadaceae (3.8%), Halieaceae (3.5%), Rhodospirillaceae (2.7%) and Pseudoalteromonadaceae (2.5%) (Fig 3B, S5 Table in S2 File).

If we compared the percentages of the dominant taxa at Heping Island with those at Badouzi Fishing Port, we observed the decreases (in percentage) of Litoricolaceae (11.9% decrease), Cryomorphaceae (10.1% decrease), and Alteromonadaceae (4.6% decrease) and the increases of Rhodobacteraceae (18.5% increase), Vibrionaceae (14.6% increase), and Oceanospirillaceae (11.4% increase).

### General functional analysis using Kyoto Encyclopedia of Genes and Genomes (KEGG)

We investigated the functional categories by employing KEGG analysis using ORFs as the input sequences and found 334 pathways associated with ORFs. Pathways common to both collections with high abundance included (top 20, Heping Island and Badouzi Fishing Port, respectively) ko01100 Metabolic pathways (29.5%, 23.2%), ko01110 Biosynthesis of secondary metabolites (13.3%, 9.8%), ko01120 Microbial metabolism in diverse environments (7.5%, 6.3%), ko01240 Biosynthesis of cofactors (6.4%, 4.6%), ko01230 Biosynthesis of amino acids (5%, 3.7%), ko01200 Carbon metabolism (4.5%, 3.3%), ko02010 ABC transporters (2.5%, 2.8%), ko02020 Two-component system (2%, 2.6%), ko03010 Ribosome (3.7%, 2%), ko00230 Purine metabolism (2.7%, 2%), ko00240 Pyrimidine metabolism (2.4%, 1.7%), ko02024 Quorum sensing (1.7%, 1.7%), ko00190 Oxidative phosphorylation (1.9%, 1.4%), ko00630 Glyoxylate and dicarboxylate metabolism (1.5%, 1.3%), ko00260 Glycine, serine and threonine metabolism (1.6%, 1.3%), ko00620 Pyruvate metabolism (1.5%, 1.2%), ko01212 Fatty acid metabolism (1.6%, 1.2%), ko00280 Valine, leucine and isoleucine degradation (1.2%, 1.1%), ko01250 Biosynthesis of nucleotide sugars (1.7%, 1%), and ko00860 Porphyrin and chlorophyll metabolism (1.1%, 1%).

We also found several pathways unique to sampling sites in Badouzi Fishing Port: ko01053 Biosynthesis of siderophore group nonribosomal peptides (9.6 folds compared with that in Heping Island), ko05110 Vibrio cholerae infection (33.3 folds), ko05150 Staphylococcus aureus infection (112.3 folds) and ko05135 Yersinia infection (210.2 folds). Furthermore, we also found several pathways unique to sampling sites in Heping Island: ko00944 Flavone and flavonol biosynthesis (10.4 folds compared with that in Badouzi Fishing Port) and ko04144 Endocytosis (6.3 folds).

### Nitrogen metabolism

**Fig 4:**
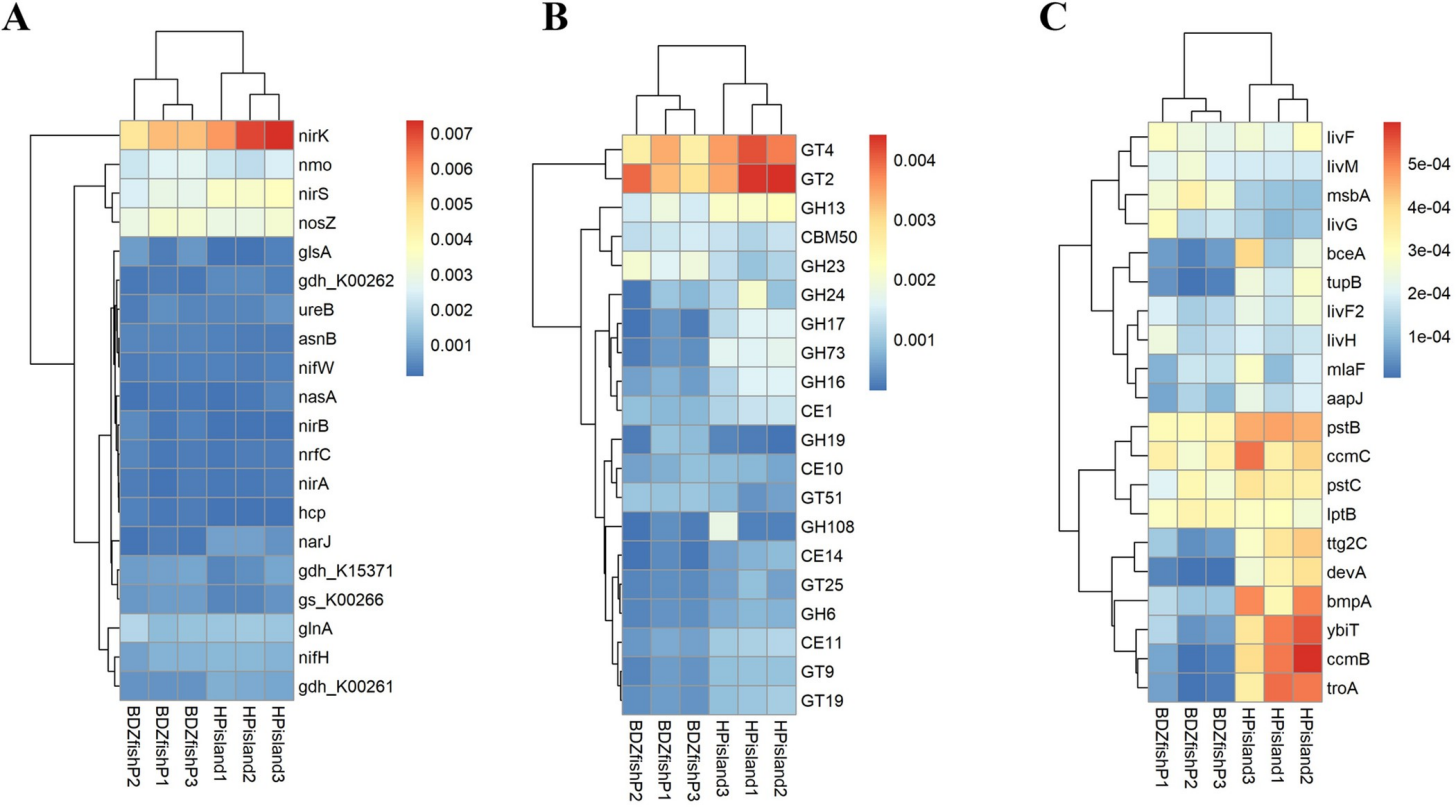
Heatmaps showing the relative abundances of genes of metabolisms. (A) nitrogen cycling metabolism, (B) carbohydrate metabolism, and (C) ABC transporters. Samples were collected from three sites in Badouzi Fishing Port (BDZfishP1, BDZfishP2, BDZfishP3) and Heping Island (HPisland1, Hpisland2, Hpisland3).

We employed the NCyc database to understand nitrogen metabolism. *nirK*, *nirS*, *nosZ*, and *nmo* were more abundant at both Heping Island and Badouzi Fishing Port than other genes (Fig 4A, S6 Table in S2 File). (t-test *p* = 2.2e-16, 1.4e-10, 1.4e-10, and 3.2e-07 for *nirK*, *nirS*, *nosZ*, and *nmo*, respectively).

*nirK*, *nirS*, *nosZ*, and *nmo* were all responsible for denitrification. Denitrification consists of four enzymatic steps, starting from nitrate and producing the intermediates nitrite, nitric oxide, and nitrous oxide. *nirK* and *nirS* catalyzed the reduction of nitrite. *nosZ* catalyzed the reduction of nitrous oxide. *nmo* is an enzyme (nitronate monooxygenase) that catalyzes the oxidative denitrification of alkyl nitronates by using oxygen (O_2_).

We found that most of the *nirK* gene sequences were affiliated with Flavobacteriaceae, Litoricolaceae, and Alteromonadaceae at Heping Island and with Vibrionaceae, Rhodobacteraceae, and Oceanospirillaceae at Badouzi Fishing Port (S12 Fig in S1 File, S6 Table in S2 File). Most of the *nirS* gene sequences were affiliated with Cryomorphaceae, Litoricolaceae, Alteromonadaceae at Heping Island and with Rhodobacteraceae at Badouzi Fishing Port (S13 Fig in S1 File). Most of the *nosZ* and *nmo* gene sequences were affiliated with Litoricolaceae at Heping Island and Rhodobacteraceae at Badouzi Fishing Port (S14 and S15 Figs in S1 File, S6 Table in S2 File).

We also found genes responsible for ammonium assimilation (*glnA*, *gdh*) at both Heping Island (Cryomorphaceae) and Badouzi Fishing Port (Rhodobacteraceae) (S16-S18 Figs in S1 File, S6 Table in S2 File). A gene responsible for N_2_ fixation (*nifH*) was also found in bacteria collected from both Heping Island (Flavobacteriaceae) and Badouzi Fishing Port (Oceanospirillaceae) (S19 Fig in S1 File, S6 Table in S2 File). Other genes responsible for N_2_ fixation were also found in bacteria collected from both Heping Island (*nifW* from Alteromonadaceae, Litoricolaceae and Rhodobacteraceae) and Badouzi Fishing Port (*nifKD* from Vibrionaceae).

Moreover, *narJ* at Heping Island (Flavobacteriaceae), a chaperone protein for the assembly of nitrate reductase (*narG*, a nitrate reductase that can convert nitrate to nitrite) and molybdenum cofactor, was also observed.

### Carbohydrate metabolism

We employed the dbCAN2 database to search for genes responsible for carbohydrate metabolism. In 452 families of bacteria found in the sampling sites, 183 families of them were demonstrated to have genes responsible for degrading, modifying, or creating glycosidic bonds (40.5%). Both GT2 and GT4 were highly abundant at Heping Island and Badouzi Fishing Port (Fig 4B, S7 Table in S2 File). GT stands for the family of glycosyltransferases that catalyze the transfer of sugar moieties from activated donor molecules to specific acceptor molecules in the biosynthesis of disaccharides, oligosaccharides, and polysaccharides. GT2 includes cellulose synthase, chitin synthase, and hyaluronan synthase, which are involved in biofilm and capsule components in bacteria. GT4 includes sucrose synthase and sucrose-phosphate synthase, which are involved in sucrose and sucrose 6-phosphate, respectively [49].

We also found that GH13 gene sequences were abundant at Heping Island. GH stands for a family of glycoside hydrolases that can hydrolyze the glycosidic bonds between two or more carbohydrates or between a carbohydrate and a noncarbohydrate moiety. GH13 includes alpha-amylase, pullulanase, sucrose phosphorylase, and glucosidase, which can degrade phytoplankton-derived glucan. GH23 was abundant at Badouzi fishing port. GH23 is a peptidoglycan lyase important for the remodeling of bacterial peptidoglycan (PG) in the cell wall of bacteria and its pathogenicity.

We found that most of the GT2 gene sequences were affiliated with Rhodobacteraceae at Heping Island and Badouzi Fishing Port (S20 Fig in S1 File, S7 Table in S2 File). GT4 gene sequences were affiliated with Cryomorphaceae and Alteromonadaceae at Heping Island and with Rhodobacteraceae and Vibrionaceae at Badouzi Fishing Port (S21 Fig in S1 File, S7 Table in S2 File). At Heping Island, GH13 gene sequences were affiliated with Cryomorphaceae and Flavobacteriaceae (S22 Fig in S1 File, S7 Table in S2 File). At Badouzi Fishing Port, GH23 gene sequences were affiliated with Rhodobacteraceae and Vibrionaceae (S23 Fig in S1 File, S7 Table in S2 File).

### ABC transporters and phosphotransferase

To understand ABC transporter metabolism in the collected bacteria, we employed the ggnog database and found 3,129 genes. Gene sequences for *pstB* (ATP-binding protein for phosphate transport), *ccmC* (export of heme to the periplasm for the biogenesis of c-type cytochromes), *pstC* (phosphate transport system permease protein), and *lptB* (lipopolysaccharide export) were abundant at both Heping Island and Badouzi Fishing Port (Fig 4C, S8 Table in S2 File). Gene sequences for *bmpA* (sugar transport system substrate-binding protein), *ybiT* (ATP-binding protein), *ttg2C* (phospholipid or cholesterol transport), *ccmB* (permease involved in cytochrome c biogenesis), *troA* (manganese/zinc/iron transport system substrate-binding protein), and *devA* (ABC exporter, ATP-binding subunit) were abundant at Heping Island. *msbA* (ATP-dependent lipid A-core flippase), *livM* (permeases for branched-chain amino acid transport) and *livG* (permeases for branched-chain amino acid transport) were abundant at Badouzi Fishing Port.

At Badouzi Fishing Port and Heping Island, we found that most of the *pstB* sequences were affiliated with Rhodobacteraceae. *pstC* sequences were affiliated with Litoricolaceae at Heping Island and Vibrionaceae at Badouzi Fishing Port. *ccmC* sequences were affiliated with Litoricolaceae and Alteromonadaceae at Heping Island and Oceanospirillaceae at Badouzi Fishing Port. *lptB* sequences were affiliated with Litoricolaceae and Cryomorphaceae at Heping Island and with Rhodobacteraceae and Vibrionaceae at Badouzi Fishing Port.

At Heping Island, *bmpA*, *ybiT*, and *troA* were affiliated with Litoricolaceae, Cryomorphaceae, and Thermoactinomycetaceae, respectively. At Badouzi Fishing Port, *msbA* and *livM* and *livG* were affiliated with Rhodobacteraceae, and *livG* was affiliated with Gammaproteobacteria.

Phosphotransferase system (PTS) was utilized by bacteria to uptake sugar as the source energy. We also employed eggNOG database and found 53 genes encoding phosphotransferase. Gene sequences for *pstN* (Phosphotransferase system mannitol fructose-specific IIA domain) were abundant at both Heping Island and Badouzi Fishing Port. Gene sequences for *thrB* (Phosphotransferase enzyme family) was abundant at Heping Island. *cmtB*, *frwC* (Phosphotransferase system mannitol fructose-specific IIA domain), *celC* (Phosphotransferase system cellobiose-specific component IIA), *crr* (Phosphotransferase system IIA components), *chpT* (Histidine phosphotransferase C-terminal domain), and *fruA* (Phosphotransferase system fructose-specific component IIB) were abundant at Badouzi Fishing Port.

At Badouzi Fishing Port, we found that most of the *celC*, *cmtB*, *crr*, *fruA*, *frwC*, and *pstN* sequences were affiliated with Vibrionaceae. *chpT and pstN* sequences were affiliated with Rhodobacteraceae. *pstN* sequences was also affiliated with Oceanospirillaceae.

At Heping Island, *pstN* and *thrB* were affiliated with Litoricolaceae. *pstN* sequences was also affiliated with Rhodobacteraceae.

### Antibiotic resistance genes

We found 68 genes conferring antibiotic resistance in the collected bacterial samples by employing the CARD database. We found 452 families of bacteria in the sampling sites and 41 families of them have AR genes of presence (9.1%). While 33.8% of 68 AR genes were found in the sea water of Heping Island, 97.1% of AR genes were found in the sea water of Badouzi Fishing Port.

**Fig 5:**
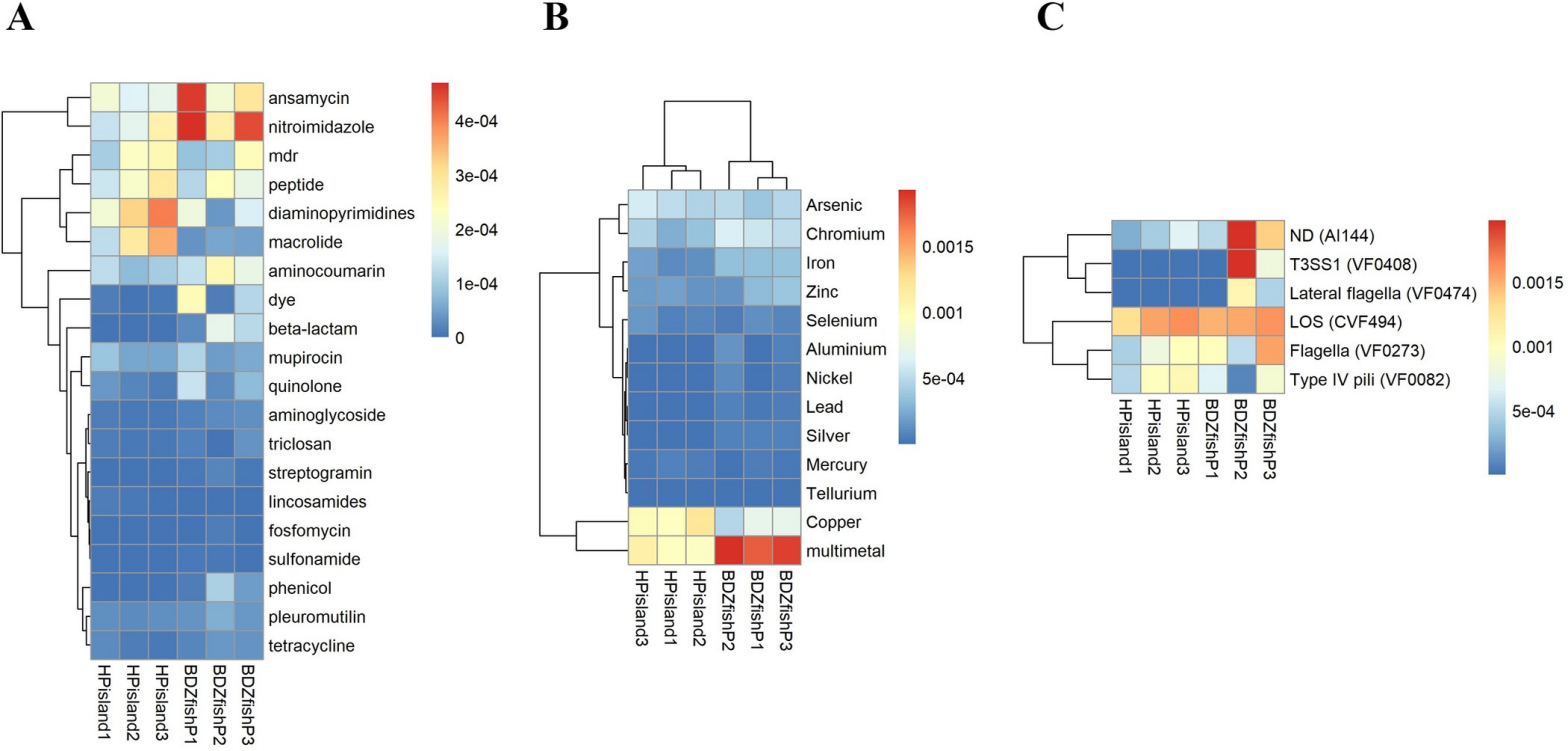
Heatmaps showing the relative abundances of genes conferring resistance to antibiotics and metals and encoding virulence factors. (A) Resistance to 20 categories of antibiotics and (B) Metal tolerance to 13 categories of metals and (C) Virulence factors. Samples were collected from three sites in Badouzi Fishing Port (BDZfishP1, BDZfishP2, BDZfishP3) and Heping Island (HPisland1, Hpisland2, Hpisland3).

Regarding resistance mechanisms, “antibiotic efflux” was the most prevalent (35 genes, 51.5%). Thirteen genes (19.1%) functioned through “antibiotic inactivation”, 12 genes (17.6%) through antibiotic target alteration, 7 genes (10.3%) through antibiotic target replacement, and one gene through antibiotic target protection. We also categorized potential bacteria-resistant antibiotics into 20 antibiotic categories. Genes conferring resistance to diaminopyrimidines and macrolides were abundant in the seawater of Heping Island (Fig 5A, S9 Table in S2 File). Genes responsible for the resistance of diaminopyrimidines include *dfrA26*, *dfrA17*, *dfrA15*, *dfrA3*, and *dfrA20* (dihydrofolate reductase, antibiotic target replacement). Genes responsible for the resistance to macrolides include *macB* (ABC transporter), *oleC* (ABC transporter), *mexW* (RND-type membrane protein of the efflux complex MexVW-OprM), *CpxR* (activation of expression of the RND efflux pump MexAB-OprM), *LpeB* (subunit of the LpeAB efflux pump), and *abeS* (efflux pump of the SMR family). Conversely, genes conferring resistance to ansamycin, nitroimidazole, and aminocoumarin were abundant in the seawater of Badouzi Fishing Port (Fig 5A, S9 Table in S2 File). The gene responsible for the resistance of ansamycin was *rpoB2* (beta-subunit of RNA polymerase, antibiotic target alteration, or replacement). The gene involved in the resistance of nitroimidazole was *msbA* (multidrug resistance transporter, antibiotic efflux). The gene responsible for the resistance of aminocoumarin was *novA* (type III ABC transporter, antibiotic efflux).

At Badouzi Fishing Port, we found that most of the gene sequences conferring ansamycin resistance were affiliated with Vibrionaceae (S31 Fig in S1 File, S9 Table in S2 File). Gene sequences conferring nitroimidazole resistance were affiliated with Rhodobacteraceae, Shewanellaceae, and Oceanospirillaceae (S32 Fig in S1 File, S9 Table in S2 File). Gene sequences conferring aminocoumarin resistance were affiliated with Rhodobacteraceae.

At Heping Island, we found that most of the gene sequences conferring diaminopyrimidine or macrolide resistance were affiliated with Litoricolaceae and Alteromonadaceae (S33 Fig in S1 File, S9 Table in S2 File).

More specifically (S9 Table in S2 File), at Heping Island, *Litoricola lipolytica* (in the family of Litoricolaceae within Gammaproteobacteria) harnessed five antibiotic resistance genes (*CRP*, *Pseudomonas*, *dfrA3*, *Nocardia*, *msbA*). At Badouzi Fishing Port, *Vibrio* within Gammaproteobacteria contained 13 antibiotic resistance genes (*ugd*, *MexF*, *abeS*, *CARB-18*, *catB8*, *CARB-19*, *CRP*, *qacH*, *hmrM*, *FosC2*, *MCR-3*, *mexW* and *catB9*). Rhodobacteraceae within Alphaproteobacteria harbored four antibiotic resistance genes (*msbA*, *novA*, *smeR*, *and ugd*). *Oleibacter marinus* and *Marinobacterium sp*. *AK27* (in the family Oceanospirillaceae within Gammaproteobacteria) contained five (msbA, CRP, dfrA26, acrB, Streptomyces) and three antibiotic resistance genes (*Nocardia*, *MexF*, and *ugd*), respectively.

### Metal tolerance genes

We found 85 genes in the BacMet database involved in metal resistance. In 452 families of bacteria found in the sampling sites, 44 families of them have metal tolerance genes of presence (9.7%). While 51.8% of 85 metal tolerance genes were found in the sea water of Heping Island, 98.8% of metal tolerance genes were found in the sea water of Badouzi Fishing Port.

There were thirteen types of metal resistance (gene number): multimetal (35 genes, channel, enzyme, efflux, transcription regulator, membrane transporter, binding protein), arsenic (9 genes, transcription regulator, chaperone, enzyme, binding protein), aluminum (one gene, enzyme), chromium (3 genes, efflux and enzyme), copper (11 genes, enzyme and transcription regulator), iron (8 genes, enzyme, binding protein, transcription regulator, and membrane protein), lead (one gene, enzyme), mercury (4 genes, membrane transporter and transcription regulator), nickel (3 genes), selenium (2 genes, enzyme), silver (2 genes, efflux and enzyme), tellurium (3 genes), and zinc (5 genes, enzyme, efflux, membrane transporter, binding protein, and transcription regulator) (S10 Table in S2 File).

At Heping Island, we found abundant genes resistant to copper, arsenic, and multimetal (Fig 5B, S10 Table in S2 File). Most of the gene sequences conferring multimetal and arsenic resistance were affiliated with Alteromonadaceae (S34, S35 Figs in S1 File). Gene sequences conferring copper resistance were affiliated with Cryomorphaceae (S36 Fig in S1 File, S10 Table in S2 File).

In the seawater of Badouzi Fishing Port, we found abundant genes conferring resistance to copper, chromium, iron, and multimetal compounds (Fig 5B, S10 Table in S2 File). Most of the gene sequences conferring multimetal resistance were affiliated with Rhodobacteraceae and Vibrionaceae (S34 Fig in S1 File, S10 Table in S2 File). Gene sequences conferring copper resistance were affiliated with Rhodobacteraceae (S36 Fig in S1 File, S10 Table in S2 File). Gene sequences conferring iron resistance were affiliated with Vibrionaceae and Oceanospirillaceae (S37 Fig in S1 File, S10 Table in S2 File). Gene sequences conferring chromium resistance were affiliated with Rhodobacteraceae, Vibrionaceae, and Oceanospirillaceae (S38 Fig in S1 File, S10 Table in S2 File).

More specifically (S10 Table in S2 File), at Heping Island, *Litoricola lipolytica* (in the family Litoricolaceae within Gammaproteobacteria) harbored six metal-tolerance genes. Both *Phaeocystidibacter luteus* (in the family Cryomorphaceae within Flavobacteriales) and Alteromonadaceae within Gammaproteobacteria contained one metal tolerance gene. At Badouzi Fishing Port, *Vibrio* within Gammaproteobacteria contained 24 metal-tolerance genes. *Phaeobacter italicus* (in the family Rhodobacteraceae within Alphaproteobacteria) harbored four metal-tolerance genes. *Marinobacterium stanieri* and *Oleibacter marinus* (in the family Oceanospirillaceae species within Gammaproteobacteria) harbored nine and two metal-tolerance genes, respectively.

### Virulence factors

We searched the virulence factor database (VFDB) for virulence factors of the bacteria under investigation. We found 369 genes belonging to 122 groups. In 452 families of bacteria found in the sampling sites, 102 families of them have virulence genes of presence (22.6%). While 45.3% of 369 virulence genes were found in the sea water of Heping Island, 98.1% of virulence genes were found in the sea water of Badouzi Fishing Port.

At the group level, we discovered that LOS (CVF494, 367 genes) was abundant at both Heping Island and Badouzi Fishing Port; ND (AI144, 278 genes), VF0408 (53 genes) and VF0273 (244 genes) were abundant at Badouzi Fishing Port (Fig 5C, S11 Table in S2 File).

Genes of the LOS (lipooligosaccharide) (CVF494) group bear similarity to those from *Haemophilus influenzae Rd KW20* and encode proteins involved in the structuring and biosynthesis of lipid A-containing complex glycolipids in the outer membranes and associated pathogenicity [50–52]. Genes of the ND (AI144) group bear similarity to those from *Aeromonas hydrophila subsp*. *hydrophila ATCC 7966* and encode proteins responsible for chemotaxis and flagellar synthesis and function. They were demonstrated to be involved in cirrhosis in humans and the invasion and survival of *Aeromonas hydrophila* in *Anguilla japonica* macrophages [53, 54]. Genes of the VF0273 group bear similarity to those from *Pseudomonas aeruginosa PAO1* and encode proteins responsible for swimming motility of bacteria relying on flagellar activity and involved in biofilm formation and pathogenic adaptations [55–57]. Genes of the VF0408 group bear similarity to those from *Vibrio parahaemolyticus RIMD 2210633* and encode proteins for the T3SS1 system. T3SS1 is a type three secretion system (a needle-like structure) and causes the cytotoxicity of host cells, involving the induction of autophagy, cell rounding, and cell lysis [58].

We also found that most of the gene sequences of LOS (CVF494) were affiliated with Bacteroidetes and Alteromonadaceae at Heping Island and with Vibrionaceae and Oceanospirillaceae at Badouzi Fishing Port (S39 Fig in S1 File, S11 Table in S2 File). At Badouzi Fishing Port, ND (AI144) and T3SS1 (VF0408) were affiliated with Vibrionaceae, and Flagella (VF0273) was affiliated with Oceanospirillaceae (S40 and S41 Figs in S1 File, S11 Table in S2 File).

### MAGs (metagenome-assembled genomes) in the coastal seawater and the characterization of metabolic pathways

**Fig 6:**
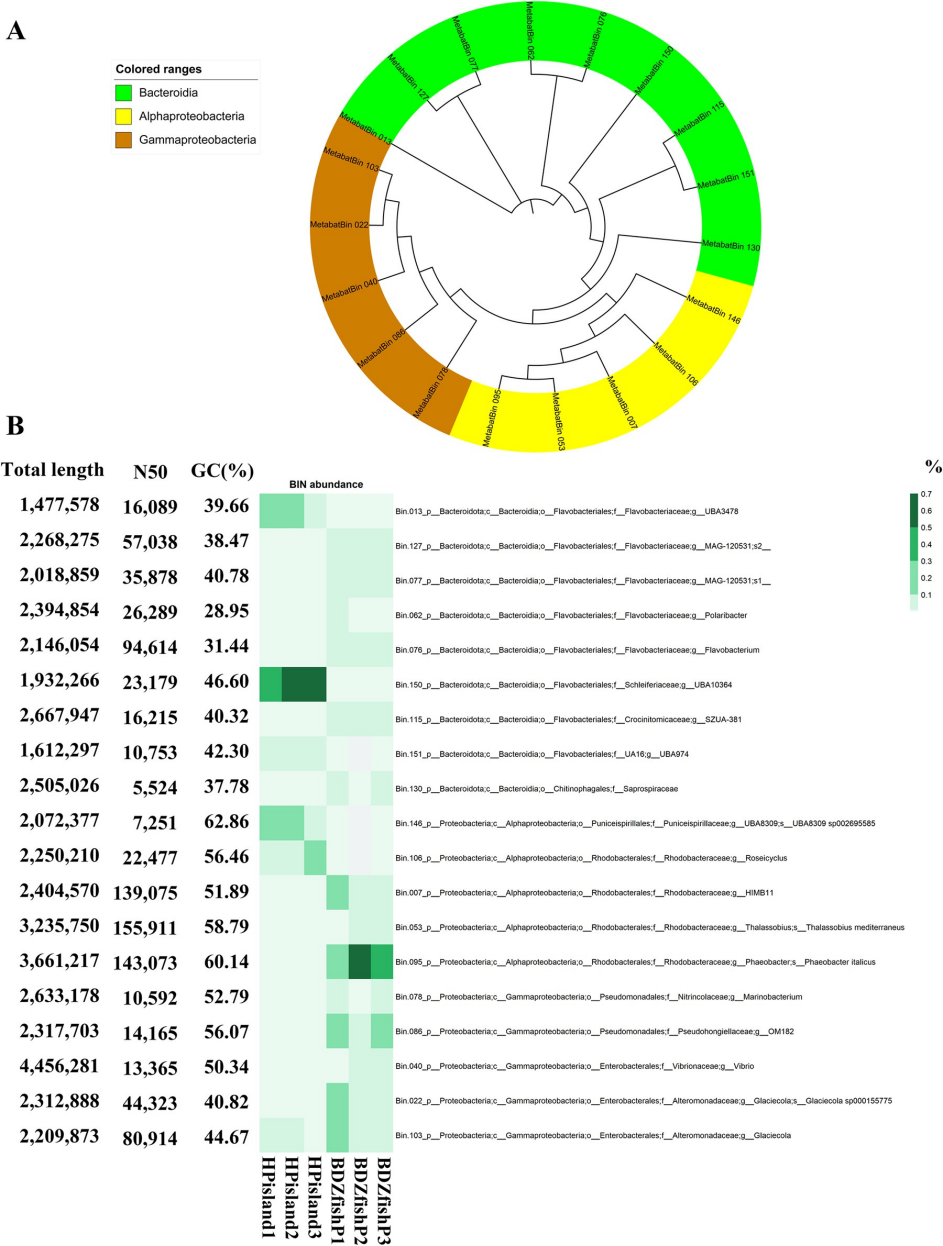
19 metagenome assembled genomes (MAGs) discovered in the coast seawater of Heping Island and Badouzi Fishing Port. (A) Phylogenetic analysis of MAGs. Color codes indicate the taxonomic assignment of “class”. (B) Heatmap showing the abundance of 19 MAGs. Samples were collected from three sites in Badouzi Fishing Port (BDZfishP1, BDZfishP2, BDZfishP3) and Heping Island (HPisland1, HPisland2, HPisland3). Accompanied table includes statistics of MAG including total length (bp), N50 (bp) and GC (%).

We assembled microbial genomes of high quality and abundance collected from coastal surface seawater of Heping Island and Badouzi Fishing Port (completeness larger than 80%, contamination less than 5%), yielding 19 bins. These genomes were affiliated with two phyla of bacteria (Bacteroidota and Proteobacteria) and three major classes (Bacteroidia, Alphaproteobacteria and Gammaproteobacteria) (Fig 6A). The largest genome of Bins in size contains 4.5MBases (genus Vibrio). Genus UBA3478 (Bin.013, Flavobacteriaceae), UBA10364 (Bin.150, Schleiferiaceae) and UBA8309 (Bin.146, Puniceispirillaceae) were abundant in Heping Island. Genus Glaciecola (Bin.022, Alteromonadaceae), HIMB11 (Bin.007, Rhodobacteraceae), Thalassobius (Bin.053, Rhodobacteraceae), Phaeobacter (Bin.095, Rhodobacteraceae), Vibrio (Bin.040, Vibrionaceae) and OM182 (Bin.086, Pseudohongiellaceae) were abundant in seawater of Badouzi Fishing Port (Fig 6B).

**Fig 7:**
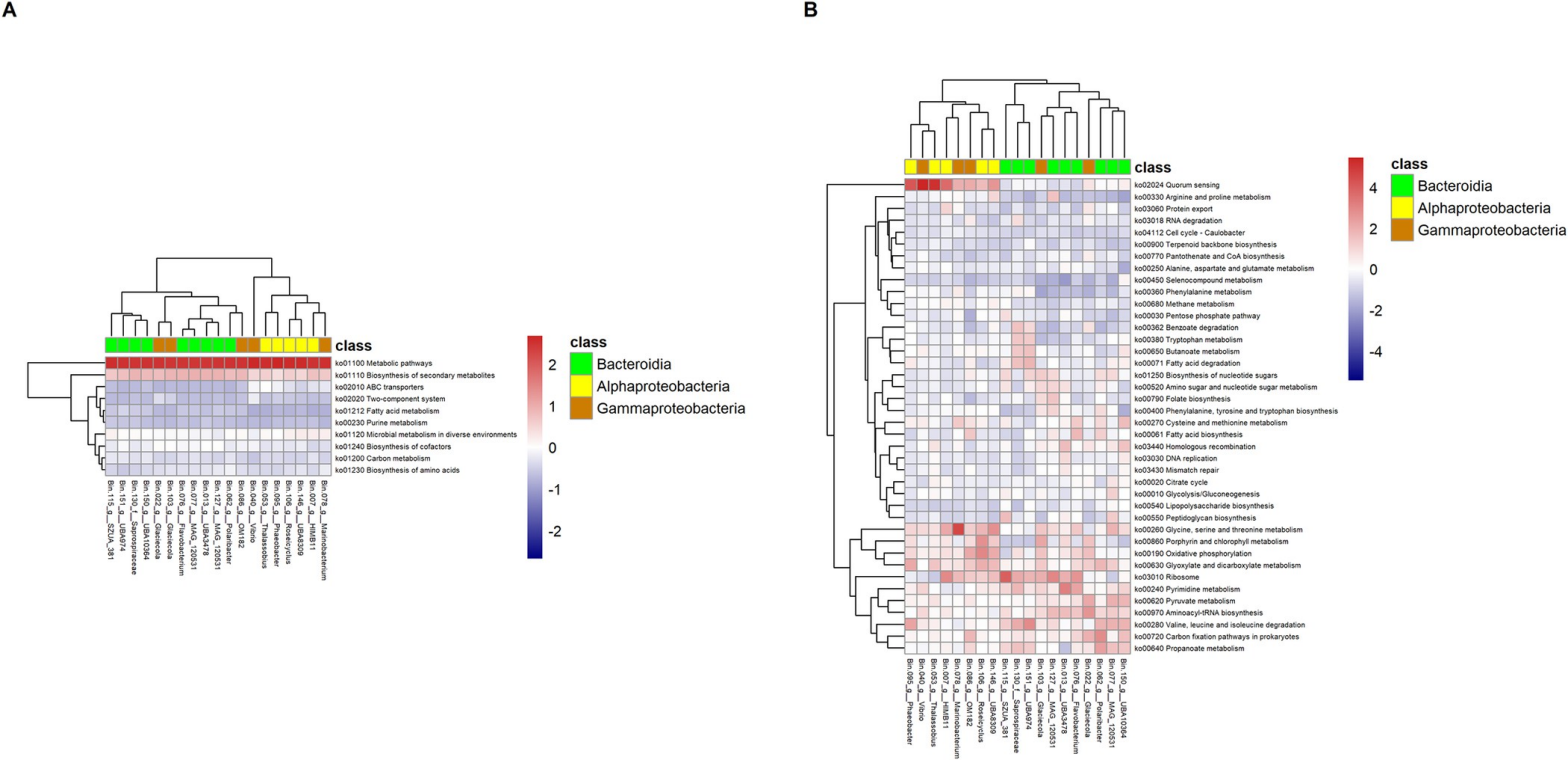
Heatmaps showing the relative abundance of genes with metabolic functions associated with 19 MAGS discovered in the coast seawater of Heping Island. (A) Top ten metabolic functions of high abundance. (B) Other metabolic functions. Color codes indicate the taxonomic assignment of “class”.

**Fig 8:**
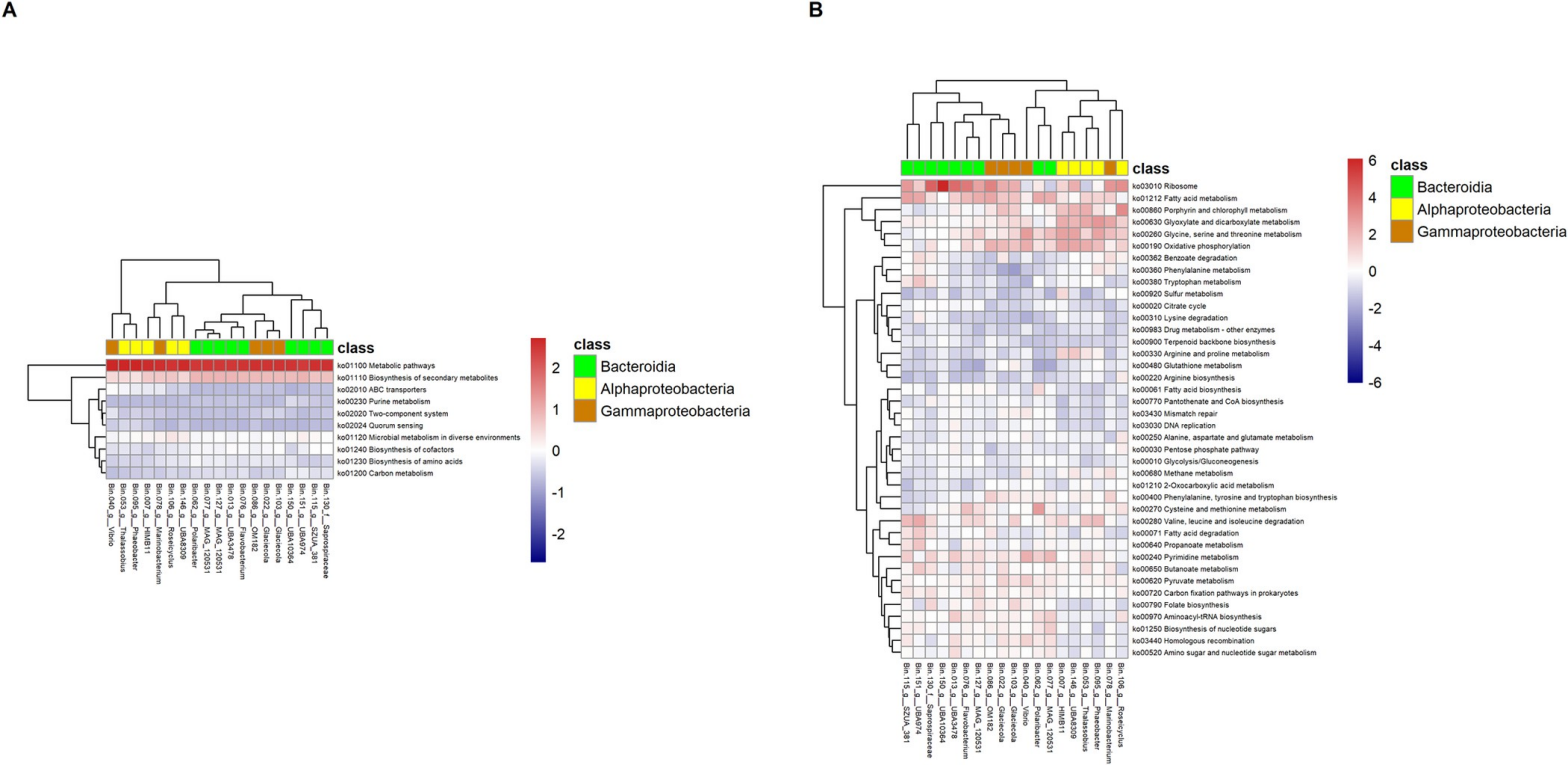
Heatmaps showing the relative abundance of genes with metabolic functions associated with 19 MAGS discovered in the coast seawater of Badouzi Fishing Port. (A) Top ten metabolic functions of high abundance. (B) Other metabolic functions. Color codes indicate the taxonomic assignment of “class”.

We also mapped the metabolic pathway inferred from MAGs (Figs 7 and 8). Overrepresented gene functions in seawater of both sampling sites were metabolic pathways, biosynthesis of secondary metabolites, microbial metabolism in diverse environments, biosynthesis of cofactors, carbon metabolism, biosynthesis of amino acids, ABC transporters, fatty acid metabolism, purine metabolism and two-component system. On the other hand, through clustering of functions, we also found several groups of abundant gene functions associated with specific MAGs. For example, in Heping Island (Fig 7), we discovered that quorum sensing, glycine, serine/threonine metabolism, oxidative phosphorylation, porphyrin/chlorophyll metabolism, and glyoxylate/dicarboxylate metabolism were associated with genus HIMB11 (Bin.007, Rhodobacteraceae), Thalassobius (Bin.053, Rhodobacteraceae), Phaeobacter (Bin.095, Rhodobacteraceae) and UBA8309 (Bin.146, Puniceispirillaceae). Benzoate degradation, tryptophan metabolism, butanoate metabolism and fatty acid degradation were associated with genus SZUA_381 (Bin.115, Crocinitomicaceae), UBA974 (Bin.151, UA16) and Saprospiraceae (Bin.130).

Clustering of functions associated with specific MAGs were also found in Badouzi Fishing Port (Fig 8). For example, oxidative phosphorylation, glycine, serine/threonine metabolism, glyoxylate/dicarboxylate metabolism and porphyrin/chlorophyll metabolism were associated with genus HIMB11 (Bin.007, Rhodobacteraceae), Thalassobius (Bin.053, Rhodobacteraceae), Phaeobacter (Bin.095, Rhodobacteraceae), Roseicyclus (Bin.106, Rhodobacteraceae), Marinobacterium (Bin.078, Nitrincolaceae) and UBA8309 (Bin.146, Puniceispirillaceae). Pyrimidine metabolism, pyruvate metabolism, carbon fixation pathways, aminoacyl-tRNA biosynthesis, biosynthesis of nucleotide sugars and homologous recombination were associated with genus Vibrio (Bin.040, Vibrionaceae), Polaribacter (Bin.062, Flavobacteriaceae), Flavobacterium (Bin.076, Flavobacteriaceae), MAG_120531 (Bin.077, Flavobacteriaceae), MAG_120531 (Bin.127, Flavobacteriaceae) and Glaciecola (Bin.022, Alteromonadaceae).

### Genomic islands (GIs) of the abundant bacteria Rhodobacteraceae in the coastal seawater of the fishing port

**Fig 9:**
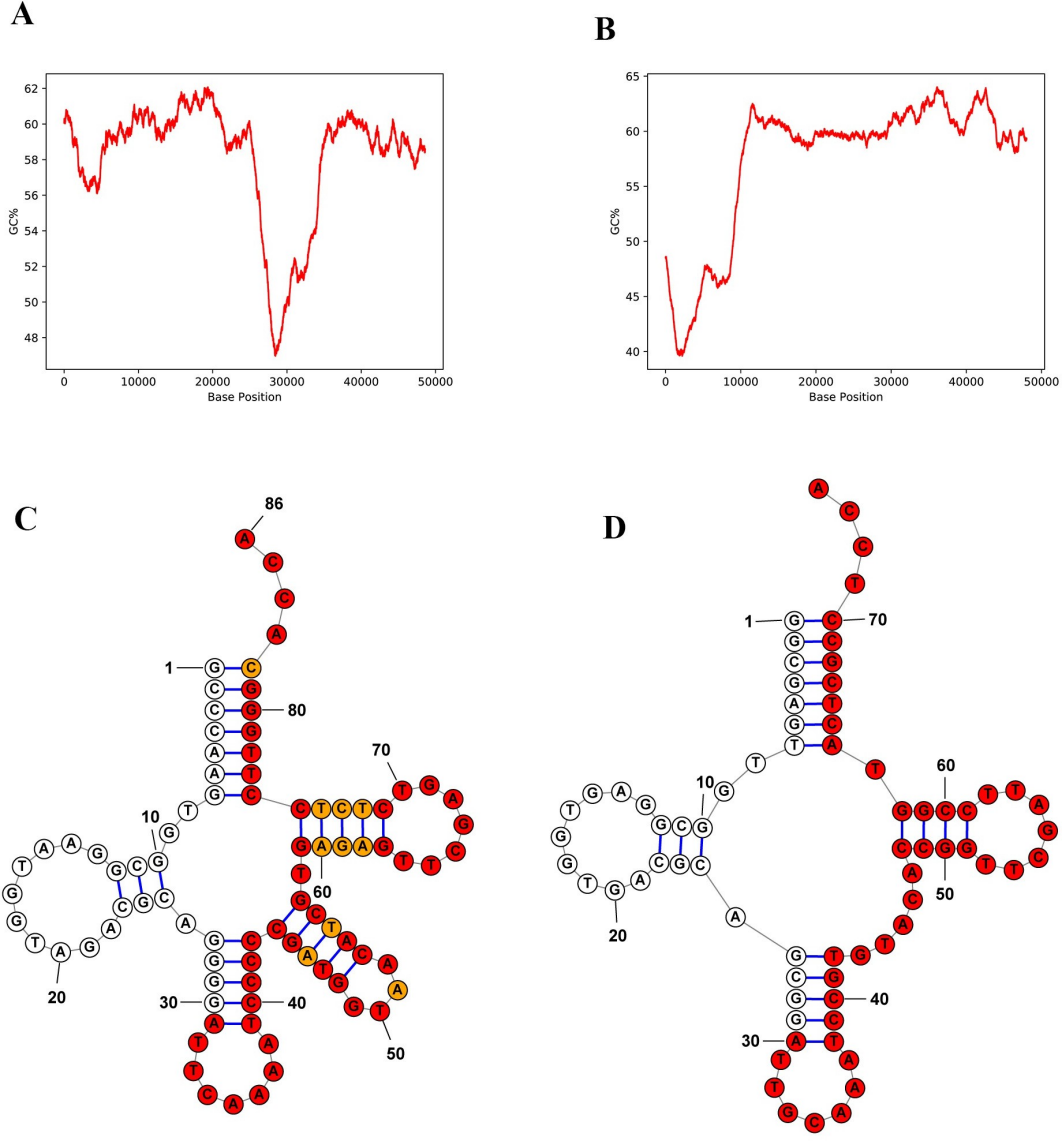
GC contents and direct repeat sequences on the tRNA structures of two genomic islands in the genome of *Phaeobacter italicus* discovered in the costal seawater of Badouzi Fishing Port. (A, B) GC content as a percentage, employing a scanning window of 3000 bps. (C, D) tRNA secondary structures and the direct repeat sequences on one side of the genomic islands (perfect repeat nucleotides are colored in red circles, and imperfect repeat nucleotides are colored in orange). The tRNAs in C and D belong to the genomic islands shown in A and B, respectively. (A) GI_2 (B) GI_4 (see S13 Table in S2 File).

**Fig 10:**
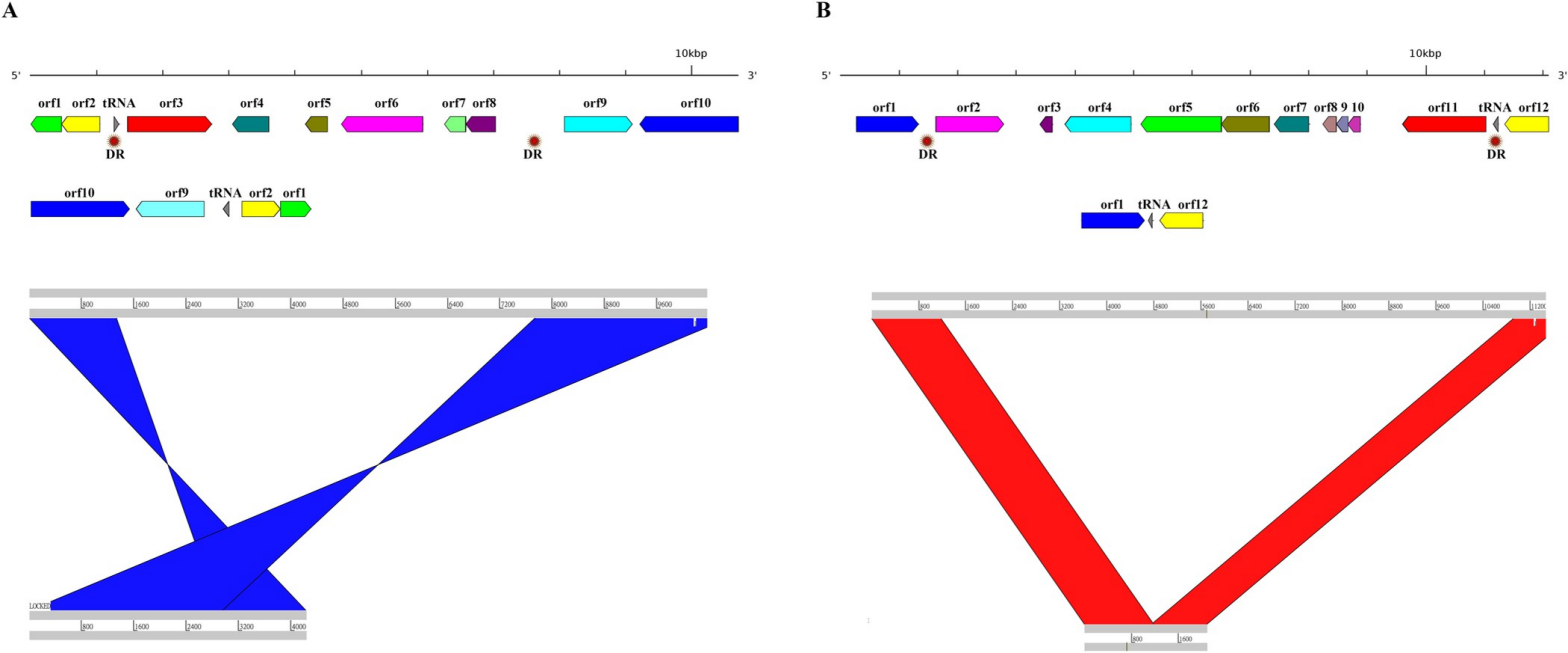
Genomic islands in the genome of *Phaeobacter italicus* discovered in the costal seawater of Badouzi Fishing Port. Gene clusters in the genomic island are shown, and sequence comparisons of the genomic island (upper) with the corresponding genome of *Phaeobacter italicus* available in NCBI (lower) are shown in (A) GI_2 (B) GI_4 (see S13 Table in S2 File). tRNA genes are colored in gray. “DR” (in red circles) indicates the direct repeat on the two flanking sides of the genomic islands.

We assembled microbial genomes of high quality and abundance from the bacteria collected from Badouzi Fishing Port and found one affiliated with Rhodobacteraceae that resolved into the species *Phaeobacter italicus*, which fit the criteria (completeness 98.54%, contamination 0%, S12 Table in S2 File). Interestingly, while we compared the genome sequence of *P*. *italicus* from Baoudozi fishing port with that of *P*. *italicus* in NCBI, we found four genomic islands (GIs) of *P*. *italicus* at Badouzi Fishing Port that were not detected in the publicly available genomes in NCBI. Lower GC% contents than the neighboring regions of the genome, tRNA genes located on one end of the genomic island, and two direct repeats in the border of the genomic islands all mark the features of genomic islands. Within these four genomic islands, we discovered genes encoding phage tyrosine integrase, prophage DNA invertase, DNA gyrase inhibitor, restriction enzyme, antitoxin HigA-1, and many proteins of unknown function (S13 Table in S2 File). Of these four GIs, two can map to a single contig of publicly available genomes of *P*. *italicus* in NCBI (Figs 9 and 10). Within these two genomic islands, all genes but one have the same transcription direction, suggesting an operon structure. The protein-coding genes (e.g. antitoxin HigA-1, metallopeptidase, SNARE associated Golgi protein, Diguanylate cyclase, and CbiX protein for Cobalamin (Vitamin B12) biosynthesis, S13 Table in S2 File) within GIs would confer the advantage for the bacteria to adapt in the environment. This discovery implies that the horizontal transfer of genomic islands could facilitate the adaptation of *P*. *italicus* in the seawater environment of the fishing port.

### The community network structure of the microbiome of the coastal seawater at the fishing port and the nearby island

**Fig 11:**
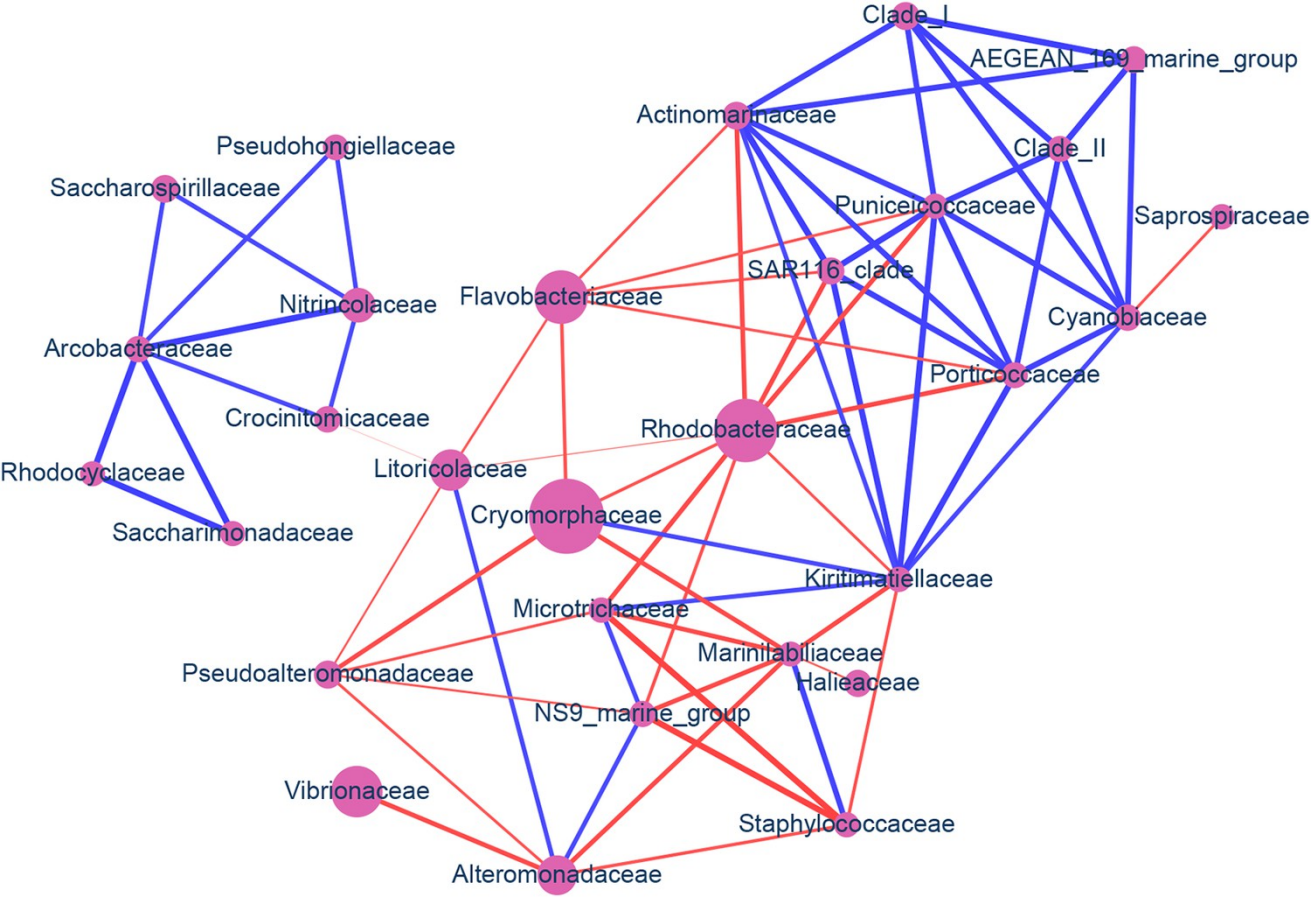
Microbial community network in the costal seawater of Heping Island and Badouzi Fishing Port. The color of the network edges denotes positive (blue) and negative (red) correlation, and the edge width is proportional to the correlation coefficient. Only network edges with an absolute value of the correlation coefficient larger than 0.8 and bootstrap *p* value smaller than 0.01 were retained. Family names of bacteria are designated.

We generated the community network structure by analyzing OTU results from 16S rRNA gene V3V4 amplicon sequencing (Fig 11). We found that the community network was composed of one main cluster associated with a small cluster. The main cluster of the network can be roughly divided into two parts: one with mainly coexistence and the other with mutual exclusion. The network with mainly coexistence was composed of the bacteria AEGEAN_169_marine_group, Clade_I, Clade_II, Cyanobiaceae, Puniceicoccaceae, Porticoccaceae, Actinomarinaceae, SAR116_clade, and Kiritimatiellaceae; the other network with mutual exclusion consisted of Rhodobacteraceae, Cryomorphaceae, Flavobacteriaceae, Litoricolaceae, and Marinilabiliaceae. The small cluster consisted of Pseudohongiellaceae, Nitrincolaceae, Saccharospirillaceae, Crocinitomicaceae, Arcobacteraceae, Rhodocyclaceae, and Saccharimonadaceae, all with coexistence relationships among the constituent nodes while connecting with the main cluster (through Litoricolaceae) by mutual exclusion. Interestingly, Alteromonadaceae formed its coexistence relationship with Litoricolaceae and NS9_marine_group, the latter with Microtrichaceae, then Kiritimatiellaceae, and then Cryomorphaceae. However, Alteromonadaceae maintained its mutual exclusion relationships with Vibrionaceae, Marinilabiliaceae, and Staphylococcaceae.

## Discussion

### The bacterial community structures in coastal seawater from a fishing port and a nearby island were different

We employed next-generation sequencing to decipher the microbiome compositions and infer the biochemical properties of bacteria in seawater at two nearby locations facing the Northwestern Pacifica Ocean and the East China Sea. After investigating the derived biochemical functions from gene-coding protein sequences based on the genome sequences, some features emerged (Fig 11, S14 Table in S2 File). At Heping Island, Alteromonadaceae, Cryomorphaceae, Flavobacteriaceae, Litoricolaceae, and Rhodobacteraceae were dominant. They harbored genes with the functions of denitrification, nitrate assimilation, phytoplankton-derived glucan degradation, phosphate transport, lipopolysaccharide transport, sugar transport, antibiotic resistance (diaminopyrimidines, macrolide), and metal tolerance (copper and multimetal). Conversely, at Badouzi Fishing Port, we found the emergence of much higher proportions of Oceanospirillaceae, Rhodobacteraceae, and Vibrionaceae. More gene sequences with the functions of nitrogen fixation, denitrification, nitrate assimilation, biofilm formation, cell wall remodeling, transport of branched-chain amino acids, transport of phosphate, transport of lipopolysaccharide, flagellar motility, antibiotic resistance (nitroimidazole, aminocoumarin, ansamycin), metal tolerance (multimetal, copper, chromium, iron), chemotaxis and flagella and T3SS1 were discovered.

**Fig 12:**
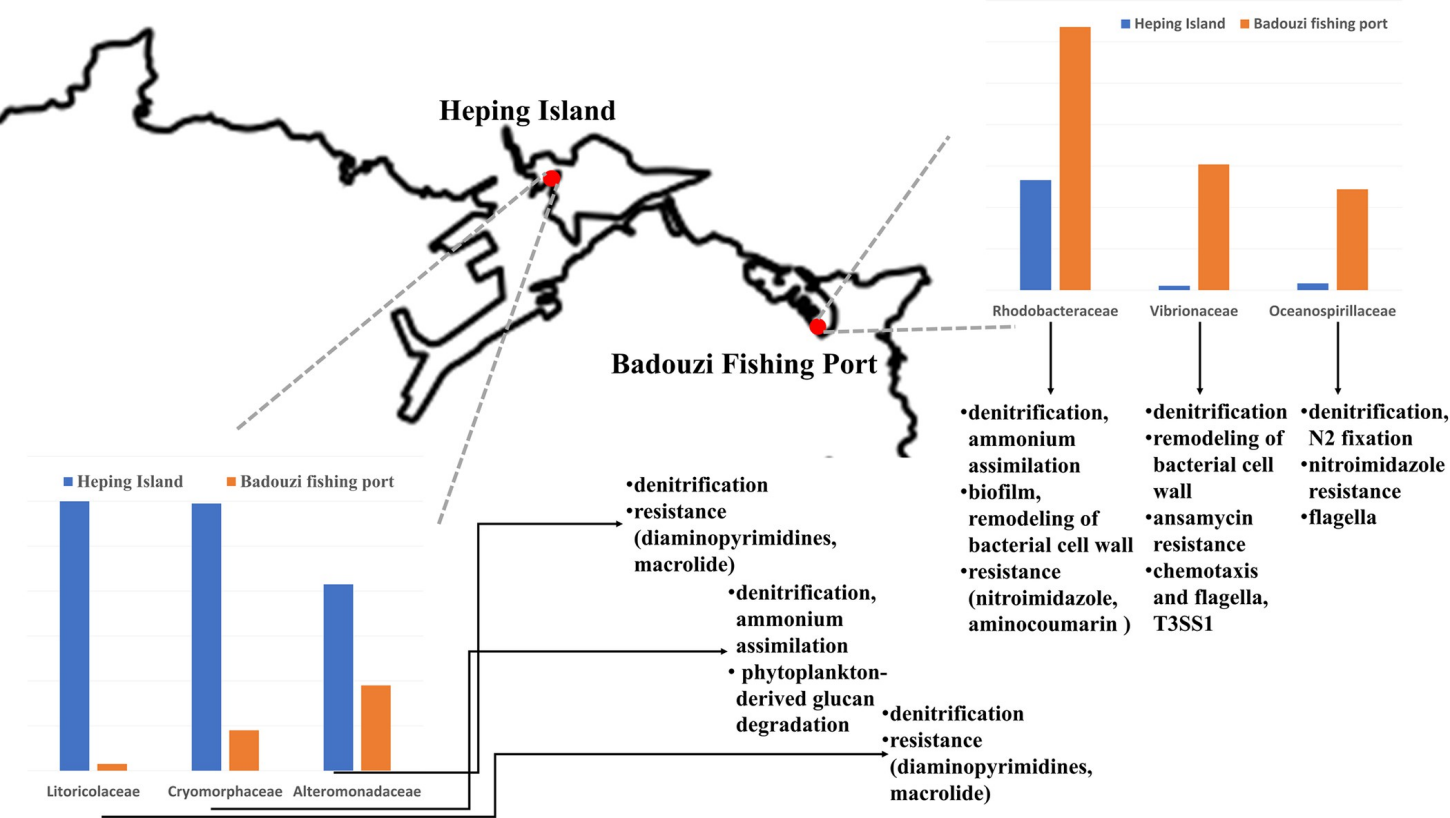
A conceptual figure for major discoveries of the microbiome from the costal seawater of Heping Island and nearby Badouzi Fishing Port. Dominant species in two sampling sites are demonstrated. Specific functions of genes encoding for the nitrogen metabolism, carbohydrate metabolism, antibiotics resistance, and virulence factors are summarized.

Alphaproteobacteria, Gammaproteobacteria, and Flavobacteriia are heterotrophic bacteria and have been discovered to respond to phytoplankton blooms by actively utilizing the nutrients (such as dissolved organic matter) released by phytoplankton [59, 60]. Cryomorphaceae (within Flavobacteriia) can degrade the high molecular weight substrates of coastal phytoplankton and have been found in coastal surface seawater worldwide [61]. Cryomorphaceae was also found to play important roles in the nutrient dynamics in inshore reefs in the Great Barrier Reef [62]. Litoricolaceae (within Gammaproteobacteria) was discovered in surface seawater in the Yellow Sea, Korea [63]. A metabolomic study showed that *Litoricola marina* (in the family Litoricolaceae) feeds on nucleotides and nucleosides in the phytoplankton bloom area [64]. A phycosphere study in the seawater of east-south Asia (Xiamen, Hong Kong, and the South China Sea) also showed that Rhodobacteraceae (within Alphaproteobacteria) was abundantly associated with marine *Synechococcus* at all three sampling locations; however, Alteromonadaceae (within Gammaproteobacteria) was abundant in coastal water around Xiamen and Hong Kong, and Flavobacteriaceae (within Flavobacteriia) were abundant only in the South China Sea [65]. In our study, both 16S rRNA gene amplicon and whole-genome shotgun sequencing results indicated that Alteromonadaceae, Cryomorphaceae, Flavobacteriaceae, Litoricolaceae, and Rhodobacteraceae were the five most abundant families in the seawater of Heping Island. This implies that in July of the sampling time, phytoplankton existed in the seawater of Heping Island.

The community structure inferred from our study suggested that in the seawater of Heping Island, Alteromonadaceae, Litoricolaceae, and Cryomorphaceae formed a coexistence relationship with a group of bacteria, including AEGEAN_169_marine_group, Clade_I, Clade_II, Cyanobiaceae, Puniceicoccaceae, Porticoccaceae, Actinomarinaceae, SAR116_clade, and Kiritimatiellaceae, most of which were reported to be abundant in phytoplankton blooms [66, 67]. Conversely, Rhodobacteraceae and Vibrionaceae showed higher abundances at Badouzi Fishing Port and formed mutual-exclusion relationships with the abovementioned bacteria, which maintained a relationship of coexistence. Moreover, the isolated group comprising Pseudohongiellaceae, Nitrincolaceae, Saccharospirillaceae, Crocinitomicaceae, Arcobacteraceae, Rhodocyclaceae, and Saccharimonadaceae formed mutual-exclusion relationships with Litoricolaceae and showed a higher abundance at Badouzi Fishing Port than at Heping Island. Arcobacteraceae was associated with human feces in sewage [68] and was also found in sewer sediment [69]. Rhodocyclaceae was demonstrated to function in single-chamber microbial fuel cells as a denitrifier [70]. Ethylbenzene dehydrogenase, which conducts the anaerobic bacterial degradation of ethylbenzene and propylbenzene, was also from Rhodocyclaceae [71]. Saccharimonadaceae was found to be more abundant in colorectal cancer (CRC) model mice when chromium was administered [72]. Saccharimonadaceae is also an intestinal metabolizer and microbial degrader in earthworms [73, 74].

### Potential impacts posed by the bacterial community in the coastal seawater of fishing ports

Rhodobacteraceae was shown to be able to utilize a different form of carbon source [75] and to have large amounts of ABC transporters [65]. Rhodobacteraceae was also the most abundant bacterium involved in biofilm formation in eastern Mediterranean coastal seawater [76] and the western Pacific Ocean [77]. Rhodobacteraceae was the abundant taxon in the coastal waters of the reefs, inlets, and wastewater outfalls of southeast Florida, USA [78], where Rhodobacteraceae showed significant correlations with oxygen level, salinity, pH, and nitrate concentration. In the southern coastal seawater of India, both Rhodobacteraceae and Vibrionaceae were involved in biofilm formation [79]. Investigation of the microbial community in the Persian Gulf also showed that after chronic exposure and oil spill events, Oceanospirillales (containing Oceanospirillaceae) was enriched in the high aliphatic content of the pollution, and Rhodobacterales (containing Rhodobacteraceae) was enriched in polyaromatic pollution, leading to the hypothesis of the division of labor for bioremediation [80]. Climate, environment, and human activity have been shown to correlate with the abundance of the *Vibrio* genus [81, 82]. The spatiotemporal distribution and abundance of *Vibrio* [83] in the marine aquaculture environment of Dongshan Bay in the southwest Taiwan Strait were investigated, and 28 species were found to vary in abundance between seasons. Vibrionaceae was reported to be associated with cage-cultured marine fishes, plants, algae, zooplankton, and animals (fish, oysters, etc.) [84, 85]. Vibrionaceae contains many pathogens and is found in the aquatic environment [86]. At Badouzi Fishing Port, where leaked oil and dead fish body pollution pose serious problems in the sea environment, we observed the abundant presence of Rhodobacteraceae, Vibrionaceae, and Oceanospirillaceae, with genes conferring biochemical functions that lead to adaptation to the environment and allow them to thrive. More virulence genes also appeared at Badouzi Fishing Port, which poses public health problems.

Antibiotic resistance seen in the microbiome poses a serious threat to the environment and humans. A recent study using shotgun metagenomic sequencing of the intestinal microbiome of deep-sea fish from the Atlantic Ocean revealed that fish in the deep sea almost do not have antibiotic resistance genes [87]. In contrast, metagenomic samples from the TARA Oceans project revealed a wide distribution of antibiotic resistance genes in microbes in the ocean [88]. A study in the coastal area of northwestern Sicily, Italy, showed that seawater with polyethylene pollution led to the accumulation of antibiotic resistance genes in the microbiome [89]. Another study in collected fish from the mainland and marine environments in China as well as from Chile and Nigeria showed that antibiotic resistance in marine fish shared high similarity and high abundance and was distinct from that observed in terrestrial animals [90]. In this study, we found more antibiotic resistance genes (nitroimidazole, aminocoumarin, and ansamycin) in the microbiome in the seawater of the Badouzi Fishing Port. The pattern of antibiotic resistance at Badouzi Fishing Port was different from that at Heping Island. This result suggested that the pollution at Badouzi Fishing Port posed an additional potential threat to the community in the fishing port. Metal tolerance has recently gained more attention due to the impact of metal pollution on microbial communities and ecological functions [91]. We also identified abundant metal tolerance genes at Badouzi Fishing Port. For example, *Vibrio* contains several multimetal-tolerant genes, of which the mechanisms of tolerance include channel (glpF), efflux (corC), enzyme (corB, dsbB, modB, modC, recG, ruvB), and membrane transporter (fecE, mntH/yfeP). Moreover, genes that render chromium resistance were apparent in Vibrionaceae collected in the sea of the Badouzi Fishing Port. Tolerance to chromium by *Vibrio parahaemolyticus* in China was reported before [92], and the tolerance concentration of *Vibrio* can be as high as 3200 μg/mL [93]. Antimicrobial resistance can be propagated through the food chain from antimicrobial-resistant bacteria to the contaminated food consumed by animals and humans [94]. Even the fishmeal containing antimicrobial-resistant genes were detected in mariculture sediment, posing another round of antimicrobial resistance propagation [95].

We found an increase in genes affiliated with denitrification and N_2_ fixation in the microbiome in seawater of the Badouzi Fishing Port. This result suggested that the input of a nitrogen source provided some advantage in the environment to certain bacteria. A laboratory study showed that abrupt increases in nitrate or ammonium in surface water from the central North Pacific Ocean stimulated the growth of Oceanospirillaceae and Rhodobacteraceae in incubations in the dark [96]. Interestingly, a study on the biocrust showed that one of the most abundant transcriptionally active N-transforming microorganisms in the investigated biocrusts was affiliated with Rhodobacteraceae [97].

Scrutiny of the contigs from the collection of whole-genome shotgun sequencing revealed abundant genes coding for the T3SS1 apparatus at the Badouzi Fishing Port. A protein sequence similarity search against the NCBI database indicated that four T3SS1s belonged to *Vibrio harveyi* (three contigs), *Vibrio alginolyticus* (one contig), and *Vibrio diabolicus* (one contig). Recently, a study on the whole genomes of 110 *Vibrio* species from coastal areas in China demonstrated that T3SS1 genes were universally detected in *Vibrio parahaemolyticus*, *Vibrio alginolyticus*, *Vibrio harveyi*, and *Vibrio campbellii* [98]. Our effort in binning the reads from whole-genome shotgun sequencing yielded a *Vibrio* sequence of sufficient completeness to the genus level. We are currently employing long-read sequencing technology (such as Oxford Nanopore sequencing or PacBio single-molecule real-time sequencing) [99–101], aiming to assemble a complete metagenome-assembled bacterial genome and reach a thorough investigation of whole TSS1 clusters.

Through investigating the community network structure (Fig 11), both coexistence and mutual-exclusion networks were discovered. Dominated bacteria in the seawater of Heping Island were mainly composed of a coexistence network; however, the dominated bacteria in the seawater of Badouzi Fishing Port posed an “antagonistic” relation with the coexistence network, forming mutual-exclusion interaction. Ecological niche overlap and resource competition [102–104] were demonstrated for causing the community structure change. In our study, we suggest that the dominant bacteria in the seawater of Heping island shared resources and, possibly, formed a metabolite exchange scenario in the natural ecological environment; nonetheless, in the seawater of Badouzi Fishing Port, the pollution and human interference caused large scale change of ecological environment, leading to the prospering opportunist bacteria without the need to cooperate with other bacteria.

### Genomic islands as the unit of horizontal transfer among microbes in the seawater of fishing port

Genomic islands could serve as a means for horizontal transfer between bacteria, facilitating their adaptation and evolution [105, 106]. We found candidate genomic islands which have features such as lower GC% contents, tRNA genes located on one end of the genomic island, and two direct repeats in the border of the genomic islands. We also found genes encoding phage integrase and recombinase that could trigger the excision and insertion of genomic elements in an appropriate environment. Genomic islands could harbor pathogenesis genes of medical importance. We found several genomic islands in the assembled genomes from whole-genome shotgun sequencing, many of which contained genes of high interest. For example, in a genomic island of *P*. *italicus* (in the family Rhodobacteraceae), we found the antitoxin HigA-1. HigA-1 belongs to the HigBA system, a toxin-antitoxin system of type II toxin-antitoxin [107–109]. HigB protein can endonucleotically cleave RNA in the ribosome 30S-RNA complex [110, 111]. The HigBA toxin-antitoxin system was suggested to play an important role in stress conditions and its adaptation to environmental challenges. HigA can bind HigB to inactivate its molecular function by binding the promoter of the *higBA* operon, which contains two palindromes [112, 113], requiring three water molecule-mediated amino acid-nucleotide interactions [112]. In our study, the presence of HigA in a genomic island could stem from horizontal transfer and be essential for the survival of *P*. *italicus* in the fishing port environment.

## Conclusions

We discovered, in the northwestern Pacific Ocean, the dominant bacteria Oceanospirillaceae, Rhodobacteraceae, and Vibrionaceae at a fishing port and Alteromonadaceae, Cryomorphaceae, Flavobacteriaceae, Litoricolaceae, and Rhodobacteraceae at a nearby offshore island. Bacteria present at fishing ports develop multiple strategies to adapt to the heavily human-interfered environment, while bacteria at offshore islands are related to phytoplankton. Community structure reflected the change in consortia of bacteria and their relative relationships of both coexistence and mutual exclusion in the sea environment. Genomic islands deduced from the bacterial genomes of the fishing port indicate that horizontal transfer could take place in the seawater of the fishing port, and, using gene exchange, the challenge to and adaptation of bacteria in the polluted sea environment could pose threat to humans and associated activities.

## Supporting information

S1 File (DOCX)

S2 File (XLSX)
