# Supplementary material for: Distinctive microbial community and genome structure in coastal seawater from a human-made port and nearby offshore island in northern Taiwan facing the Northwestern Pacific Ocean
Source: PLoS One. 2023 Jun 9;18(6):e0284022. doi: 10.1371/journal.pone.0284022 (PMC10256201; doi:10.1371/journal.pone.0284022)
Supplement: S1 File — (DOCX) [file pone.0284022.s001.docx]

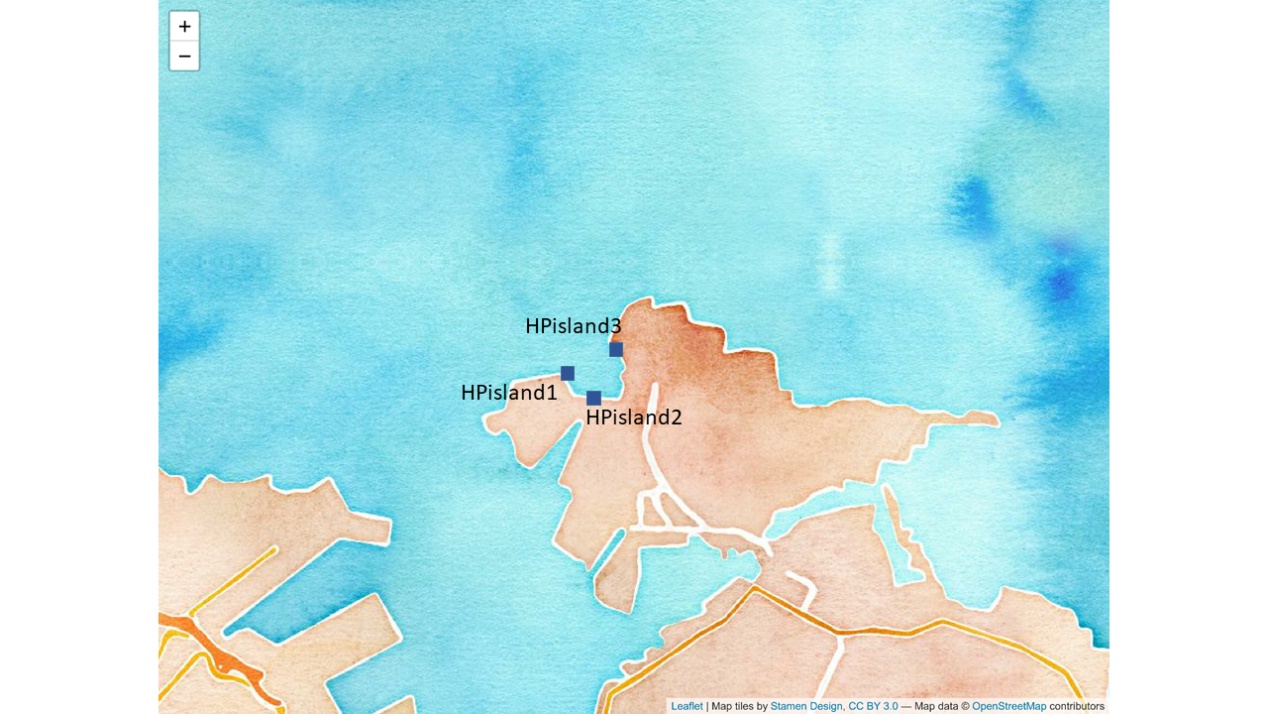


S1 Fig. The map shows three sampling sites in Helping Island (25.161944 N, 121.7625E ) (map was made from open source https://github.com/Wesely/Taiwan-Python-Map).


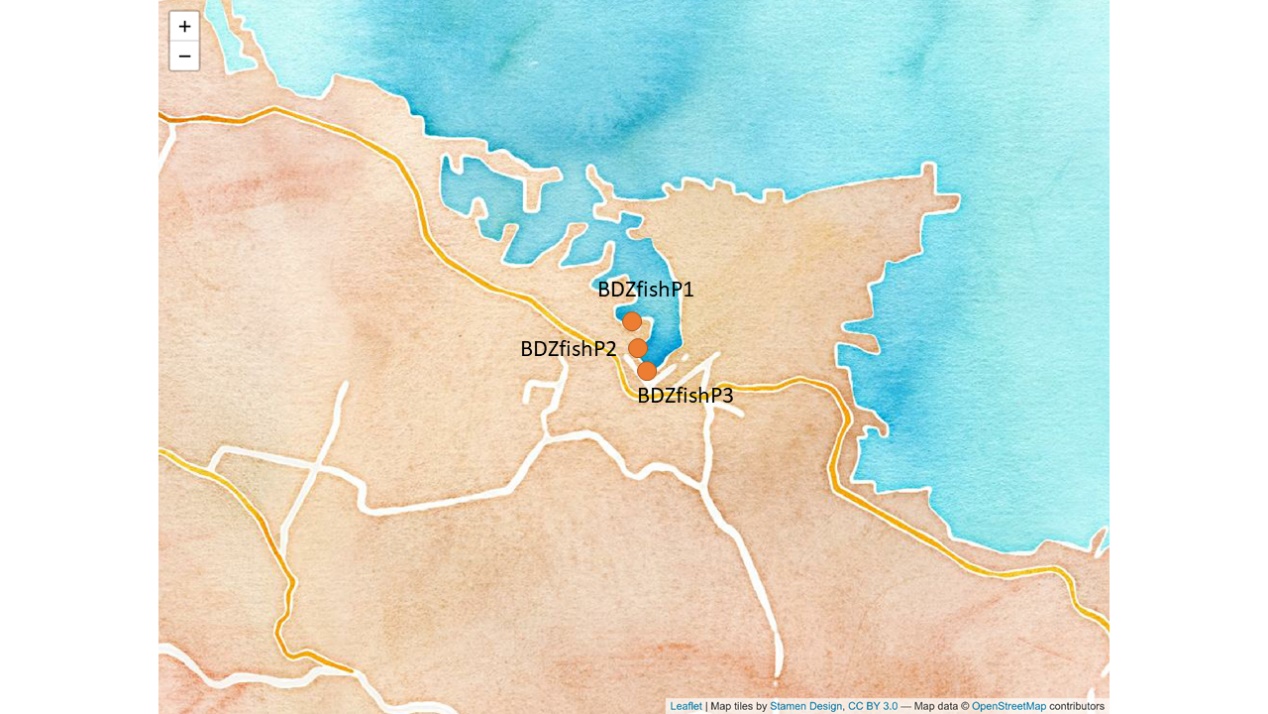


S2 Fig. The map shows three sampling sites in Badouzi fishing port (25.141389N, 121.792778E) (map was made from open source https://github.com/Wesely/Taiwan-Python-Map).


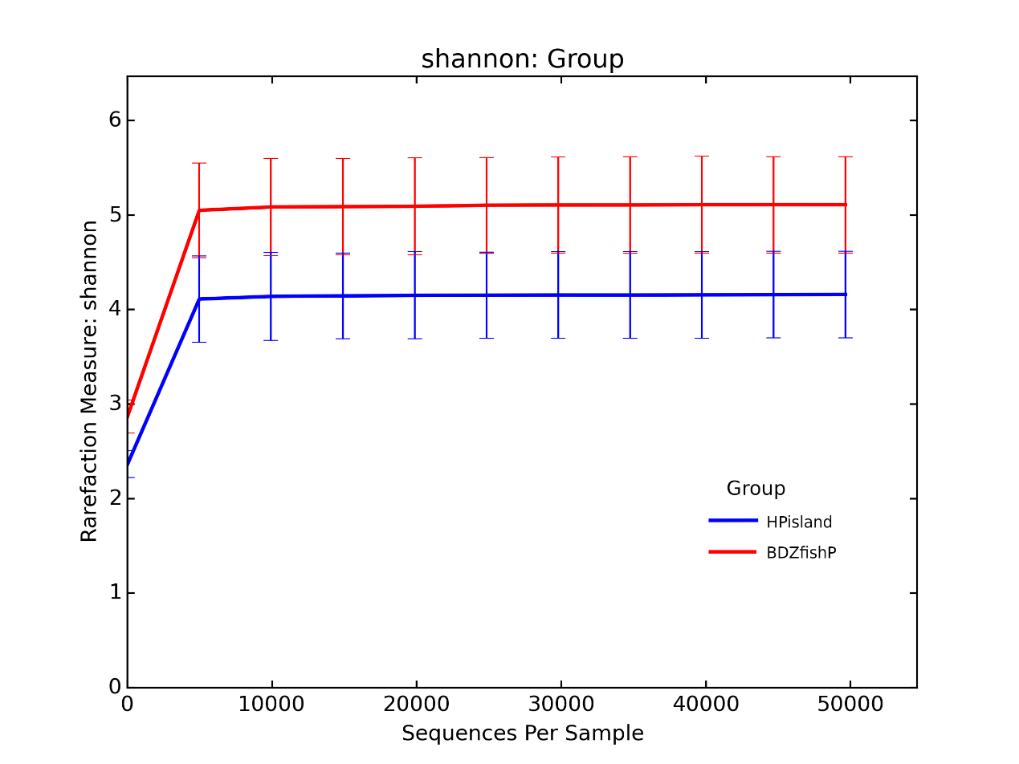


S3 Fig. Rarefaction curve for Shannon index.


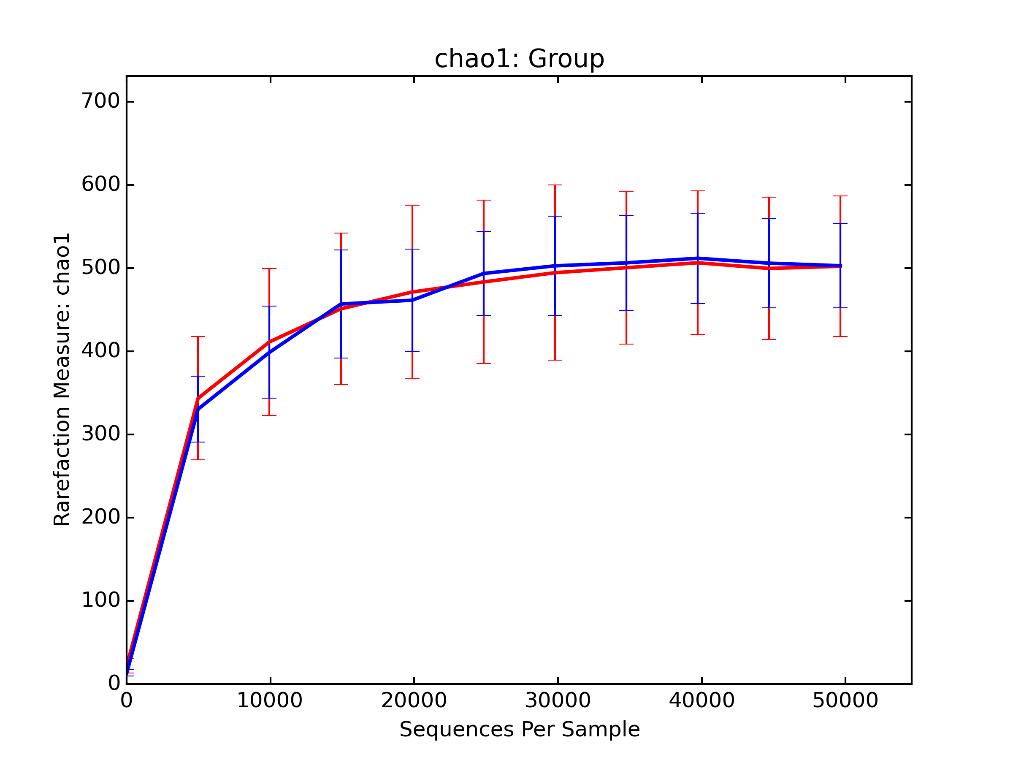


S4 Fig. Rarefaction curve for chao1.


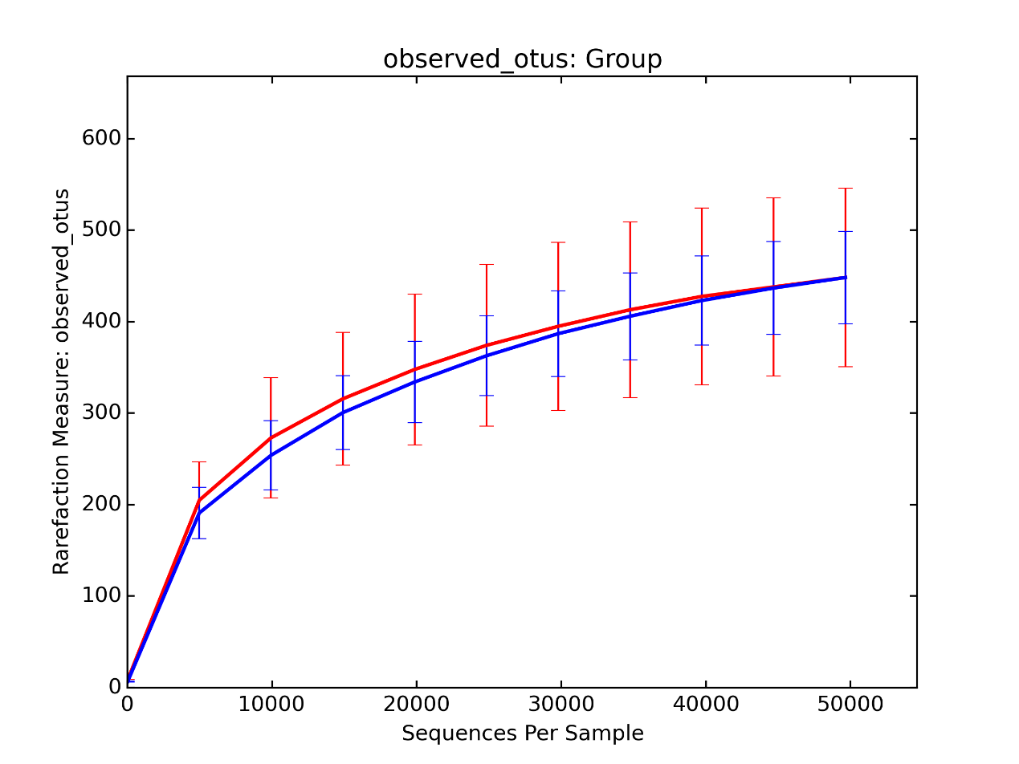


S5 Fig. Rarefaction curve for the observed OTUs.


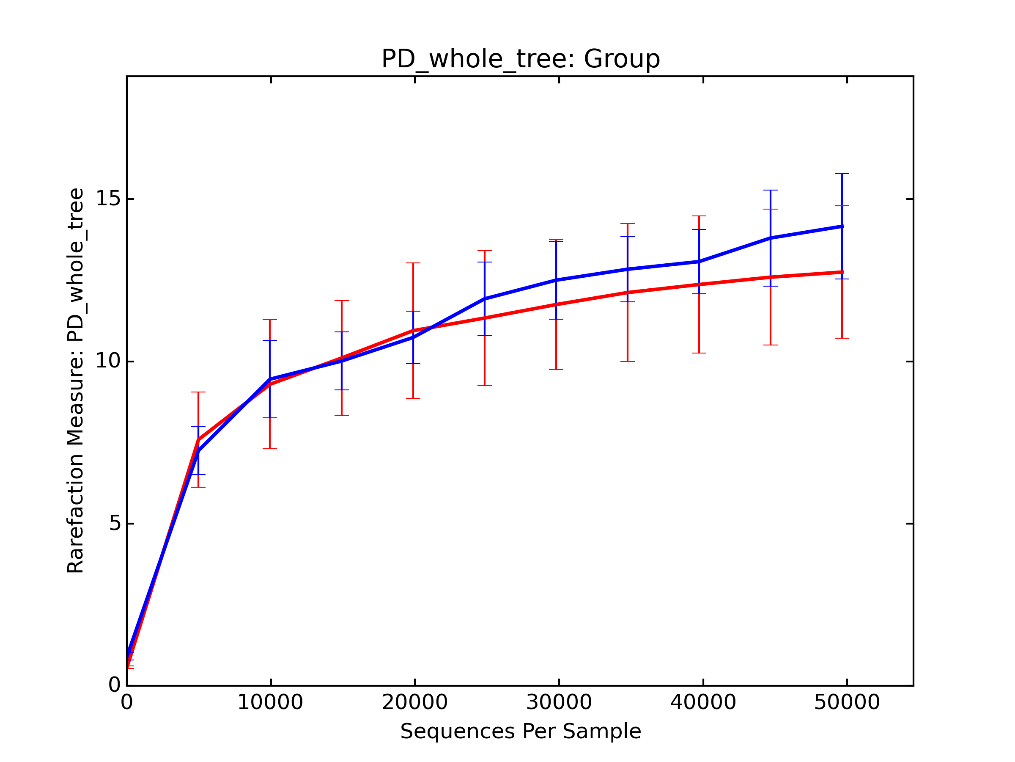


S6 Fig. Rarefaction curve for the phylogenetic distance (PD_whole_tree).


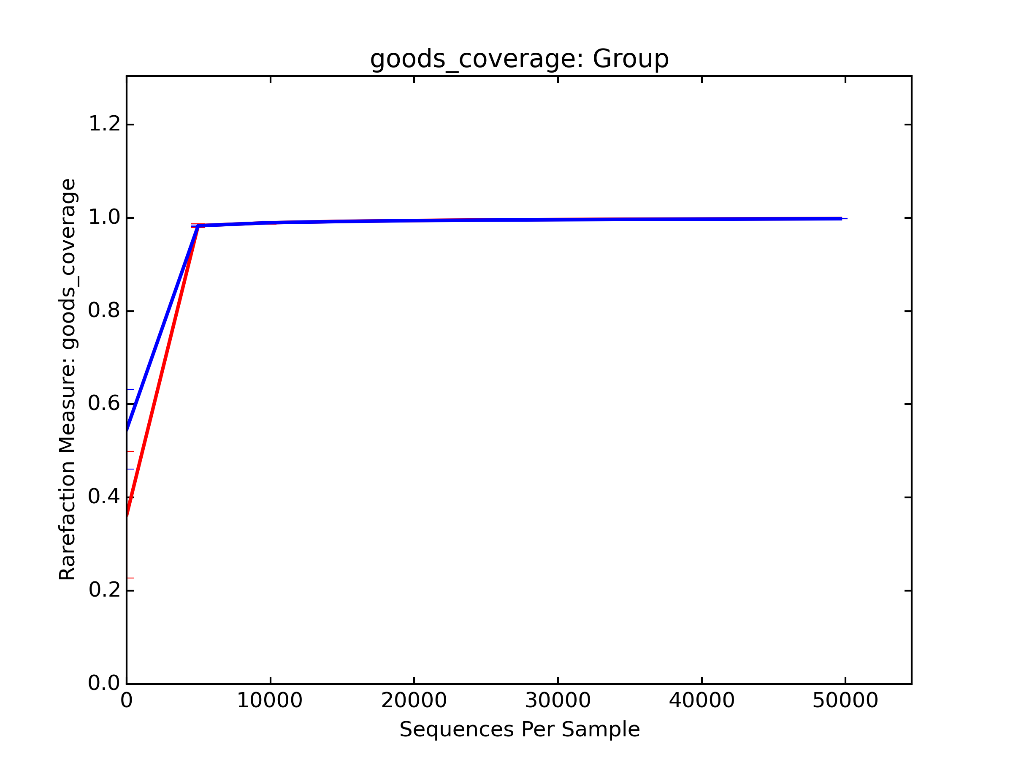


S7 Fig. Rarefaction curve for the goods coverage.


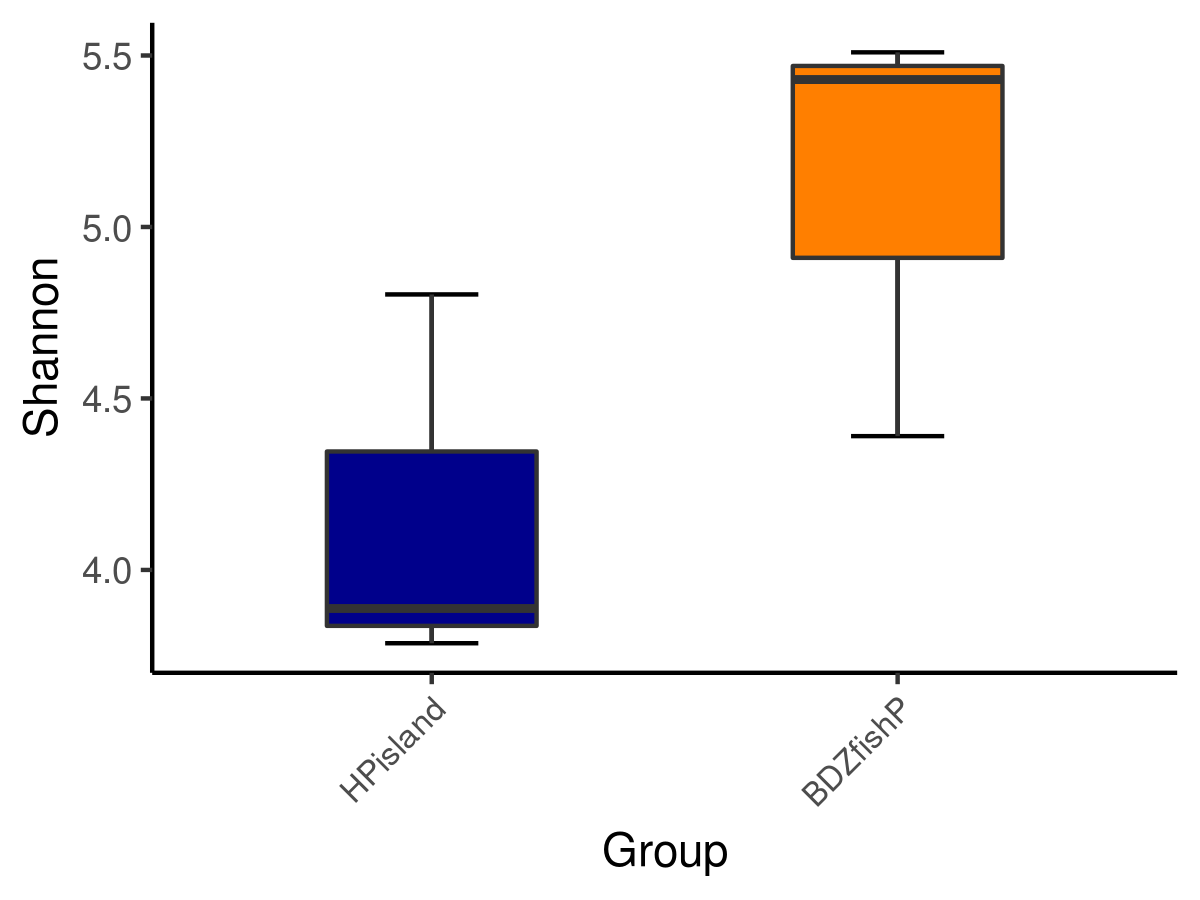


S8 Fig. Boxplot for Shannon index.

.


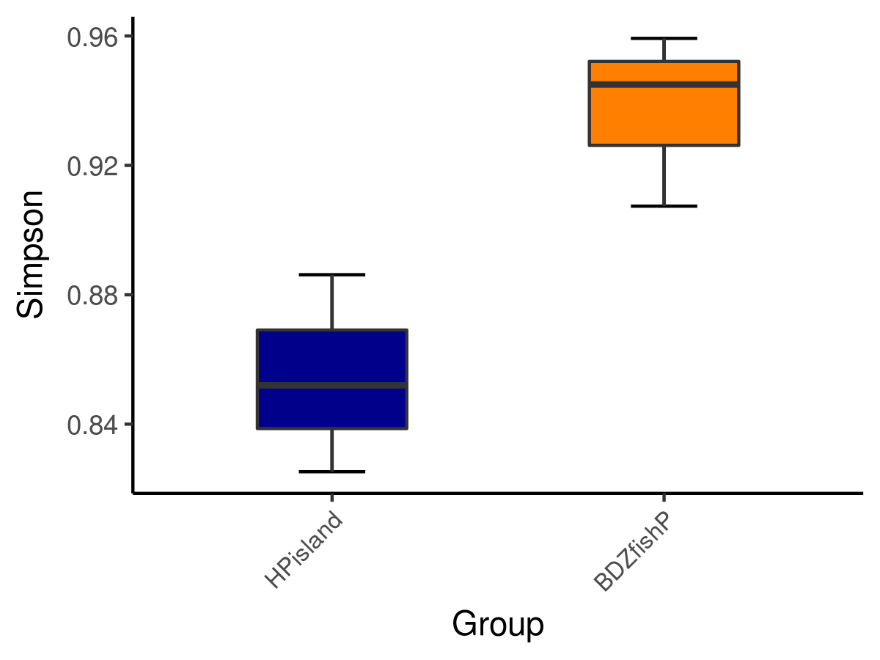


S9 Fig. Boxplot for Simpson index.


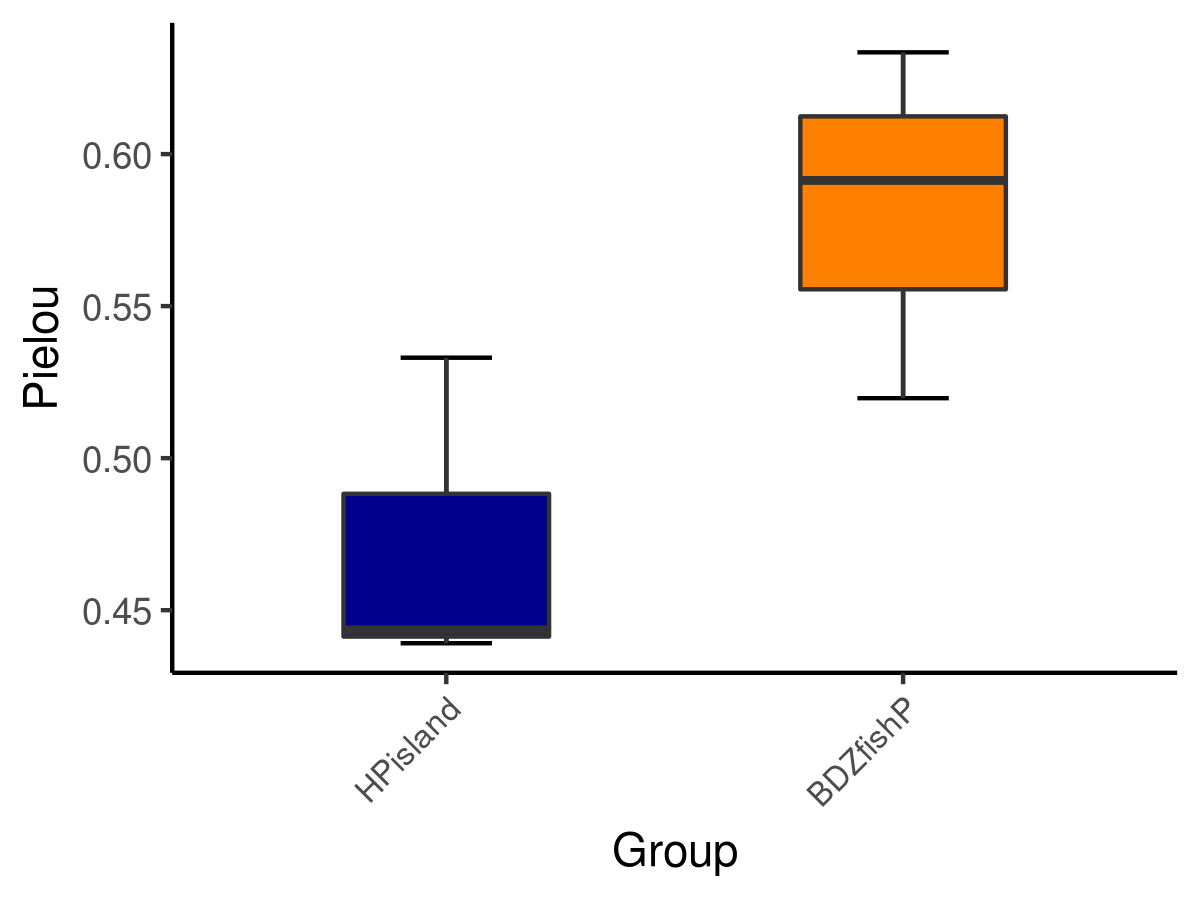


S10 Fig. Boxplot for Pielou's evenness index.


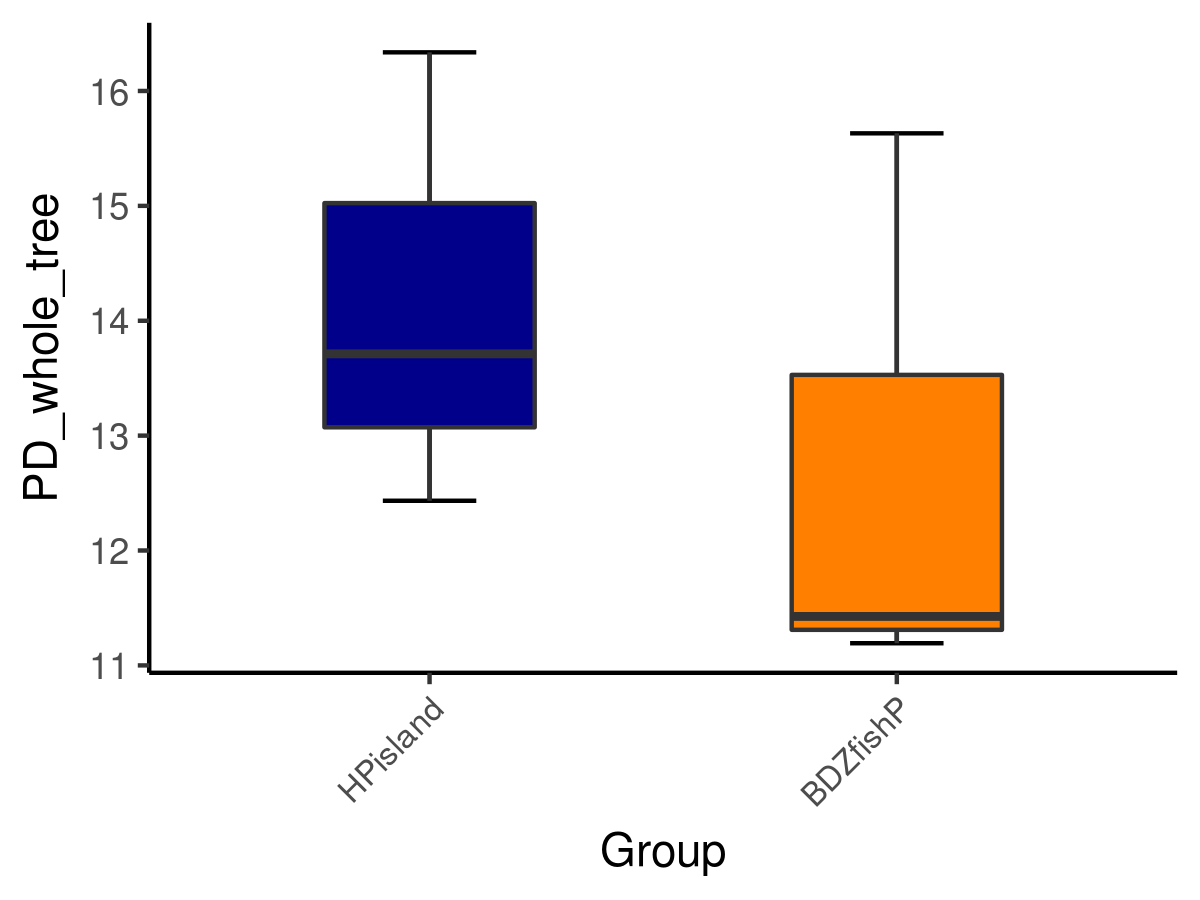


S11 Fig. Boxplot for the phylogenetic diversity (PD whole tree)


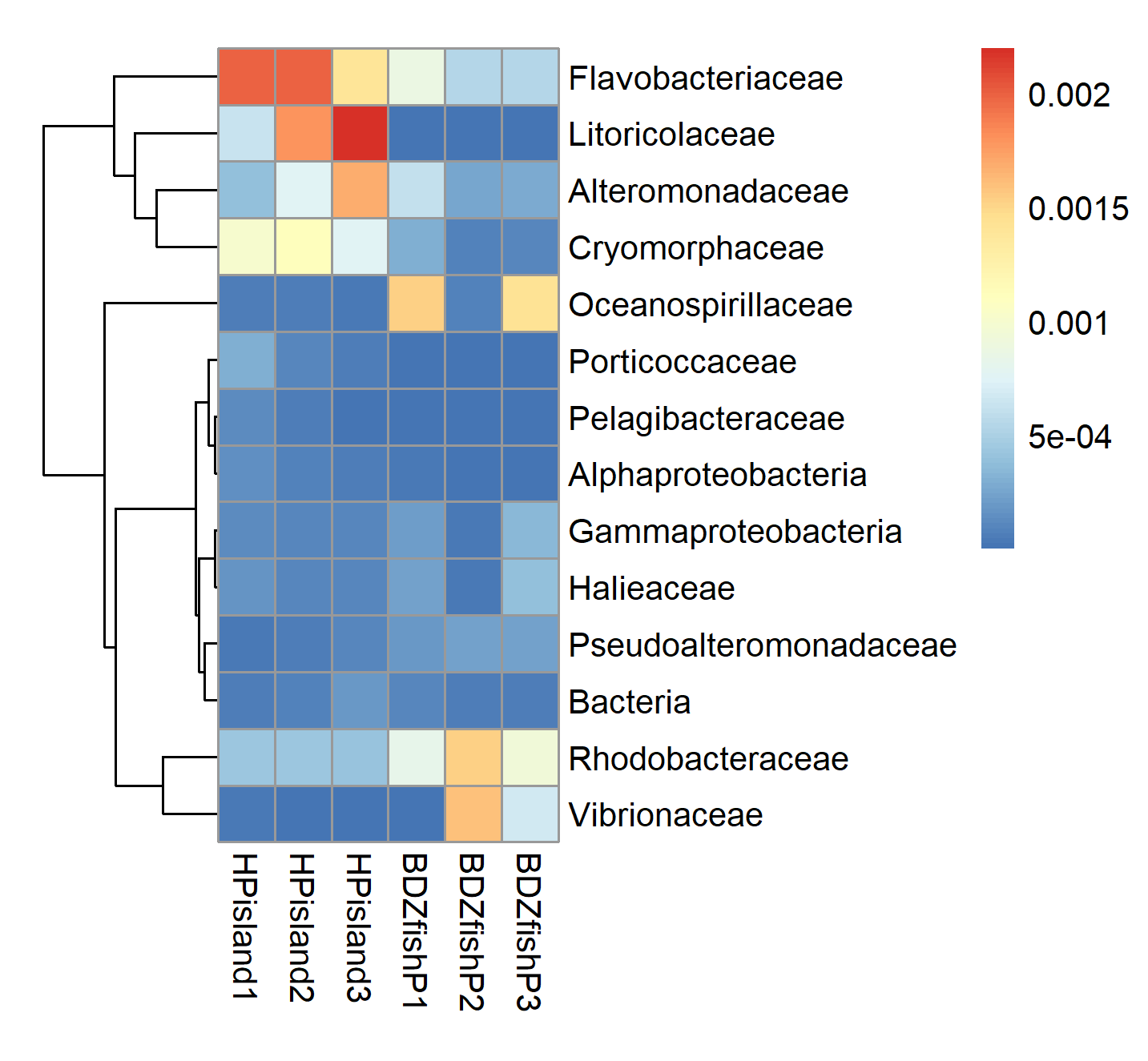


S12 Fig. Taxonomic composition of the *nirK* gene (involved in nitrogen cycling metabolism) in the bacterial communities in Badouzi fishing port (BDZfishP1, BDZfishP2, BDZfishP3) and Heping Island (HPisland1, HPisland2, HPisland3).


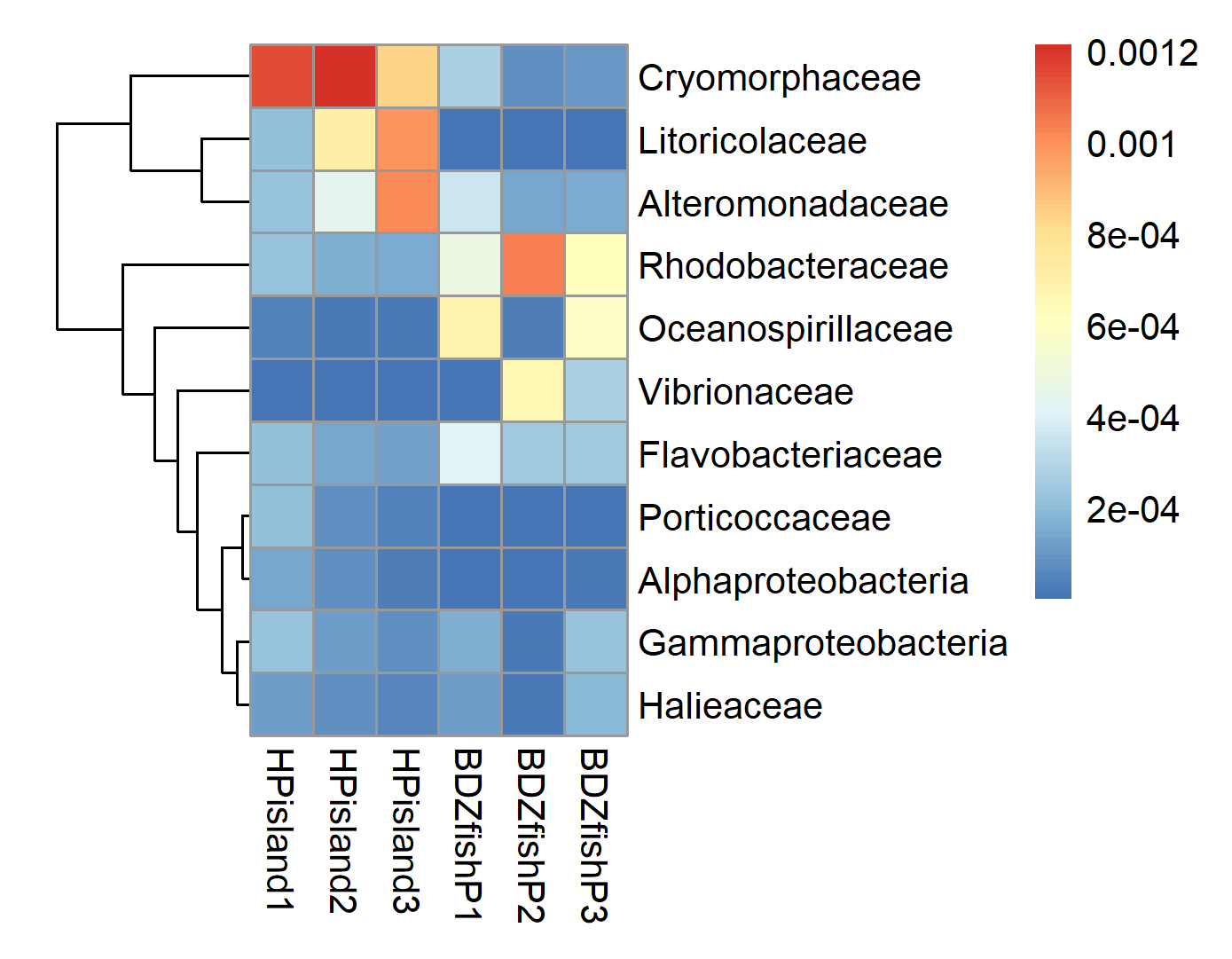


S13 Fig. Taxonomic composition of the *nirS* gene (involved in nitrogen cycling metabolism) in the bacterial communities in Badouzi fishing port (BDZfishP1, BDZfishP2, BDZfishP3) and Heping Island (HPisland1, HPisland2, HPisland3).


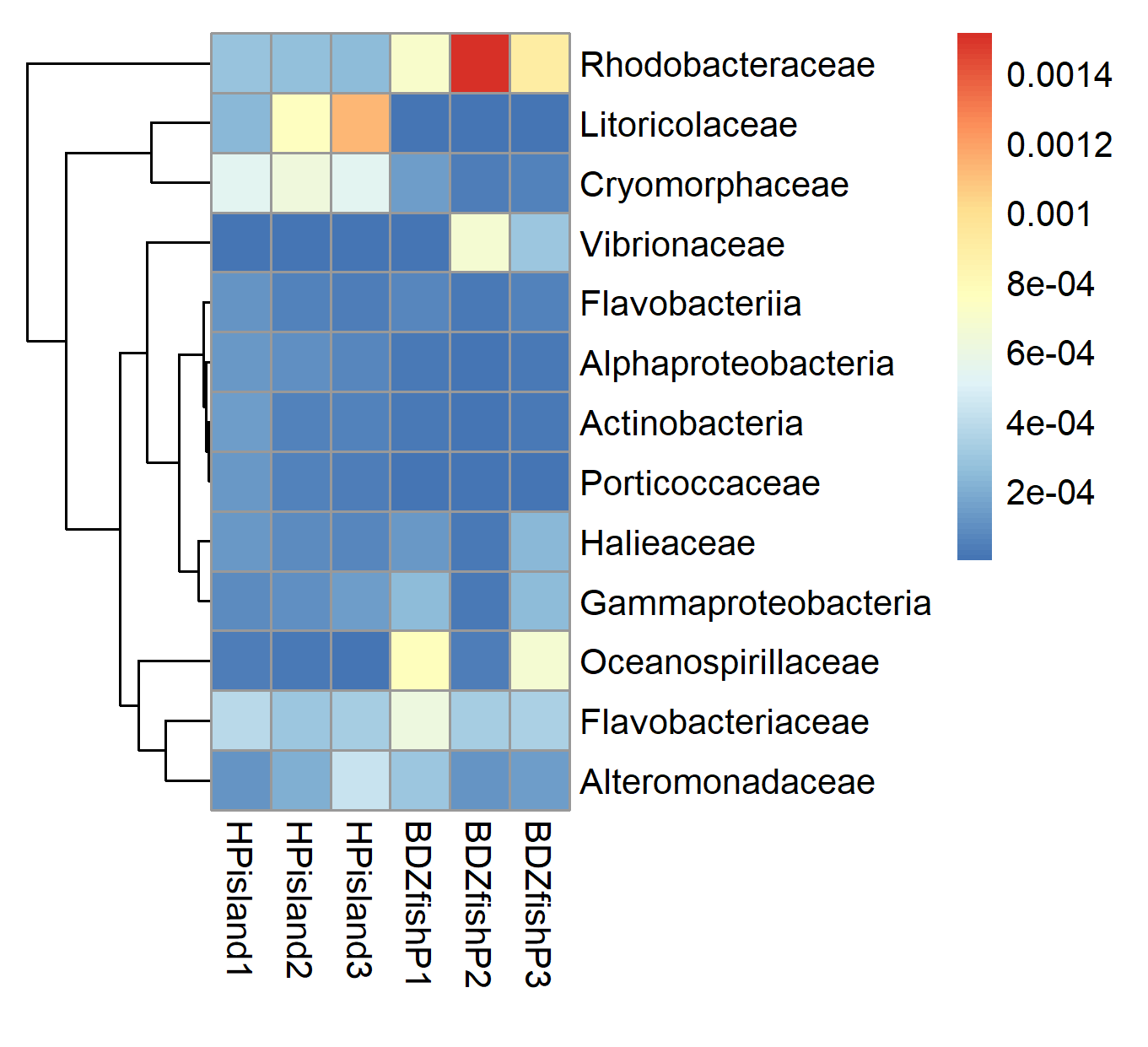


S14 Fig. Taxonomic composition of the *nosZ* gene (involved in nitrogen cycling metabolism) in the bacterial communities in Badouzi fishing port (BDZfishP1, BDZfishP2, BDZfishP3) and Heping Island (HPisland1, HPisland2, HPisland3).


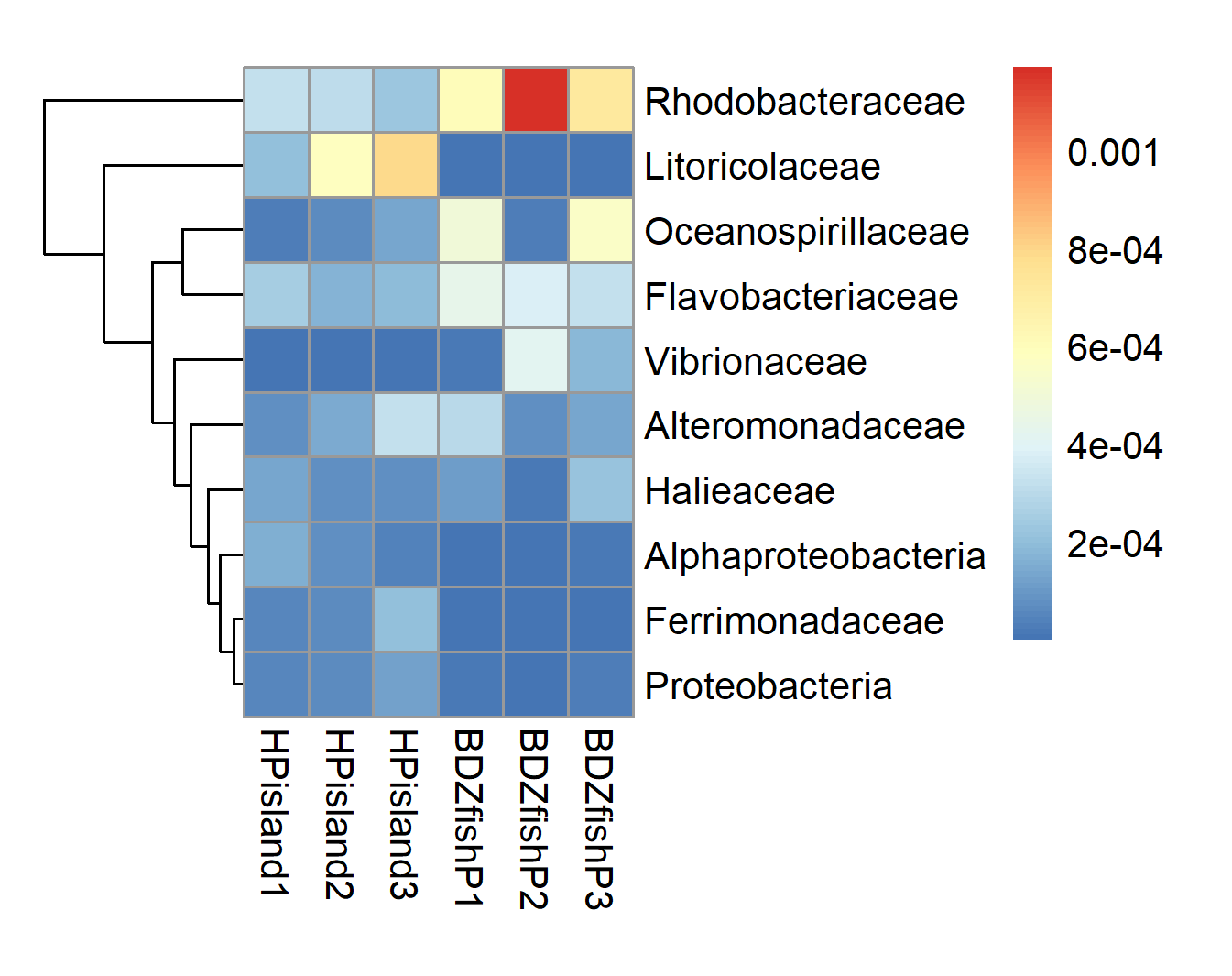


S15 Fig. Taxonomic composition of the *nmo* gene (involved in nitrogen cycling metabolism) in the bacterial communities in Badouzi fishing port (BDZfishP1, BDZfishP2, BDZfishP3) and Heping Island (HPisland1, HPisland2, HPisland3).


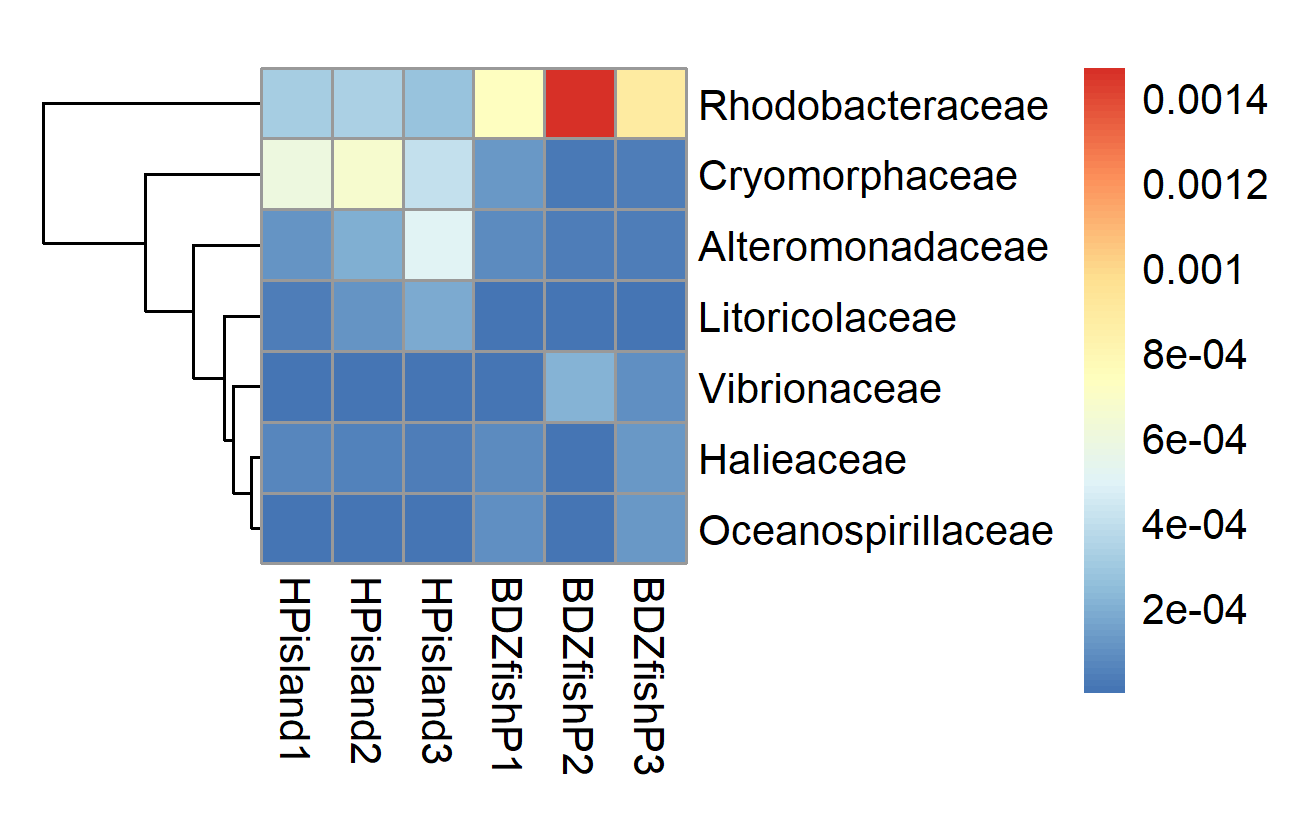


S16 Fig. Taxonomic composition of the *glnA* gene (involved in nitrogen cycling metabolism) in the bacterial communities in Badouzi fishing port (BDZfishP1, BDZfishP2, BDZfishP3) and Heping Island (HPisland1, HPisland2, HPisland3).


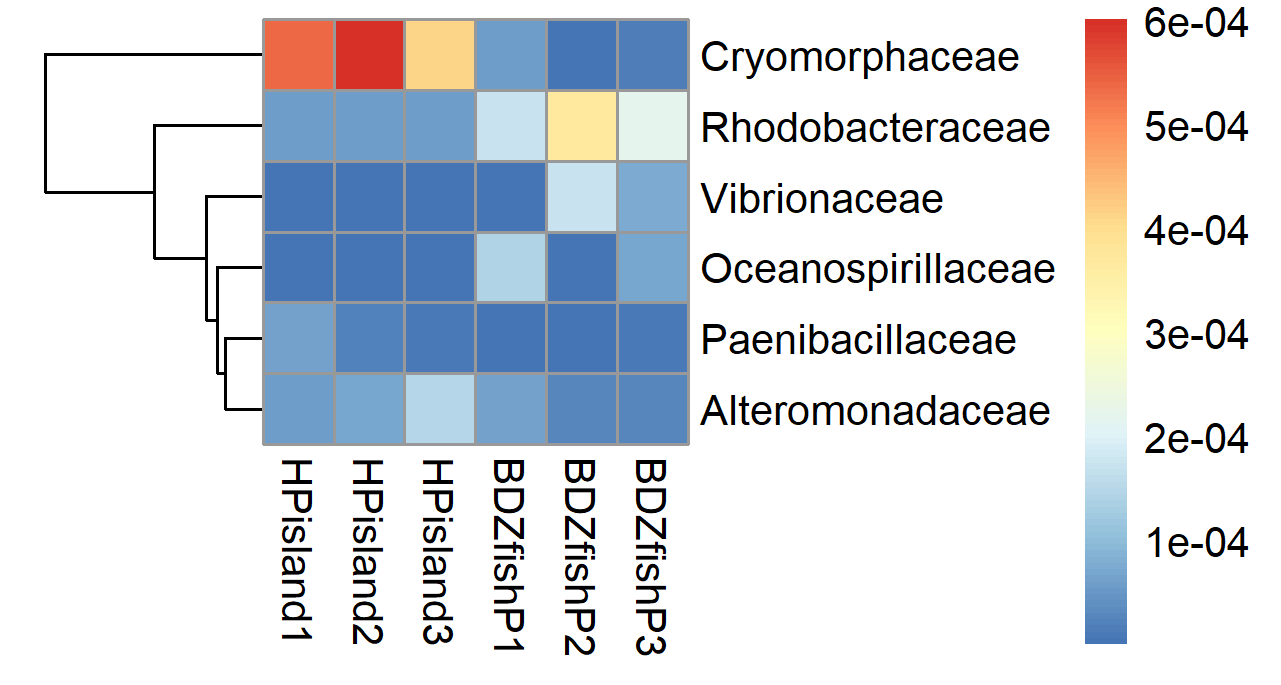


S17 Fig. Taxonomic composition of the *gdh*_*K00261* gene (involved in nitrogen cycling metabolism) in the bacterial communities in Badouzi fishing port (BDZfishP1, BDZfishP2, BDZfishP3) and Heping Island (HPisland1, HPisland2, HPisland3).


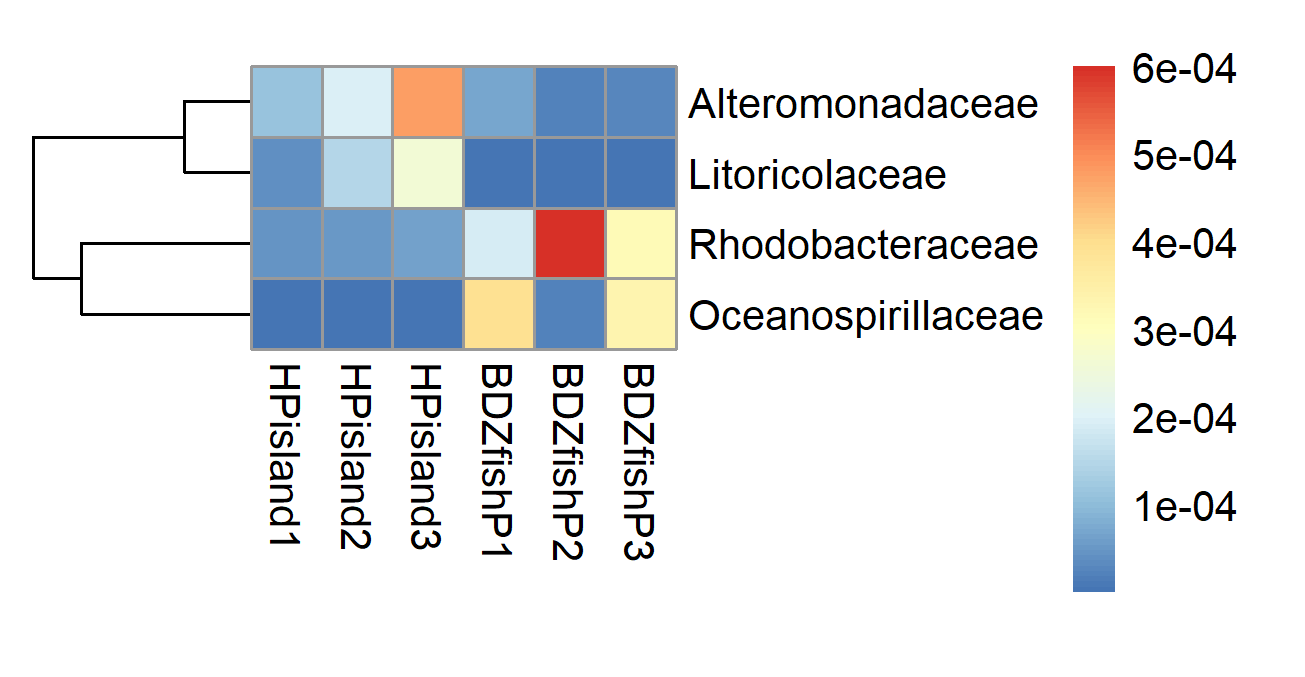


S18 Fig. Taxonomic composition of the *gdh*_*K15371* gene (involved in nitrogen cycling metabolism) in the bacterial communities in Badouzi fishing port (BDZfishP1, BDZfishP2, BDZfishP3) and Heping Island (HPisland1, HPisland2, HPisland3).


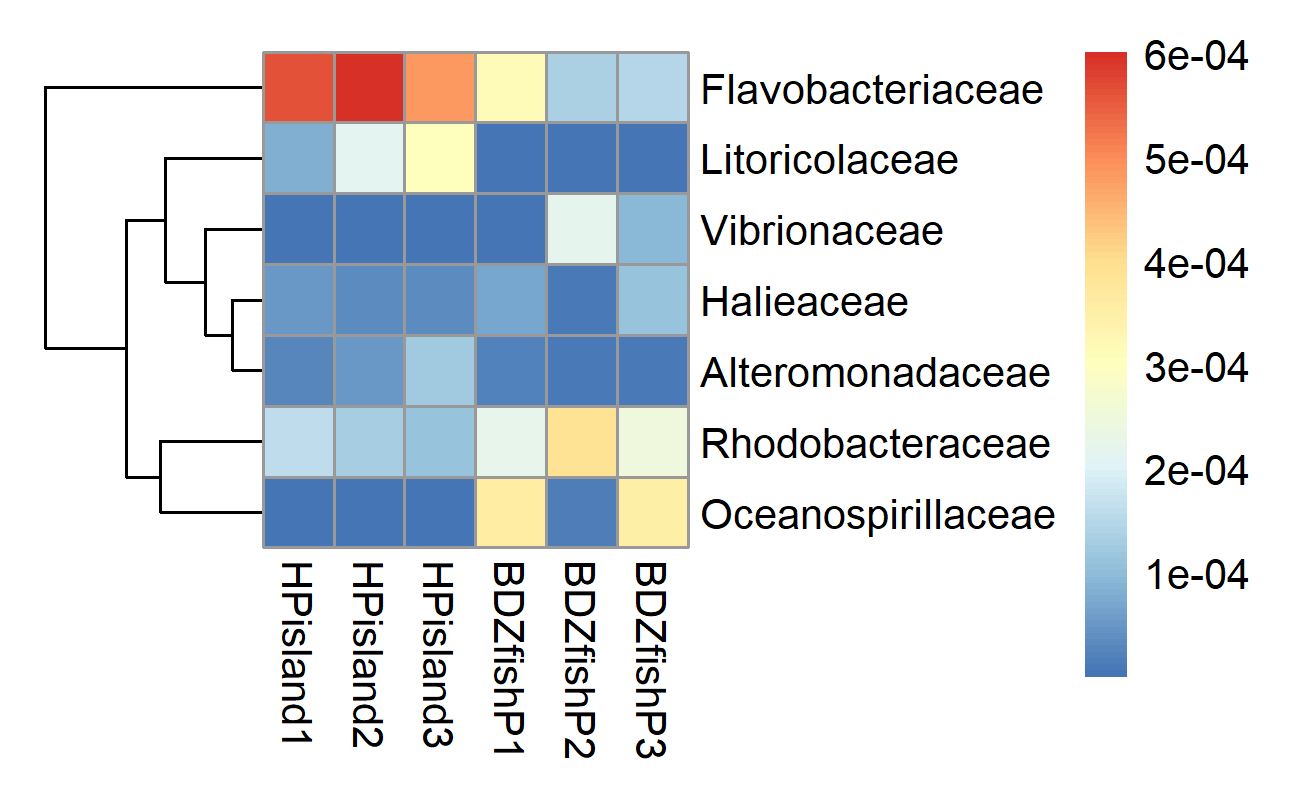


S19 Fig. Taxonomic composition of the *nifH* gene (involved in nitrogen cycling metabolism) in the bacterial communities in Badouzi fishing port (BDZfishP1, BDZfishP2, BDZfishP3) and Heping Island (HPisland1, HPisland2, HPisland3).


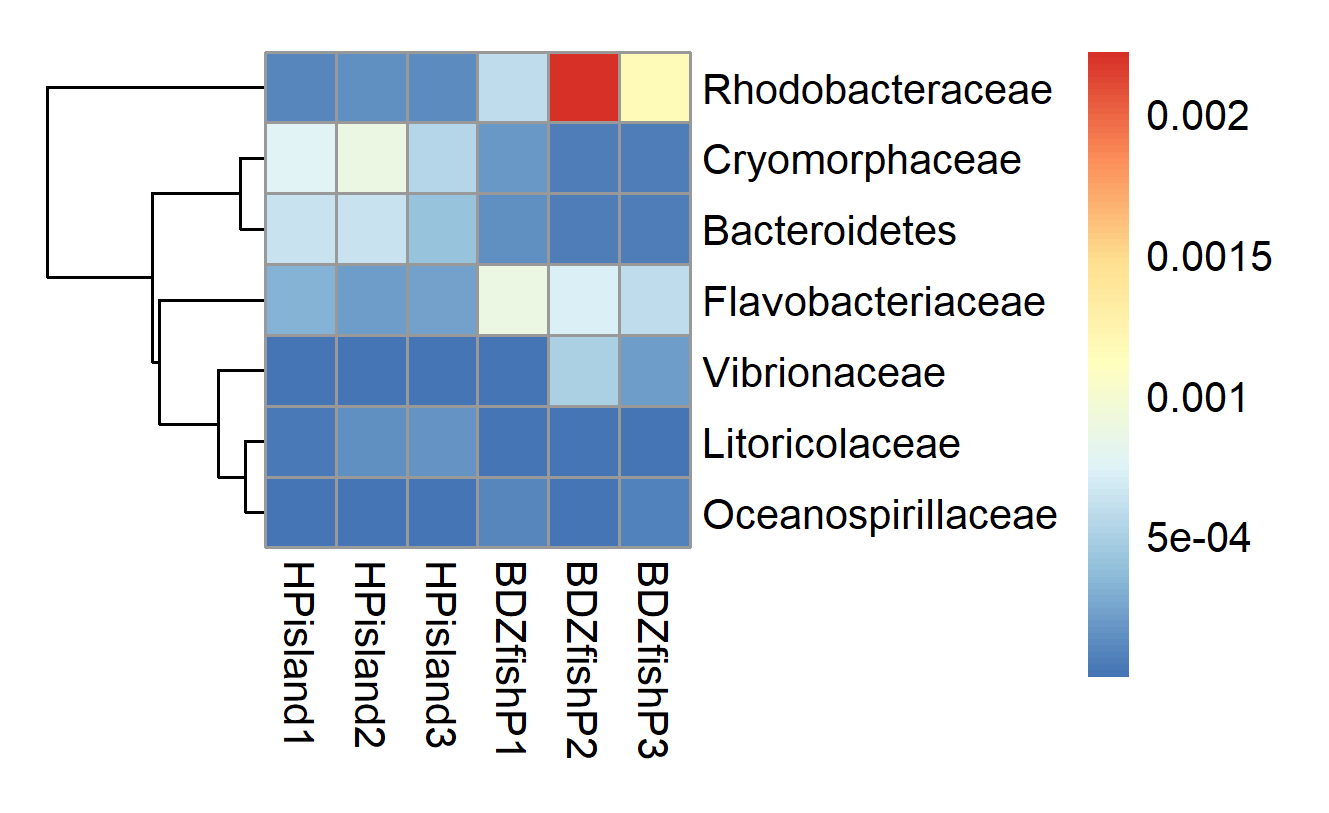


S20 Fig. Taxonomic composition of the GT2 gene (involved in carbohydrate metabolism) in the bacterial communities in Badouzi fishing port (BDZfishP1, BDZfishP2, BDZfishP3) and Heping Island (HPisland1, HPisland2, HPisland3).


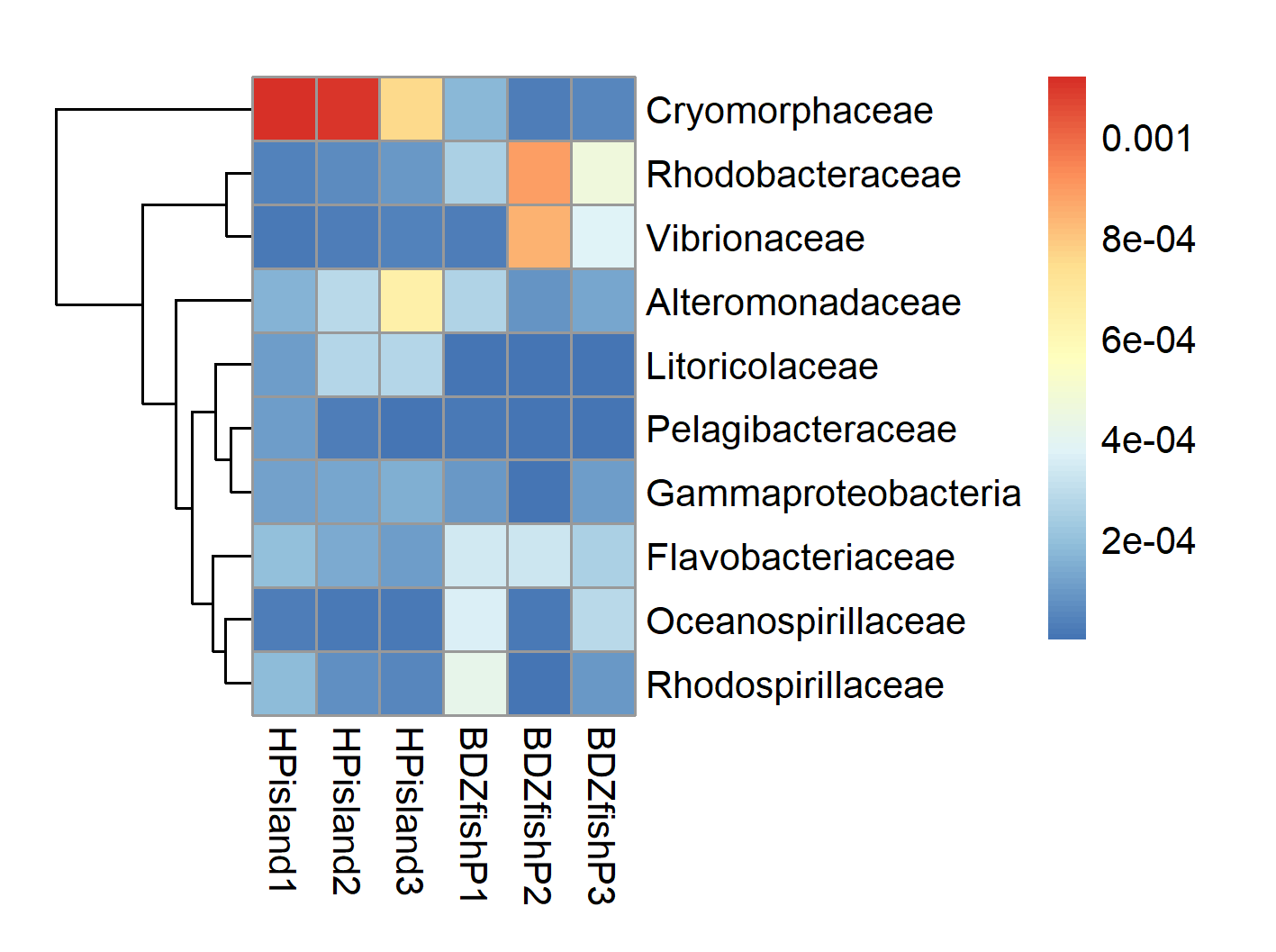


S21 Fig. Taxonomic composition of the GT4 gene (involved in carbohydrate metabolism) in the bacterial communities in Badouzi fishing port (BDZfishP1, BDZfishP2, BDZfishP3) and Heping Island (HPisland1, HPisland2, HPisland3).


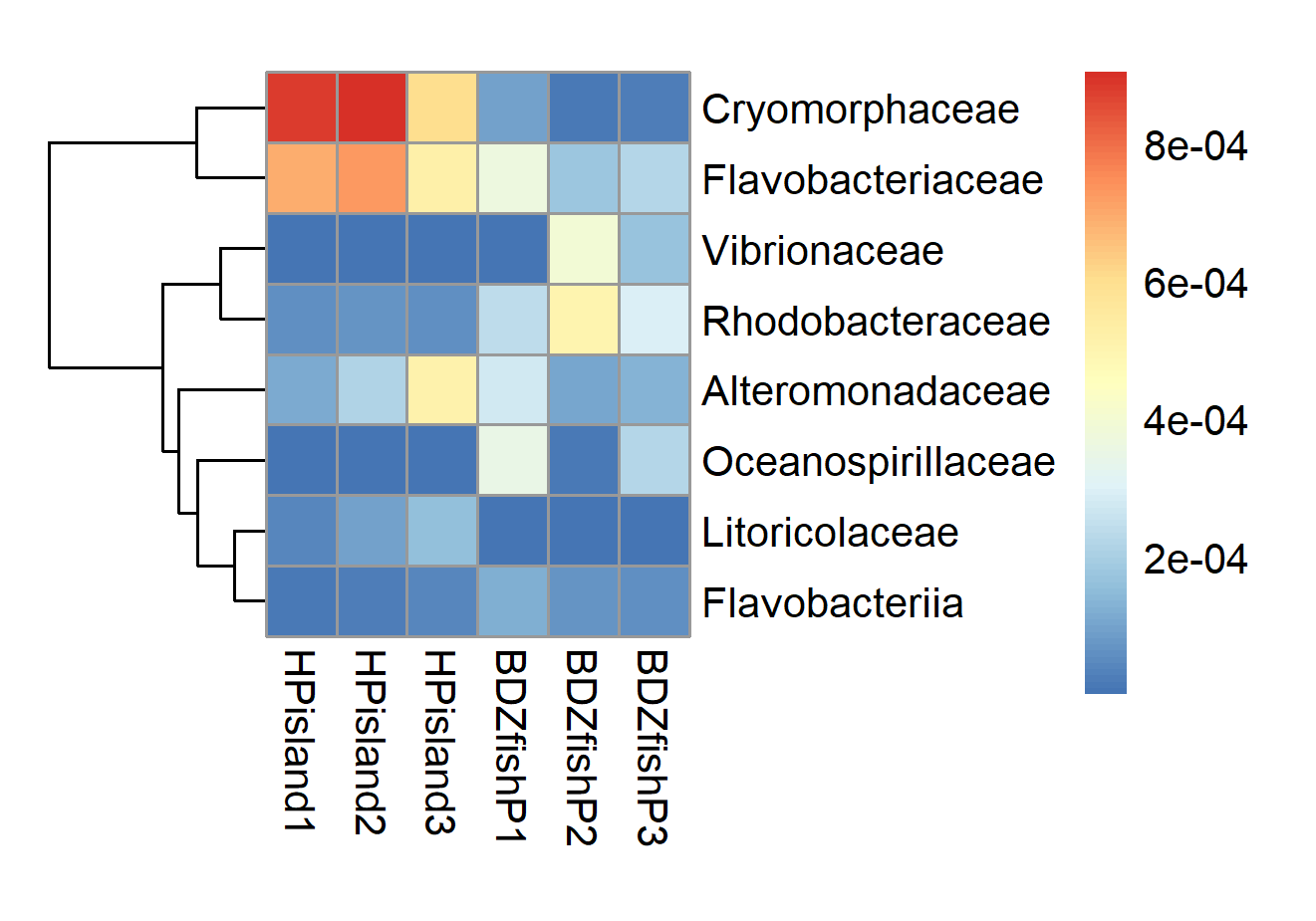


S22 Fig. Taxonomic composition of the GH13 gene (involved in carbohydrate metabolism) in the bacterial communities in Badouzi fishing port (BDZfishP1, BDZfishP2, BDZfishP3) and Heping Island (HPisland1, HPisland2, HPisland3).


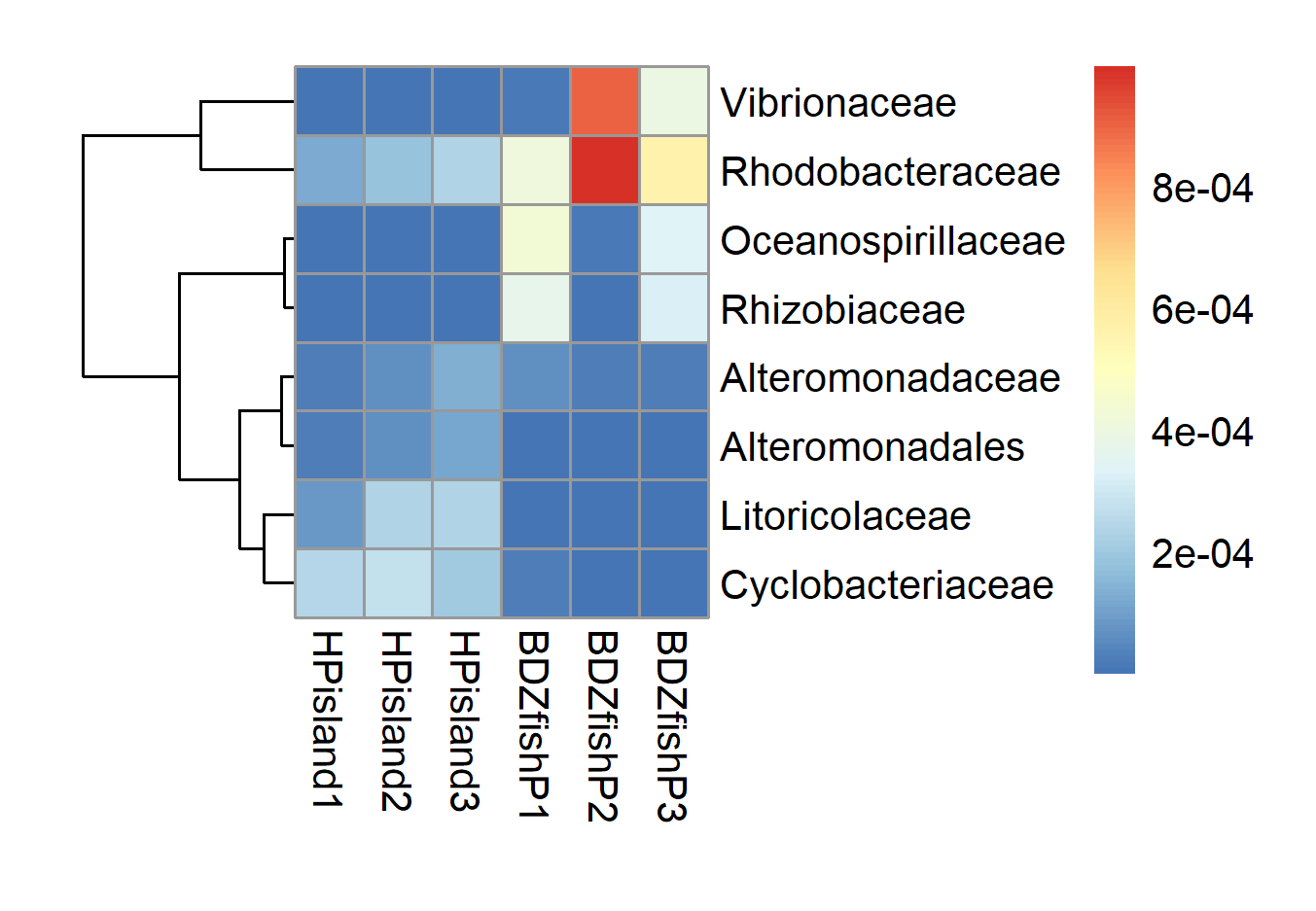


S23 Fig. Taxonomic composition of the GH23 gene (involved in carbohydrate metabolism) in the bacterial communities in Badouzi fishing port (BDZfishP1, BDZfishP2, BDZfishP3) and Heping Island (HPisland1, HPisland2, HPisland3).


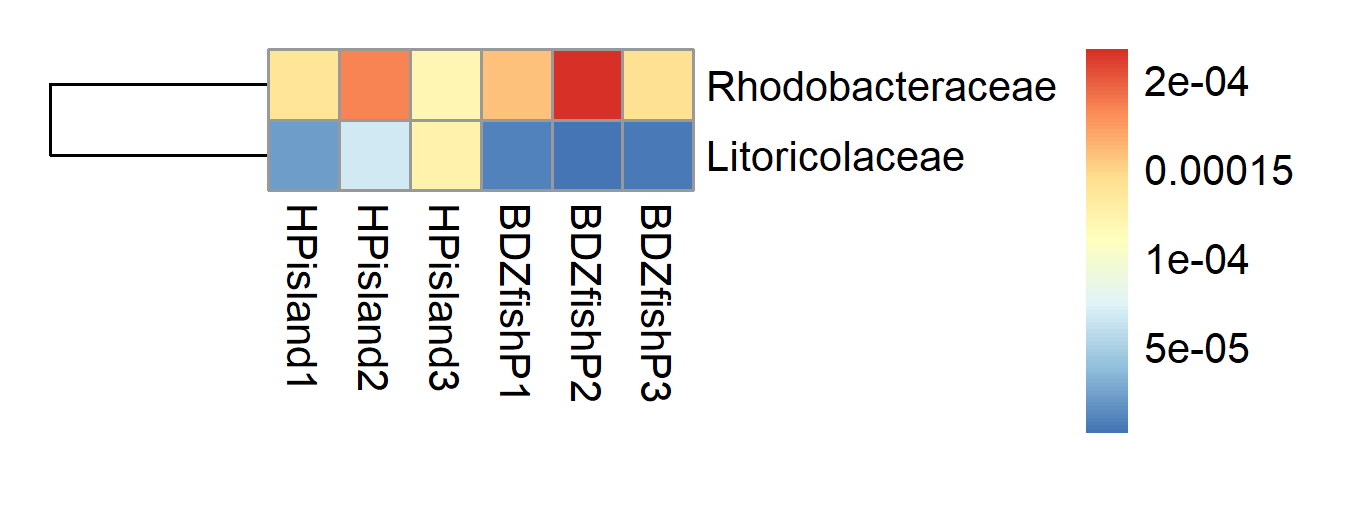


S24 Fig. Taxonomic composition of the *pstB* gene (as the component of ABC transporters) in the bacterial communities in Badouzi fishing port (BDZfishP1, BDZfishP2, BDZfishP3) and Heping Island (HPisland1, HPisland2, HPisland3).


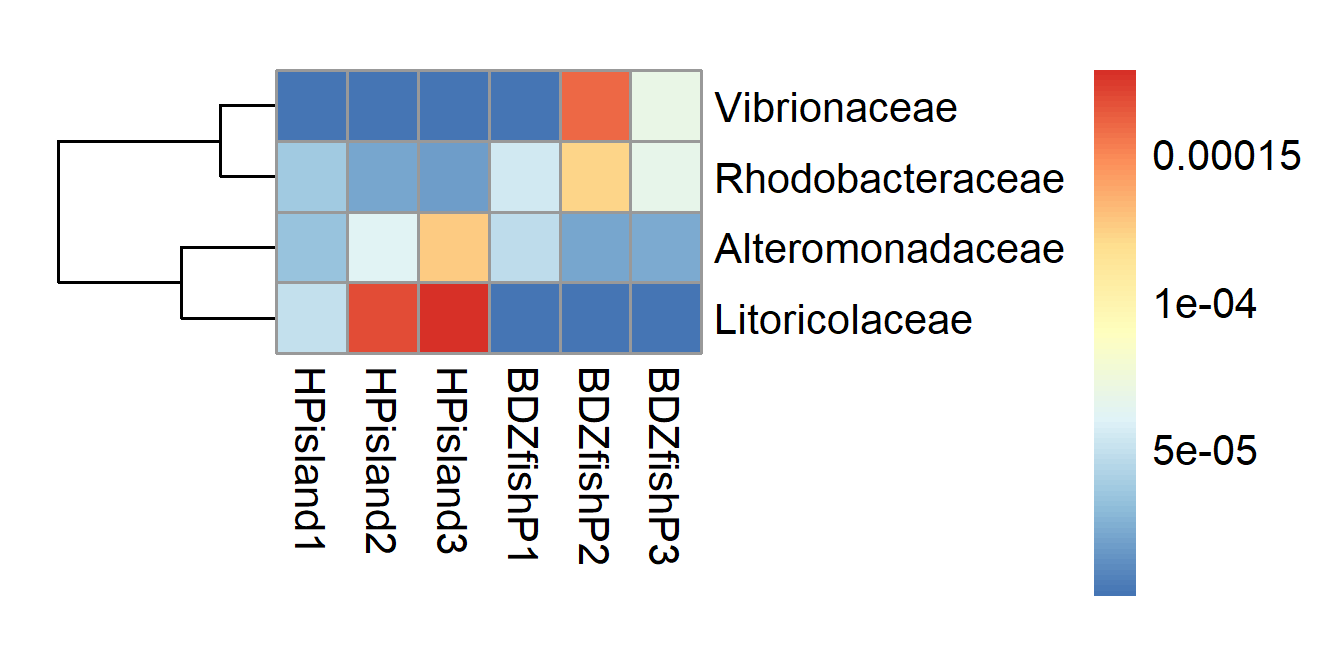


S25 Fig. Taxonomic composition of the *pstC* gene (as the component of ABC transporters) in the bacterial communities in Badouzi fishing port (BDZfishP1, BDZfishP2, BDZfishP3) and Heping Island (HPisland1, HPisland2, HPisland3).


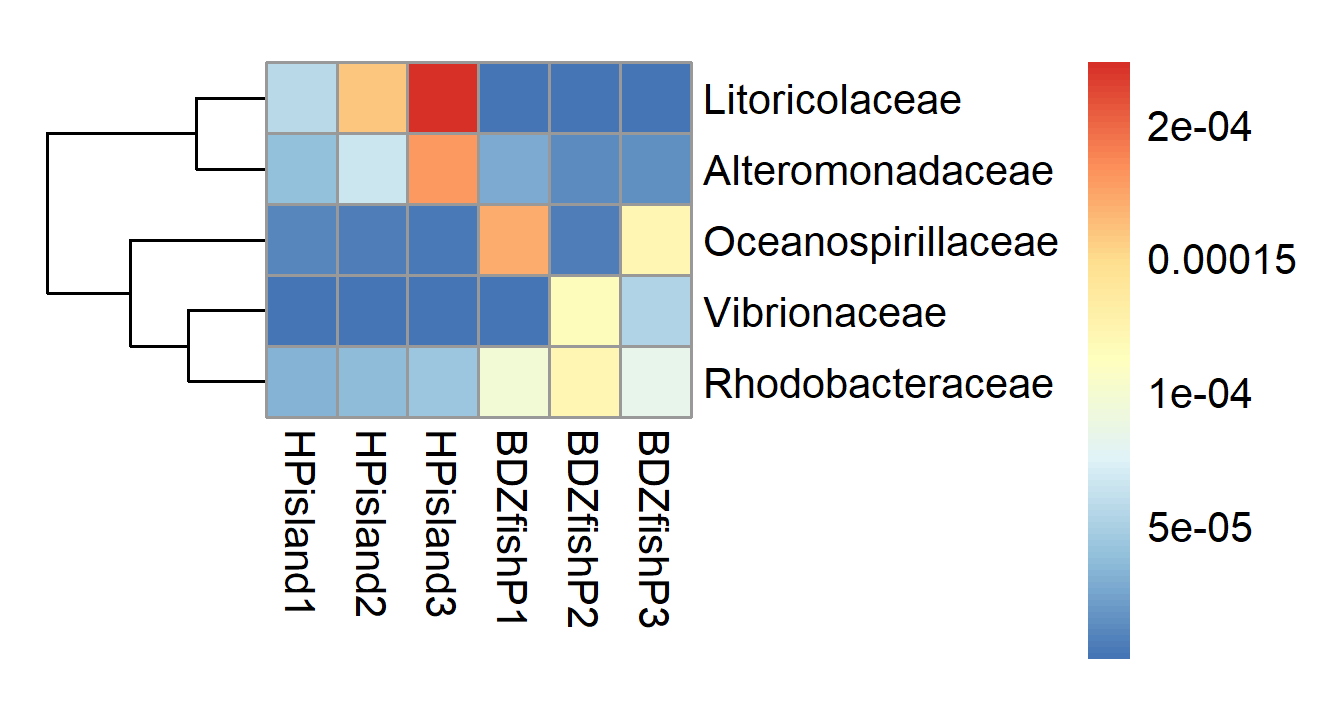


S26 Fig. Taxonomic composition of the *ccmC* gene (as the component of ABC transporters) in the bacterial communities in Badouzi fishing port (BDZfishP1, BDZfishP2, BDZfishP3) and Heping Island (HPisland1, HPisland2, HPisland3).


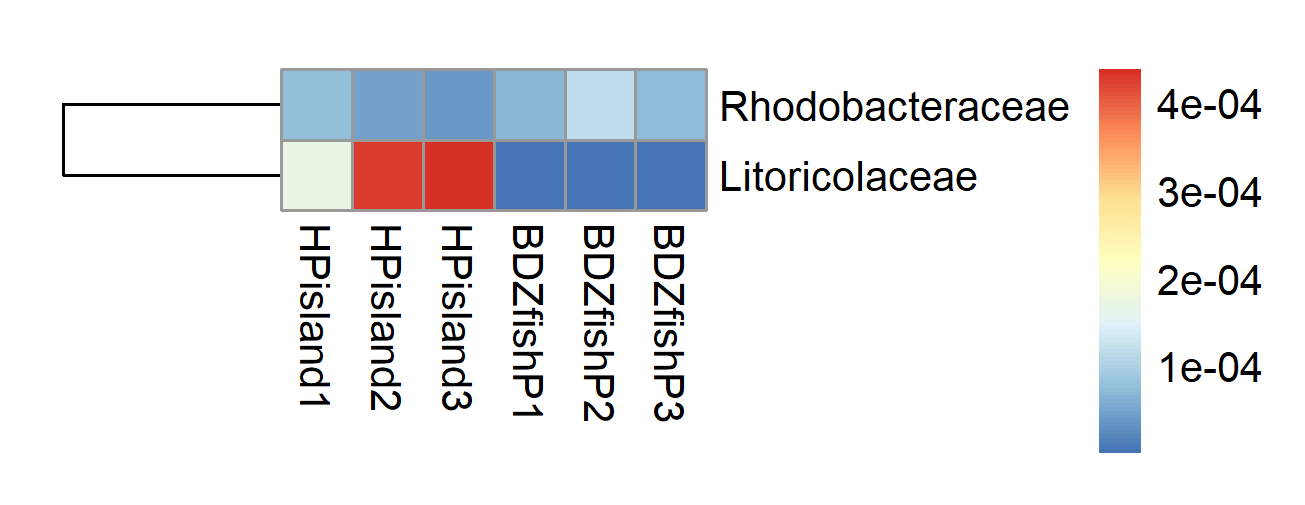


S27 Fig. Taxonomic composition of the *bmpA* gene (as the component of ABC transporters) in the bacterial communities in Badouzi fishing port (BDZfishP1, BDZfishP2, BDZfishP3) and Heping Island (HPisland1, HPisland2, HPisland3).


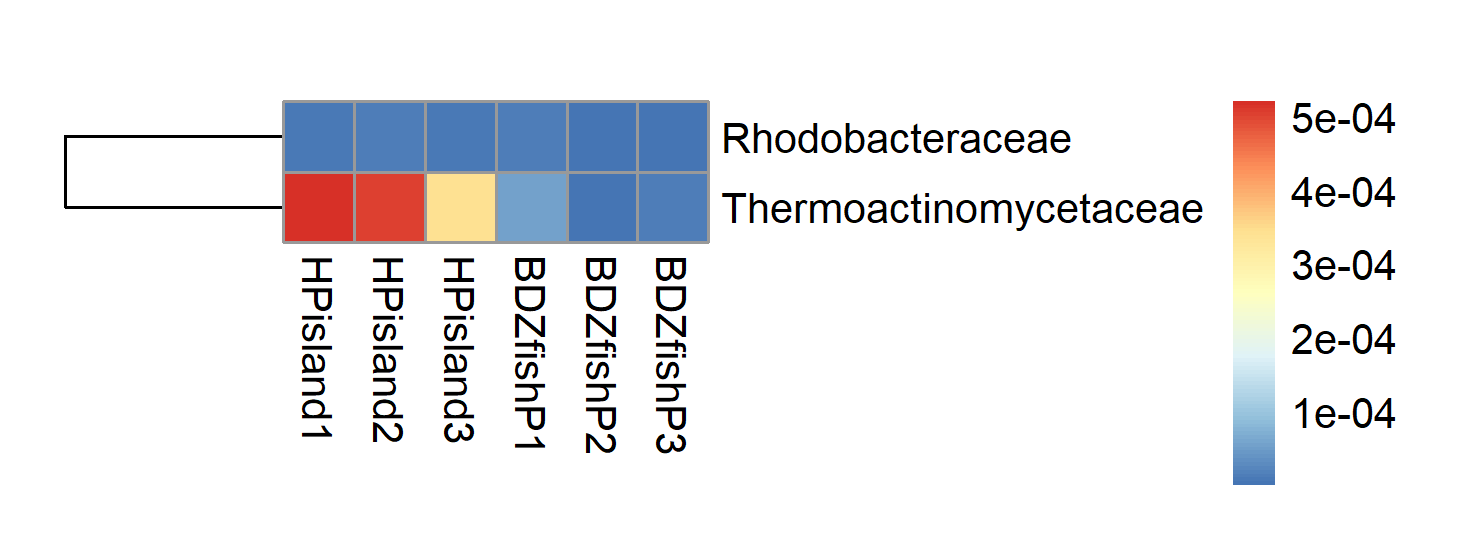


S28 Fig. Taxonomic composition of the *troA* gene (as the component of ABC transporters) in the bacterial communities in Badouzi fishing port (BDZfishP1, BDZfishP2, BDZfishP3) and Heping Island (HPisland1, HPisland2, HPisland3).


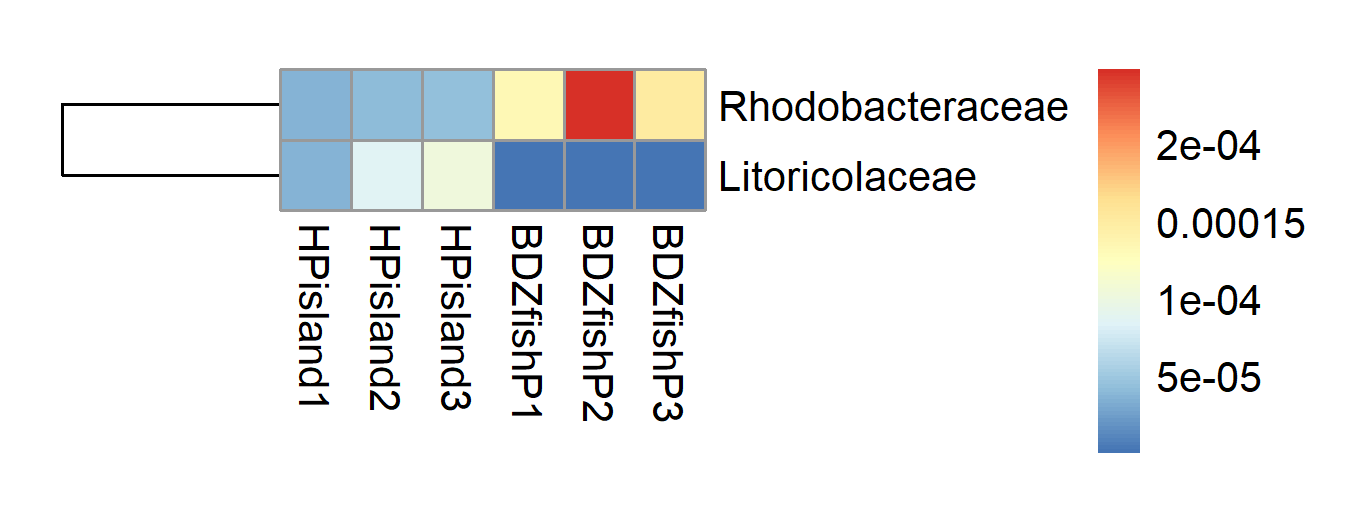


S29 Fig. Taxonomic composition of the *livM* gene (as the component of ABC transporters) in the bacterial communities in Badouzi fishing port (BDZfishP1, BDZfishP2, BDZfishP3) and Heping Island (HPisland1, HPisland2, HPisland3).


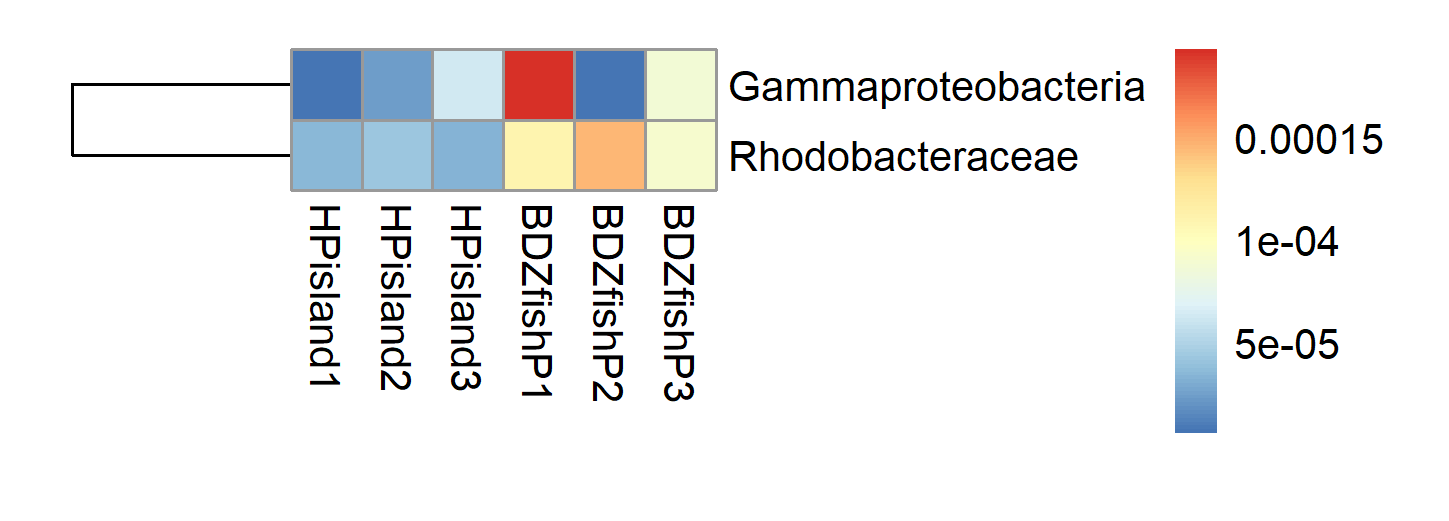


S30 Fig. Taxonomic composition of the *livG* gene (as the component of ABC transporters) in the bacterial communities in Badouzi fishing port (BDZfishP1, BDZfishP2, BDZfishP3) and Heping Island (HPisland1, HPisland2, HPisland3).


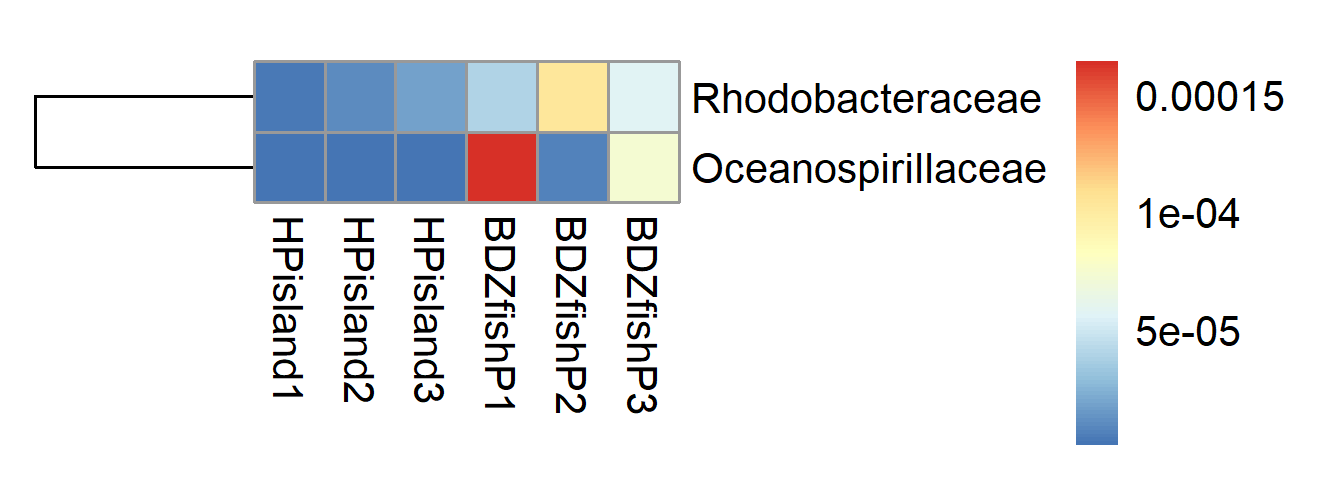


S31 Fig. Taxonomic composition of the genes conferring ansamycin resistance in the bacterial communities in Badouzi fishing port (BDZfishP1, BDZfishP2, BDZfishP3) and Heping Island (HPisland1, HPisland2, HPisland3).


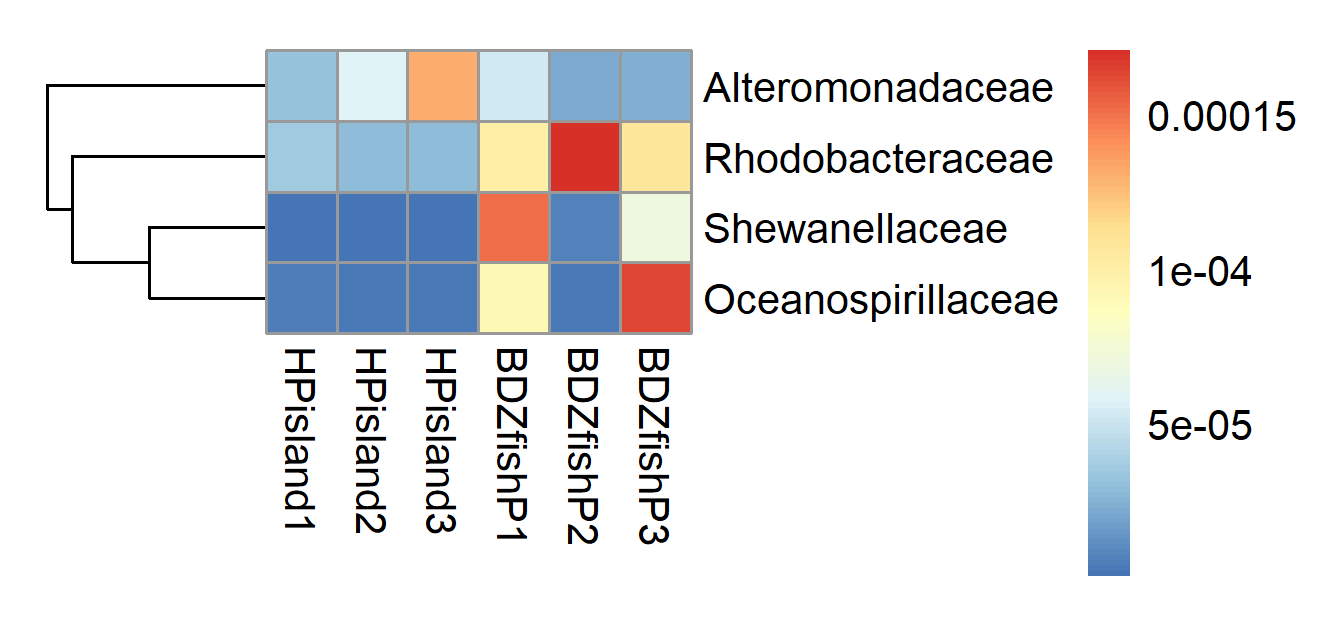


S32 Fig. Taxonomic composition of the genes conferring nitroimidazole resistance in the bacterial communities in Badouzi fishing port (BDZfishP1, BDZfishP2, BDZfishP3) and Heping Island (HPisland1, HPisland2, HPisland3).


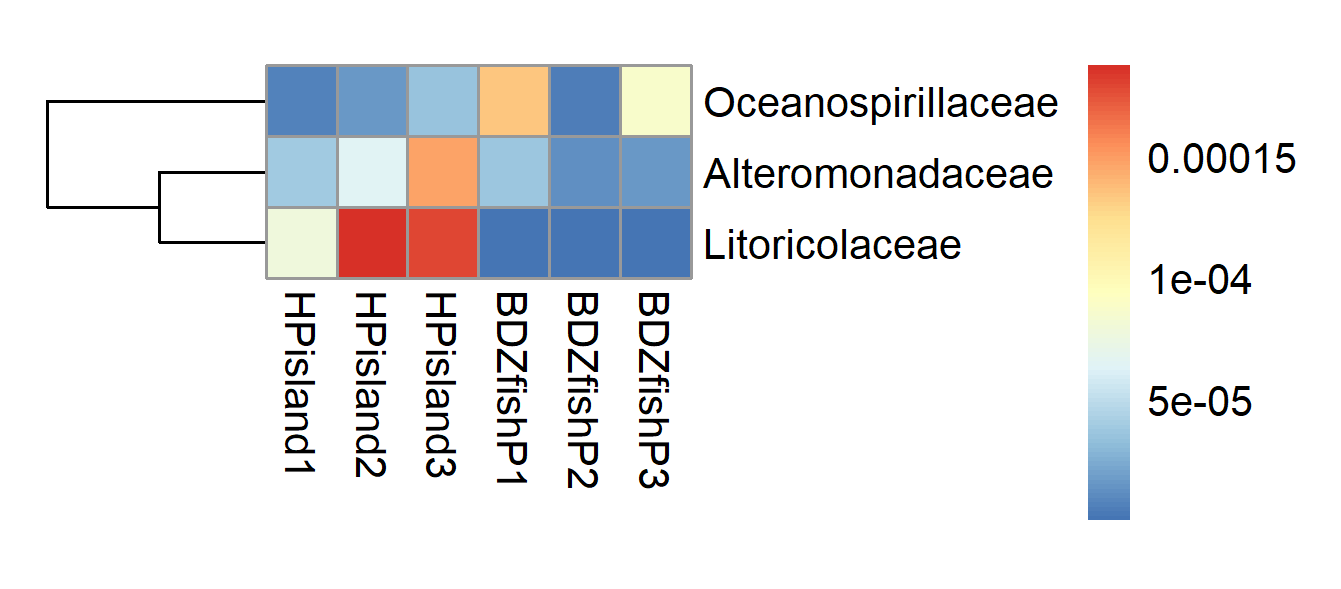


S33 Fig. Taxonomic composition of the genes conferring diaminopyrimidines resistance in the bacterial communities in Badouzi fishing port (BDZfishP1, BDZfishP2, BDZfishP3) and Heping Island (HPisland1, HPisland2, HPisland3).


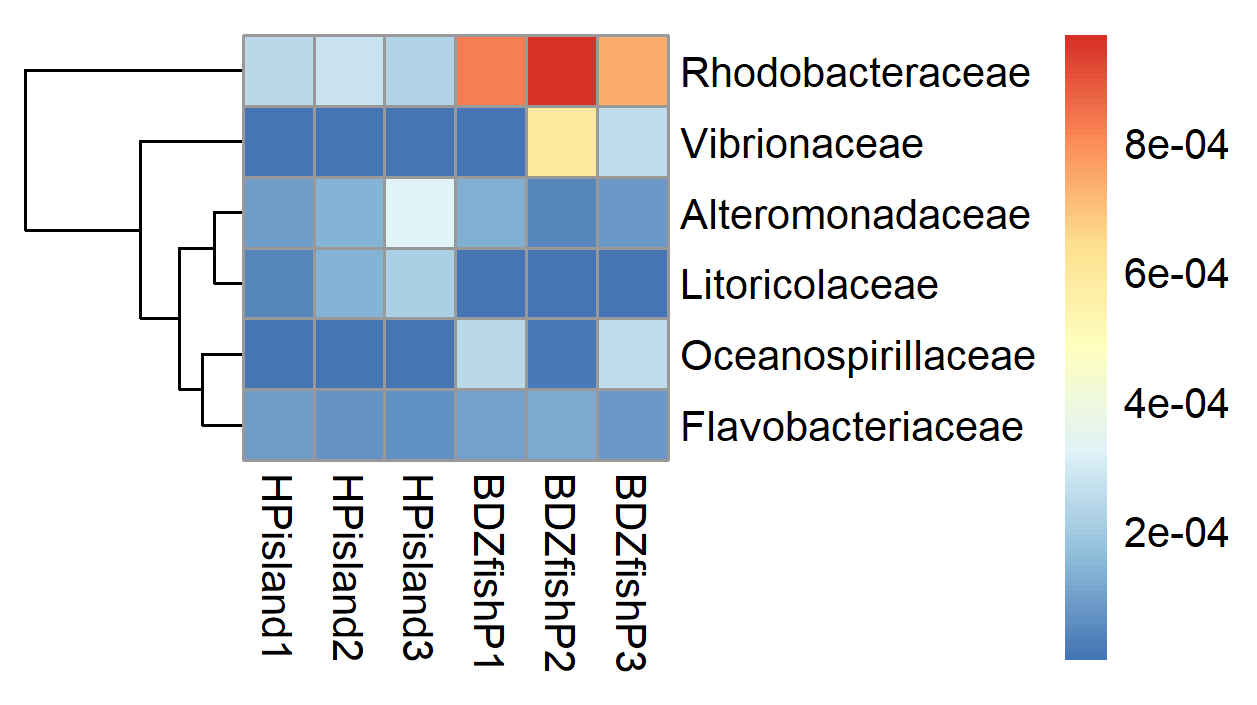


S34 Fig. Taxonomic composition of the genes conferring multimetal tolerance in the bacterial communities in Badouzi fishing port (BDZfishP1, BDZfishP2, BDZfishP3) and Heping Island (HPisland1, HPisland2, HPisland3).


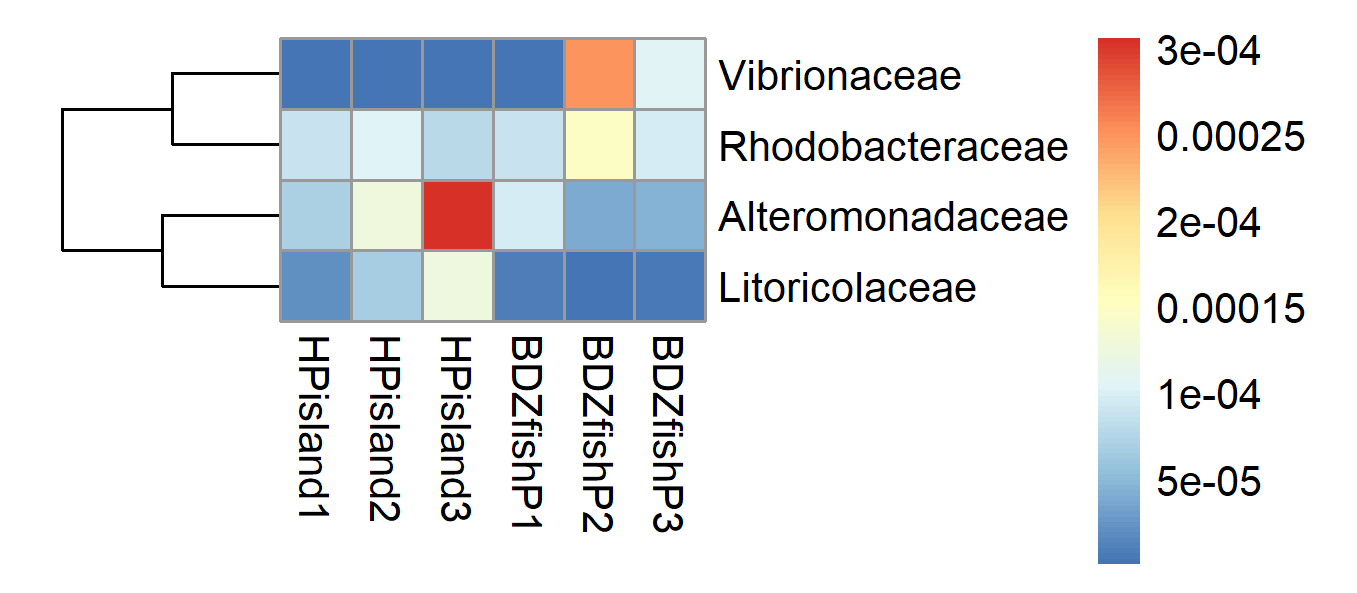


S35 Fig. Taxonomic composition of the genes conferring arsenic tolerance in the bacterial communities in Badouzi fishing port (BDZfishP1, BDZfishP2, BDZfishP3) and Heping Island (HPisland1, HPisland2, HPisland3).


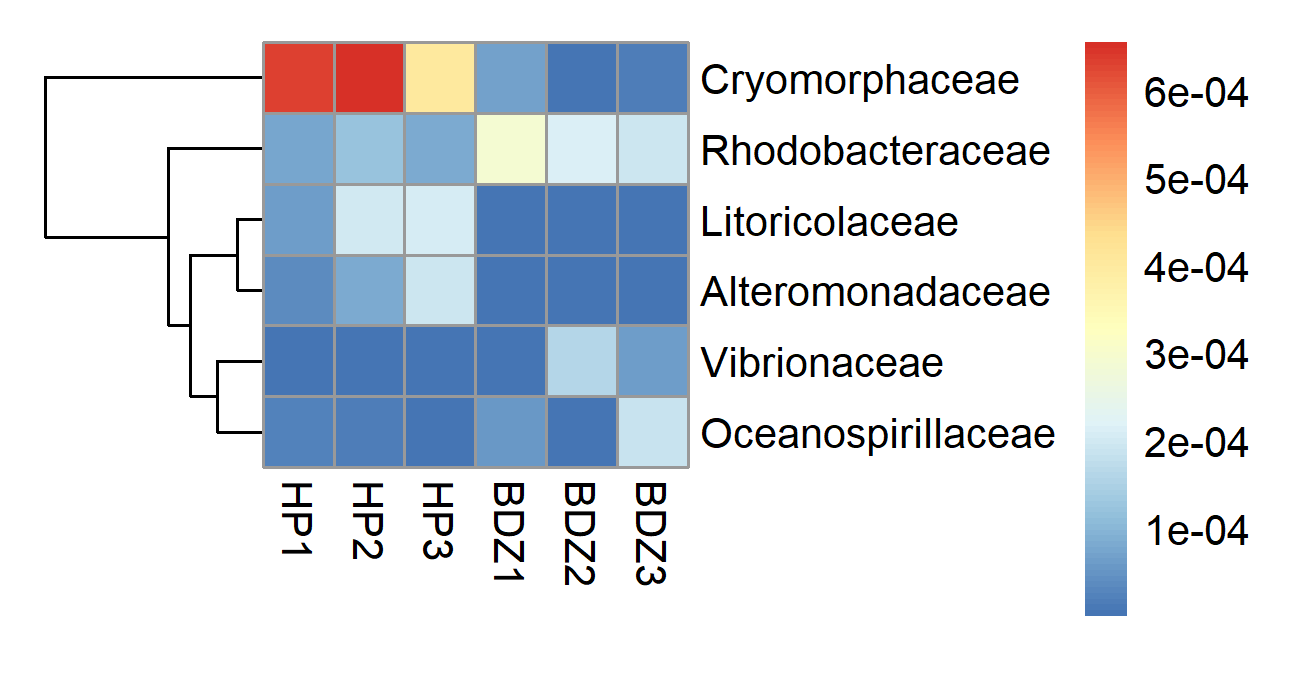


S36 Fig. Taxonomic composition of the genes conferring copper metal tolerance in the bacterial communities in Badouzi fishing port (BDZfishP1, BDZfishP2, BDZfishP3) and Heping Island (HPisland1, HPisland2, HPisland3).


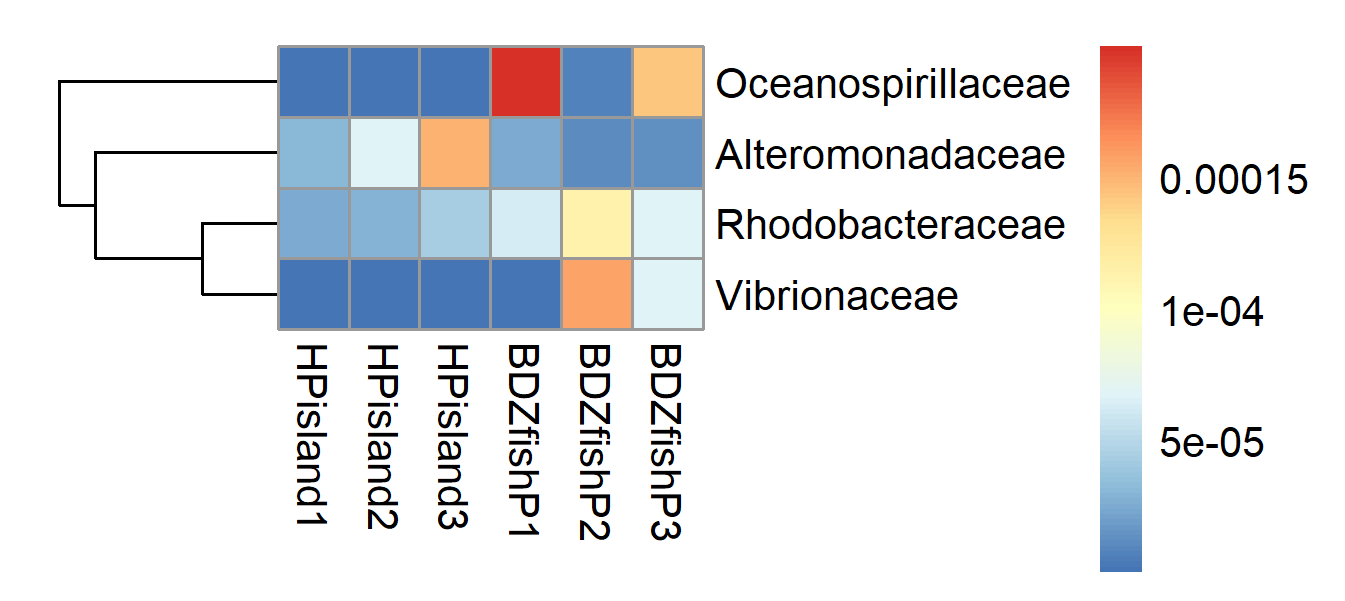


S37 Fig. Taxonomic composition of the genes conferring iron tolerance in the bacterial communities in Badouzi fishing port (BDZfishP1, BDZfishP2, BDZfishP3) and Heping Island (HPisland1, HPisland2, HPisland3).


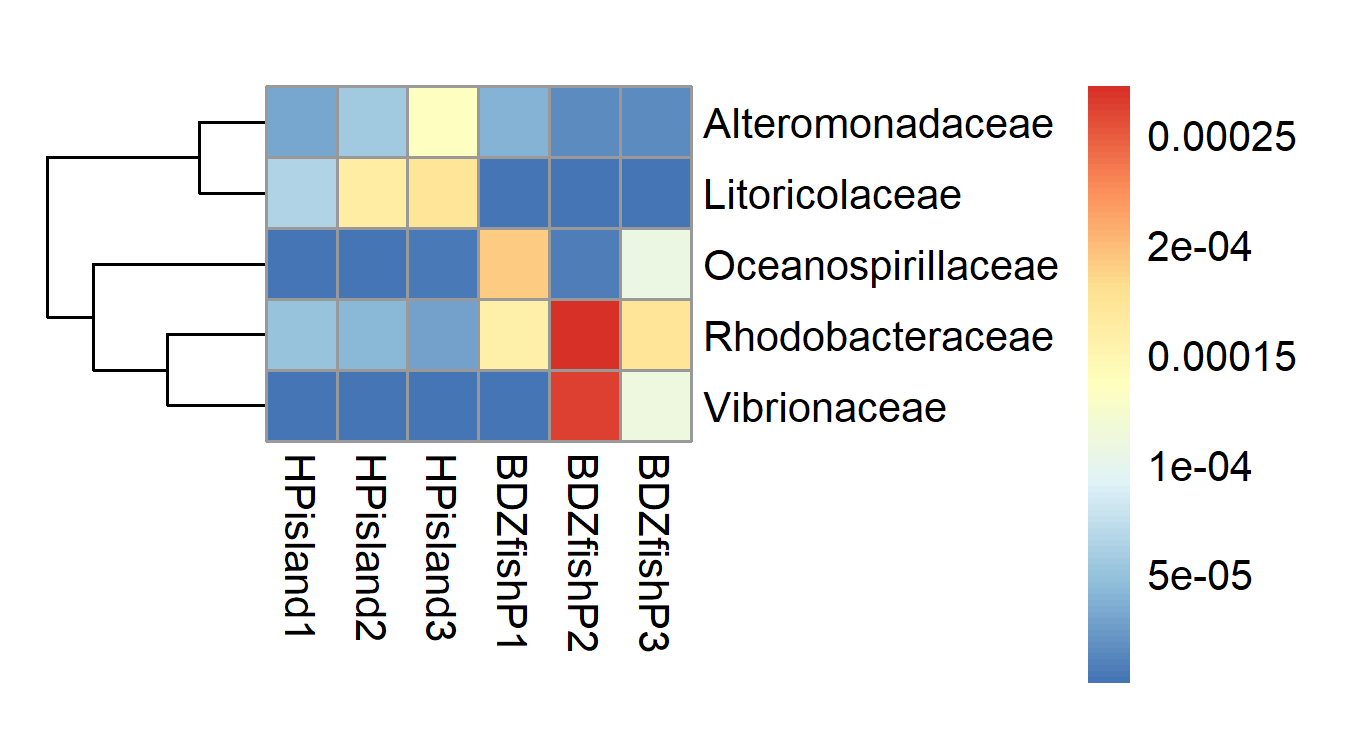


S38 Fig. Taxonomic composition of the genes conferring chromium tolerance in the bacterial communities in Badouzi fishing port (BDZfishP1, BDZfishP2, BDZfishP3) and Heping Island (HPisland1, HPisland2, HPisland3).


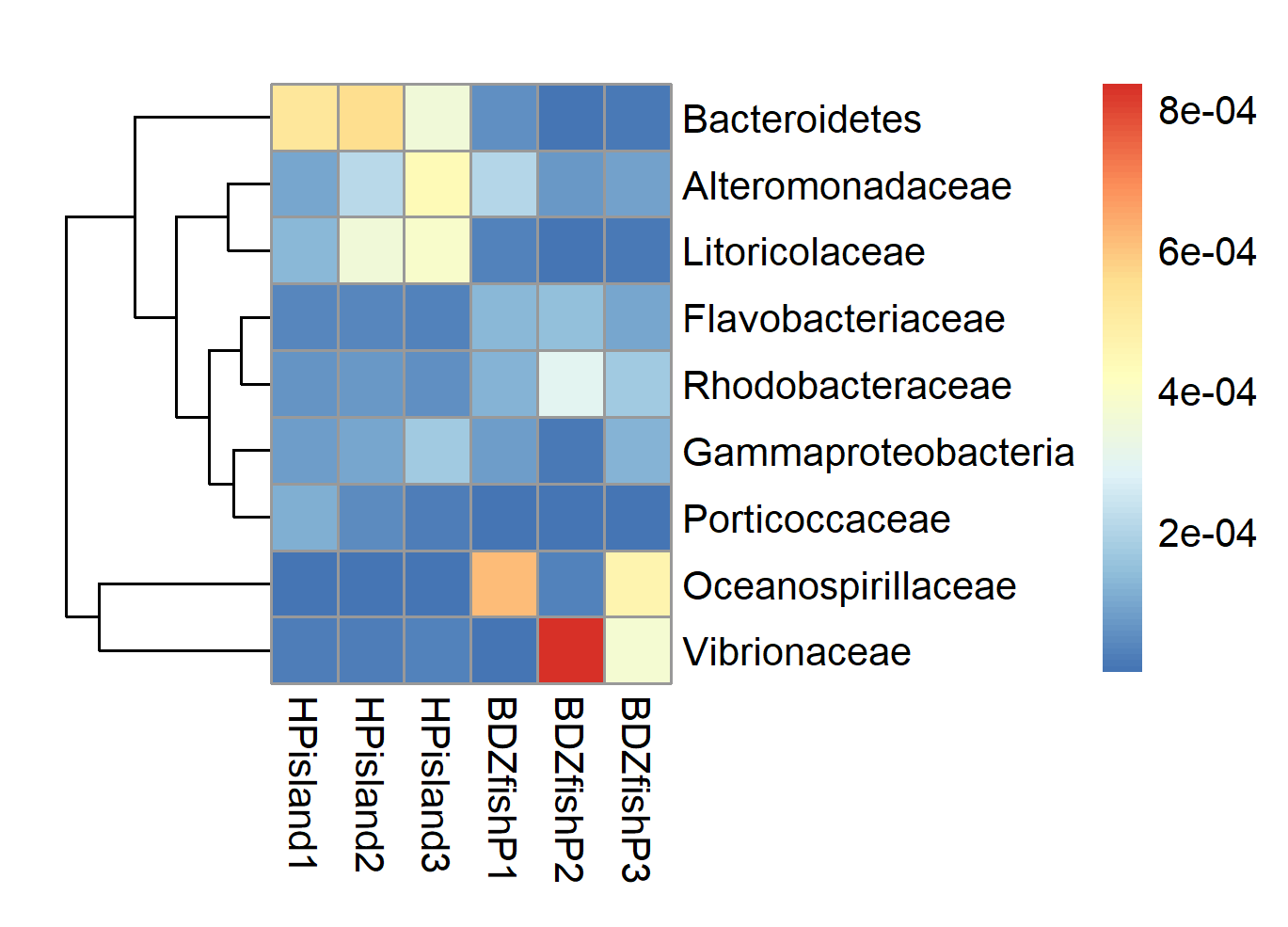


S39 Fig. Taxonomic composition of the genes encoding virulence factor LOS (CVF494) in the bacterial communities in Badouzi fishing port (BDZfishP1, BDZfishP2, BDZfishP3) and Heping Island (HPisland1, HPisland2, HPisland3).


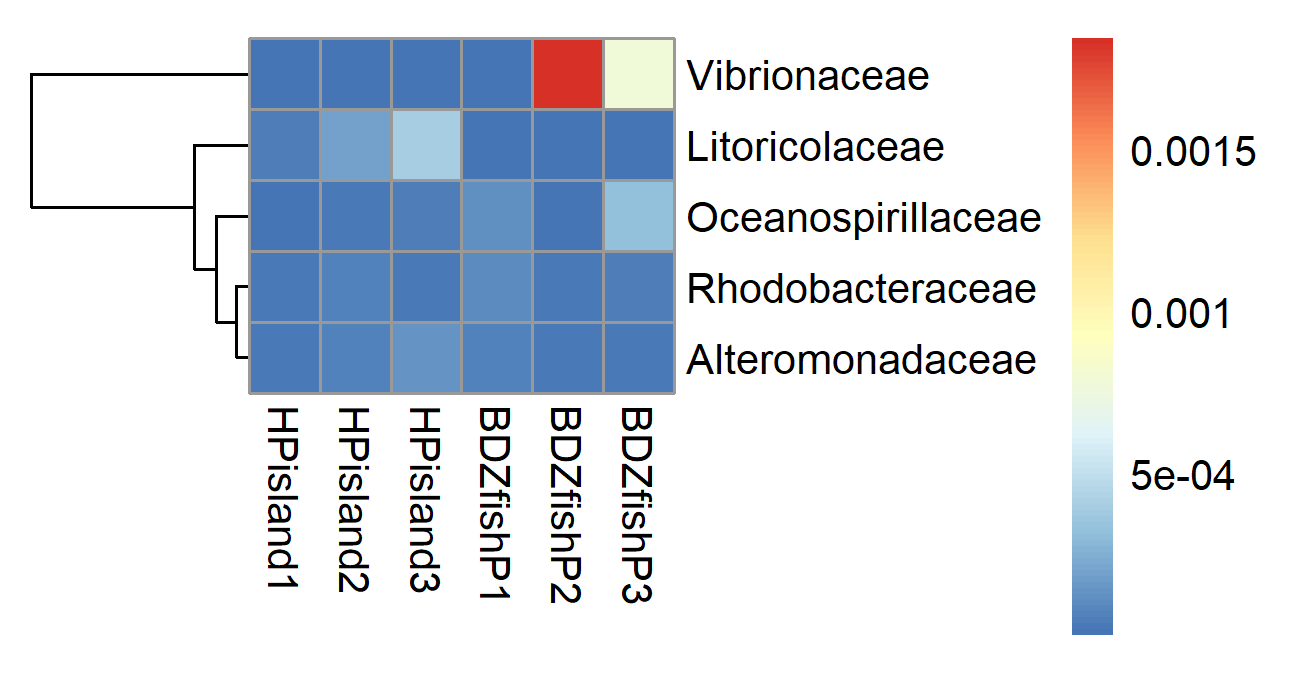


S40 Fig. Taxonomic composition of the genes encoding virulence factor ND (AI144) in the bacterial communities in Badouzi fishing port (BDZfishP1, BDZfishP2, BDZfishP3) and Heping Island (HPisland1, HPisland2, HPisland3).


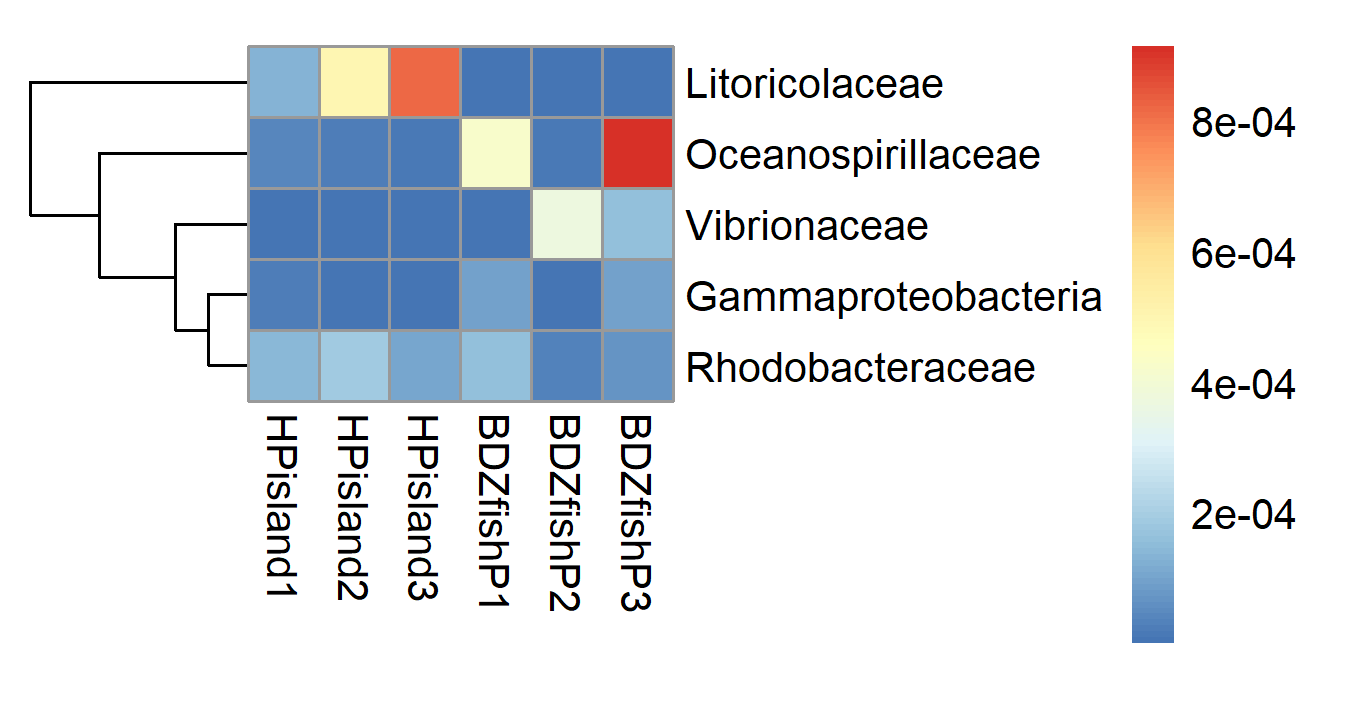


S41 Fig. Taxonomic composition of the genes encoding virulence factor VF0273 in the bacterial communities in Badouzi fishing port (BDZfishP1, BDZfishP2, BDZfishP3) and Heping Island (HPisland1, HPisland2, HPisland3).
